# The Economic Value of Coastal Amenities: Evidence from Beach Capitalization Effects in Peer-to-Peer Markets

**DOI:** 10.1007/s10640-022-00735-5

**Published:** 2022-10-01

**Authors:** David Boto-García, Veronica Leoni

**Affiliations:** 1 Department of Economics, University of Oviedo, Oviedo, Spain; 2 Center for Advanced Studies in Tourism, University of Bologna, Via Angherá 22, 47921 Rimini, Italy; 3 Department of Applied Economics, University of the Balearic Islands, Palma, Spain

**Keywords:** Hedonic pricing, coastal amenities, Capitalization effects, Peer-to-peer markets, Distance decay

## Abstract

Coastal amenities are public goods that represent an important attraction for tourism activities. This paper studies the capitalization effects of beach characteristics using hedonic pricing methods. We examine the implicit economic value of several beach characteristics like sand type, width, longitude, accessibility, or frontage in the Airbnb rental market. Using data for 16,663 Airbnb listings located in 67 municipalities of the Balearic Islands (Spain) during the summer of 2016, together with detailed information about the attributes of 263 beaches, our modelling approach considers interaction terms between the beach amenities and distance to the closest beach. Controlling for a set of listings’ structural characteristics, host attributes and municipality fixed effects, we find that Airbnb guests attach economic value to beach length, the presence of vegetation, the type of coastal frontage and beach accessibility and exclusivity. However, there is no evidence of capitalization effects associated with beach width or the type of sand.

**Supplementary Information:**

The online version contains supplementary material available at 10.1007/s10640-022-00735-5.

## Introduction

Coastal amenities are important attraction factors for coastal areas, especially for those regions specialized in tourism activities. As shown by Onofri and Nunes (2013), tourists choose coastal destinations because they have strong preferences for beach characteristics. The marine ecosystem quality of coastal areas is therefore a significant predictor of tourism flows and revenues (Otrachshenko and Bosello 2017; Spalding et al. 2017), which in turn causes large and significant long-run local economic gains in terms of employment and GDP (Faber and Gaubert 2019). Due to the expected sea level rise caused by climate change, many coastal areas and beaches are at a high risk of erosion. According to the sixth report by the Intergovernmental Panel on Climate Change (IPCC 2021), sea level is expected to rise up to 81 cm along the Spanish coastline in the next 80 years. The identification of the welfare effects of coastal amenities is therefore economically relevant for the appropriate development of policy interventions (Gopalakrishnan et al. 2016a; Parsons et al. 2013), especially in tourism-led economies.

Hedonic pricing functions have been widely used to study consumers’ willingness to pay for a variety of local environmental amenities in many different settings (e.g., Chay and Greenstone 2005; Franco and Macdonald 2018). Typically, residential property transaction prices are used to measure the ‘capitalization effects’ of proximity to environmental amenities, which inform about the economic value of non-marketed goods. Previous research has shown that the quality of nearby coastal areas generates substantial price premiums on residential housing values because consumers value aspects like water quality (Walsh et al. 2017), water view (Lansford and Jones 1995), beach quality (Landry and Hindsley 2011) or beach width (Landry et al. 2021). However, for tourism development, a proper understanding of tourists’ preferences over coastal amenities seems even more relevant because coastal attractiveness is a key driver of inbound tourists’ destination choices and beach visitation (Pascoe 2019). In this sense, whereas residents’ preferences for coastal attributes are widely documented, less is known yet about tourists’ preferences for beach characteristics.

This paper studies the capitalization effects of a large set of coastal amenities. We apply the hedonic pricing method to estimate the implicit prices of several beach characteristics like sand type, width, longitude, accessibility or coastal frontage in the Airbnb rental market. Some works in the tourism literature have analysed the economic value of sea view or beach attributes using hotel prices (Fleischer 2012; Rigall-i-Torrent and Fluvià 2011; Rigall-i-Torrent et al. 2011). However, to the best of our knowledge, there are no studies on how beach amenities capitalize into the prices of peer-to-peer markets.^1^ Airbnb stands nowadays as the leading online marketplace for peer-to-peer accommodation. It has been shown to be a relevant competitor for traditional accommodations (Zervas et al. 2017; Farronato and Fradkin 2022) because of offering different services and experiences to tourists, being also generally cheaper (Tussyadiah and Pesonen 2016). Despite the vast literature on Airbnb hedonic pricing (e.g., Voltes-Dorta and Sánchez-Medina 2020; Moreno-Izquierdo et al. 2020; Casamatta et al. 2022), the economic value of coastal amenities has been overlooked to date in this market segment.

We use data for 16,663 Airbnb listings located in 67 municipalities in the Balearic Islands (Spain) in the summer of 2016. The Balearic Islands is a relevant case study because of being a well-known destination specialized in sun and beach (mass) tourism for whom the tourism sector is an important economic driver (Ginard-Bosch and Ramos-Martín 2016).^2^ These islands are of additional interest because of the concomitant presence of high recreation values, poor protection status and high erosion risk (Ghermandi 2015). We combine data on Airbnb transaction daily rates, host attributes (e.g., number of listings on property, experience as a host, etc.) as a proxy of potential market power and listing structural characteristics (e.g., size, type of property, etc.) with detailed information about the attributes of 263 beaches in the islands. We match each Airbnb listing with the closest beach, so each property is vis-à-vis matched with a beach in our dataset. Since listings are sparsely located throughout the territory, some listings are very close to the beach whereas others are quite distant. In this respect, the hedonic pricing literature has documented a distance decay effect in the contribution of environmental amenities to property values (Lansford and Jones 1995; Gibbons et al. 2014; Landry and Hindsley 2011; Athukorala et al. 2019; Landry et al. 2021). We exploit listings’ closeness to the beach as an indicator of exposure to different beach amenities. Therefore, our modelling approach incorporates interaction terms between the beach attributes and the distance to the closest beach in the hedonic equation to properly estimate the price premiums of coastal amenities. This allows us to uncover edge and proximity effects.

Conditional on an array of structural characteristics, host features and municipality fixed effects, we document that Airbnb guests value the length of the beach, the presence of vegetation, the type of coastal frontage and whether the beach is in an urban environment. However, there is no evidence of capitalization effects associated with beach width, the type of sand or the presence of protected natural spaces in the beach. Interestingly, beaches with a difficult access on foot convey a price premium of 16.2%, which could be associated with preference for beaches with low occupancy.

The contribution of the paper is twofold. First, unlike previous research on the implicit value of coastal amenities that mainly focus on beach width (Landry and Hindley 2011; Landry et al. 2021) or a single environmental attribute (Leggett and Bockstael 2000; Lutzenhiser and Netusil 2001), we estimate the gradient of Airbnb prices with respect to a wide set of beach attributes, separately. In doing so, we examine the economic value of some amenities that have been neglected so far like the type of coastal frontage and beach accessibility. From this perspective, the paper follows the lines of Gibbons et al. (2014), although in a different context. Importantly, to capture potential distance-decay effects, the implicit prices of attributes are allowed to be moderated by the distance to the shoreline. Unlike other studies that restrict the sample to properties within certain thresholds (Landry and Hindsley 2011; Walsch et al. 2017; Catma 2020), we expand the recipients of beach amenity value to properties both in the surroundings of the shoreline and in inland locations within the islands.

Second, we provide the first empirical characterization of the impact of coastal amenities on daily rates in Airbnb accommodations. The analysis of capitalization effects in the peer-to-peer rental market is convenient for at least two reasons. Firstly, hotels generally concentrate around the coast (Marco-Lajara et al. 2016), so the separate identification of the capitalization effects of coastal amenities from other hedonic attributes is cumbersome due to reduced variability. On the contrary, Airbnb listings are more scattered across the islands (Eugenio-Martín et al. 2019), which offers the advantage of a better *ceteris paribus* comparison between properties that are close to the beach and others located further away. In this vein, we consider all Airbnb properties in the islands that have been rented at least once during the study period. Second, compared to the analysis of capitalization effects on the real-estate market, studying short term accommodation rentals entails an additional advantage. Housing selling prices typically conflate the current value of coastal amenities with expectations on the future evolution of beach quality (Bishop and Murphy 2019). In contrast, Airbnb accommodation prices merely reflect consumer preferences for current levels of environmental amenities.

The remainder of the paper is structured as follows. The following section reviews the related literature. In Sect. 3, we present a theoretical characterization based on Rosen’s framework (Rosen 1974) but extended to consider potential hosts’ market power. Section 4 presents and describes the data and the variables used in the analysis. Section 5 outlines the econometric modelling and some empirical aspects to bear in mind. The results are presented and discussed in Sect. 6. Finally, Sect. 7 concludes with a summary of the findings and some implications.

## Literature Review

### The Hedonic Value of Environmental Amenities

A large body of literature has studied how residential property values capitalize the value of environmental amenities. Scholars have estimated hedonic pricing functions that regress transaction prices on a set of local environmental amenities and appropriate controls. This literature has documented relevant price premiums from water quality (Leggett and Bockstael 2000; Walsh et al. 2011, 2017), water clarity (Michael et al. 2000), waterfront view (Brown and Pollakowski 1977; Lansford and Jones 1995), open spaces (Lutzenhiser and Netusil 2001), air quality (Chay and Greenstone 2005) or cultural heritage (Franco and Macdonald 2018). Similarly, other scholars have studied the price discounts associated with bushfires exposure (Athukorala et al. 2019), road noise (Andersson et al. 2010), power plants (Davis 2011), hurricane occurrence risks (Cohen et al. 2021) or closeness to hazardous waste sites (Greenstone and Gallagher 2008).

Rather than focusing on the hedonic value of a specific amenity, other scholars have estimated the separate shadow prices of several environmental attributes. Gibbons et al. (2014) estimate the amenity value associated with proximity to habitats, designated areas, domestic gardens, rivers and other natural amenities in England. They document considerable positive price premiums for gardens, freshwater, flood plain locations and green spaces within the census ward. Liu et al. (2020) analyse the spillover effects on housing prices of ecological lands considering forest, grassland, wetland and cultivated land in China. Using a multilevel hedonic model, they find that forest size, wetland size and a moderate grassland area exert positive and linear effects on house prices.

Climate change is producing a gradual increase in sea level, causing beach erosion and increasing the frequency of coastal flooding. Since beach width is an important attribute for both shore protection and recreation, beach nourishment projects have been undertaken. Given the large costs associated with these projects, another stream of research has focused on estimating the economic value of beach width using residential property prices. For instance, Landry and Hindsley (2011) show that beach and dune widths increase house values but within a 300-m radius from the shore, over which their effects become non-significant. Similarly, Landry et al. (2021) pay attention to the role played by shoreline proximity and potential measurement error problems. They find positive price premiums for beach width and no problems of errors-in-variables. Catma (2020) estimates spatial hedonic regressions and documents that beach width positively influences values of properties located within 633 feet of the shoreline. Gopalakrishnan et al. (2016b) revisit the impact of beach width on house property values studying the potential attenuation bias when the beaches under study have implemented beach replenishment projects. Using IV methods, they find that the capitalization effect of beach width is larger than previously estimated.

Overall, a common finding is that the contribution of coastal amenities to property values diminishes as we move away from the shoreline. That is, consumers’ willingness to pay for coastal amenities is subject to a distance decay pattern (Brown and Pollakowski 1977; Lansford and Jones 1995; Landry and Hindsley 2011; Gibbons et al. 2014; Athukorala et al. 2019; Landry et al. 2021). That is why empirical applications typically restrict the samples to those properties that lie within certain ad hoc distance boundaries.

### The Economic Value of Beach Quality

In the tourism economics literature, the hedonic method has been applied to uncover the implicit prices of accommodation attributes for hotels (e.g., Rigall-i-Torrent and Fluviá 2011), second homes (e.g., Saló and Garriga 2011) and Airbnb listings (e.g., Casamatta et al. 2022). Most of this literature focuses on the hedonic value of intrinsic characteristics and typically control for location through neighbourhood fixed effects. However, although the economic value of the sociodemographic composition of the neighbourhood has started to be recognized (Rigall-i-Torrent et al. 2011; Saló and Garriga 2011; Saló et al. 2014; Moreno-Izquierdo et al. 2020), studies that estimate the economic value of beach amenities are scarce.

One of the first works on the economic value of coastal amenities is Hamilton (2007), who investigates the influence of landscape attributes on the prices of hotels, bed and breakfast and private rooms in 92 districts in Germany. This author finds that districts with open coast charge higher prices, whereas an increase in the length of dikes is associated with lower prices. Beyond this work, most existing studies have focused on the aesthetic value of sea view. Conditional on other hedonic characteristics, Rigall-i-Torrent and Fluviá (2011) and Espinet et al. (2003) document that hotel rates are higher when the hotel locates in front of the beach. Fleischer (2012) goes a step further showing that more important than hotel location is whether the room has a sea view. His estimates point to a 10% price differential between rooms with and without sea view. Similarly, using data for both hotels and second homes, Saló et al. (2014) find a price premium of around 15.7% from beachfront view. They also report a smooth price decrease as the distance between the accommodation and the closest beach increases. In a study on the relationship between second-home prices and neighbourhood amenities, Saló and Garriga (2011) report that prices decrease linearly as the dwelling locates further away from the shoreline.

Overall, the price premium of being close to the beach is widely recognized. However, beaches are highly heterogeneous and less is known yet about the separate capitalization effects of their distinct features. For example, how much do tourists value lodging close to a beach with gold sand? Do they attach value to beach accessibility or the type of frontage? To the authors’ knowledge, Rigall-i-Torrent et al. (2011) is the only study that examines the impact of beach characteristics on hotel prices using data for Catalonia region. They consider beach width, length, degree of urbanization, type of sand, and the availability of services like WC facilities or umbrellas for rent, among others. They show that beachfront location translates into a price premium of around 17% and that prices decrease as distance to the beach increases. Additionally, prices are negatively correlated with beach width but unrelated to beach length. In the current study, we expand their work by focusing on the beach capitalization effects in the Airbnb peer-to-peer rental market, paying attention to the moderating role of distance on amenities’ capitalization.

## Theoretical Framework

### Hedonic Prices Under Perfect Competition

Listings offered on Airbnb can be understood as a bundle of characteristics in the sense of Lancaster (1966) that embed a combination of private and public attributes. Conditional on having decided to stay at an Airbnb accommodation, consumers derive utility from the private characteristics of the listing (e.g., size or the type of building) as well as from its geographic location. In this regard, the public characteristics of the area where the accommodation is placed (such as safety, cleanliness or accessibility to natural amenities) are additional sources of utility. Therefore, consumers’ utility per night stay is expressed as follows:

1 $$U\left( {x, C, Z} \right)$$

where *C* are Airbnb private attributes *c*_*k*_ (for $k = 1, \ldots K$), *Z* is a vector of public goods *z*_*m*_ (for $m = 1, \ldots M$) that characterize the environment where the listing is located (including the coastal amenities), and *x* is a composite good to be consumed during the tourist stay. The utility function is assumed to be monotonically increasing in its three arguments so that $\frac{\partial U}{{\partial X}} > 0,$ $\frac{\partial U}{{\partial C}} > 0$ and that $\frac{\partial U}{{\partial Z}} > 0.$

Consumers choose the listing that maximizes utility subject to the budget constraint:

2 $$x + P = I$$

where the price of the composite good *x* is taken as a numeraire, *P* is the price per night of an Airbnb listing, and *I* is consumer’s disposable income.

From the first-order conditions of the maximization problem we get:

3 $$\begin{aligned} \frac{\partial P}{{\partial c_{k} }} & = \frac{{\frac{\partial U}{{\partial c_{k} }}}}{{\frac{\partial U}{{\partial x}}}} = MRS_{{c_{k} ,x}} \\ \frac{\partial P}{{\partial Z}} & = \frac{{\frac{\partial U}{{\partial z_{m} }}}}{{\frac{\partial U}{{\partial x}}}} = MRS_{{z_{m} ,x}} \\ \end{aligned}$$

Since in equilibrium $MRS_{{z_{m} ,X}} = p_{{z_{k} }}$/$p_{x}$ and $p_{x} = 1,$ it holds that consumers’ decision regarding the quantity of public attribute $z_{m}$ is optimal when the marginal willingness to pay equals the marginal increase in price per change in the attribute *m*. Therefore, consumers’ willingness to pay for the bundle of private and public characteristics embedded in Airbnb listing i (for $i = 1, \ldots ,N$) taking utility and income as given is expressed as:

4 $$WTP_{i} = \theta \left( {c_{1} , c_{2} \ldots ,c_{k} ; z_{1} ,z_{2} \ldots ,z_{m} } \right)_{i}$$

with $\frac{\partial \theta }{{\partial c_{k} }}$ and $\frac{\partial \theta }{{z_{m} }}$ being the marginal willingness to pay for private attribute $c_{k}$ and public attribute $z_{m}$, respectively.^3^

Airbnb listings located in areas with a greater supply of public attributes are expected to be highly priced conditional on the same private characteristics. This greater WTP stems from quasi-rents from product differentiation derived from consumers’ preferences over site-specific public attributes (Taylor and Smith 2000).

Let us for the moment assume Airbnb hosts operate in a competitive market. The price at which a host is willing to supply an additional private characteristic (willingness to accept) equals the marginal cost (including opportunity ones). The total price per night is therefore the sum of the shadow prices of each listing attribute. In equilibrium, consumers’ willingness to pay for listing i equals its market price ($$WTP_{i} = P_{i} )$$ and the marginal willingness to pay for attribute $$c_{k}$$ equals its corresponding shadow price $$\left( {\frac{\partial \theta }{{\partial c_{k} }} = \frac{\partial P}{{\partial c_{k} }}} \right)$$. As a result, the market price of Airbnb listings can be expressed as a function of implicit prices of the private and public characteristics as follows:

5 $$P_{i} = f\left( {C_{i} , Z_{i} } \right)$$

A regression of observed market prices on private and public attributes will therefore provide estimates of the marginal valuation for the different attributes if consumer preferences are homogeneous (Rosen 1974). The error term would capture unobserved variability in prices stemming from unobserved characteristics that are assumed to be uncorrelated with the regressors.

### Hedonic Prices with Market Power

The hedonic price model presented before assumes perfect competition. This is based on the fact that Airbnb originally emerged as an online platform in which non-professional hosts rented their underutilized space (rooms) to peers, generally at lower prices than the ones charged by traditional market-based accommodations. However, several studies have documented a radical change in Airbnb use, with a substantial share of listings currently managed by a reduced number of hosts, who operate close to business firms. These hosts charge higher rates (Gibbs et al. 2018), are more proficient in setting prices (Kwok and Xie 2019) and therefore earn greater revenues (Xie et al. 2021; Casamatta et al. 2022). Since they manage several listings and are usually concentred geographically, they are better able to exploit economies of scale (Li and Srinivasan 2019). This has led to a *professionalization* of Airbnb (Gil and Sequera 2020; Dogru et al. 2020).

Therefore, Airbnb can be understood as a monopolistic competition market as defined by Chamberlin (1933), where hosts face a downward sloping demand curve. Since professional hosts are motivated by profit maximization, they are expected to keep prices close to the perfect competitive equilibrium under a highly elastic demand and to exert market power under an inelastic demand. Since the demand curve is unobserved from the host viewpoint, they must form a belief. Those managing several properties in the same market and with longer experience (professionals) are predicted to have better knowledge about the market conditions, *ceteris paribus*, and therefore better able to assess market demand. Evidence presented in Gunter et al. (2020) and Bibler et al. (2021) show that Airbnb demand is quite inelastic, which allows professionals to increase revenues via price hiking. In this vein, Casamatta et al. (2022) documents that professionals indeed charge larger prices, particularly during the peak season when demand elasticity is lower.

Similar to Harding et al. (2003) and Cotteleer et al. (2008), we assume a set of host characteristics (including the number of properties as a professionalism indicator) are a valid proxy of the parallel shift in the hedonic price function caused by market power. Therefore, the expanded hedonic pricing function in the presence of market power is given by:

6 $$P_{i} = f\left( {C_{i} , Z_{i} , H_{i} } \right) + \epsilon_{i}$$

where $H_{i}$ is a set of host characteristics and $\epsilon_{i}$ is the error term.^4^

## Data

### Case Study

The Balearic archipelago is a well-known tourism-led economy. According to local statistics (IMPACTUR 2014), the tourism industry contributes to 45% of regional GDP and 32% of local employment. With more than 13.6 million of international tourist arrivals in 2019 (FRONTUR 2020), the Balearic Islands are one of the most popular ‘sun and beach’ tourist destinations worldwide. Domestic tourism accounts for about 17% of arrivals, while most international tourists come from Germany, United Kingdom, Italy and France. Its tourism demand is strongly seasonal, with more than 60% of tourist arrivals concentrated during the summer period.

Beaches are among the main attractions for tourism activities in the Balearic Islands which, paradoxically, have contributed to the degradation of the archipelago’s natural resources. As documented in Ghermandi (2015), these islands are characterized by a poor protection status and high erosion risk. Roig-Munar et al. (2019) indicate that the geomorphological and environmental peculiarities of the islands’ coastal ecosystems have not been properly considered by local authorities, which has led to suboptimal conservation policies. This has resulted in a strong exposure to coastal erosion, loss of beach surface and volume, elimination of dune formation and loss of biodiversity, among others (Roig-Munar et al. 2019).

### Data Description

Our analysis uses two types of information: (1) Airbnb listings’ prices, their structural characteristics and host features, and (2) detailed beach amenities. The following paragraphs are devoted to the description of the data sources and variable definition.

#### Airbnb Data

Data on Airbnb listings has been obtained from AirDNA and cover the entire Balearic archipelago (Mallorca, Menorca, Ibiza and Formentera). For the month of August 2016, we have information on daily prices and status (*booked*, *blocked*, *available*) for all the properties listed on Airbnb platform in the islands (N = 30,204). Those properties that have not been booked (n = 13,541) are excluded from the analysis since their corresponding prices do not reflect equilibrium prices (i.e., inactive accommodations). For the retained sample (n = 16,663), we compute the average daily rate (ADR) as the mean price for booked days during August 2016. This variable will act as our dependent variable.

The dataset also provides detailed information about (1) the most relevant structural characteristics of the accommodations (type of property, entire versus shared/private room, minimum required stay, number of bedrooms), (2) reputation and quality indicators like the number of photos and the rating score from previous guests, which have been shown to explain Airbnb prices (Ert et al. 2016), (3) rental cancellation policies (Benítez-Aurioles 2018), and (4) some other host-specific variables like the experience gained as a host, whether the host holds the *Superhost* badge or the number of listings managed. The latter is considered as a proxy of market power (Casamatta et al. 2022).

Table 1 presents the definition of these variables together with summary statistics. The average daily rate is €257. There is great price dispersion in the dataset, ranging from a minimum of €11 to a maximum of €3558 per night. Figure A1 in Supplementary Material presents a histogram of the ADR, whose distribution is heavily right skewed.

**Table 1:** Definition and descriptive statistics of the property and host characteristics (N = 16,663)

Label	Description	Mean (%)	SD	Min	Max
ADR	Average daily rate	257.94	275.08	11	3558
Num. days booked	Number of days in August 2016 the listing was booked	16.49	9.43	1	31
Apartment	= 1 if apartment	45.21			
House	= 1 if house	34.43			
Villa	= 1 if villa	10.35			
Chalet	= 1 if chalet	2.37			
Other	= 1 if bed & breakfast, bungalow, castle, condominium, guesthouse, dorm, loft or townhouse, among others	7.64			
Entire	= 1 if entire property	83.34			
Shared/private	= 1 if the listing is shared with others/private room	16.66			
Min. Stay	Minimum number of nights required per booking	3.86	2.29	1	90
Bedrooms	Number of bedrooms	2.38	1.45	0	10
Num. Photos	Number of photographs available	22.71	15.70	1	780
Never rated	The listing has never been rated	27.69			
High rate	= 1 if 4,5 < score rating ≤ 5	41.10			
Medium rate	= 1 if 4 < score rating ≤ 4,5	19.79			
Low rate	= 1 if score rating ≤ 4	11.40			
Flexible Canc	Flexible cancellation policy	17.23			
Moderate Canc	Moderate cancellation policy	11.87			
Strict. Canc	Strict cancellation policy	69.92			
Instant Booking	Bookings are instantly accepted with no screening needed	25.92			
Superhost	= 1 if host attains the ‘Superhost’ badge	7.38			
Host Experience	Number of days since the account creation	444.47	408.17	5	2524
Num. listings	Number of listings owned by the host	35.58	118.58	1	624

About 83% of the sample is represented by entire properties. Most of the listings are (or located within) apartments (45%) or houses (34%), with an average of 2.4 bedrooms. The minimum stay demanded by the host is 3.86 nights on average, with each property having around 23 photos. Concerning reputation indicators, approximately 28% of the properties have no visible rating. This might happen because the listing has received less than 3 reviews or because it has never been rented before. For those with a positive number of reviews, more than 40% have received high ratings. This is in line with the existing literature on user-generated content showing that online reviews are left-skewed (Fradkin et al. 2021). Whereas 17% of the host adopt a flexible cancellation policy (no cancellation fees), the vast majority (70%) enforce a strict cancellation policy (no cancellation fees only during the first 48 h since the booking). The share of properties allowing for an immediate booking is only 26%*.* This low figure could imply a certain type of screening of guests’ profiles and is consistent with potential discrimination as documented in some studies (Edelman et al. 2017; Ahuja and Lyons 2019). Importantly, only 7% of hosts attain the *Superhost* status*.* This is a quality badge conceded by the platform to those hosts that satisfy several requirements and represent a relevant quality signal for potential guests.^5^

On average, hosts’ experience in the Airbnb platform is 444 days. However, the large standard deviation (SD = 401) indicates the market is composed of both highly experienced and unexperienced hosts. Interestingly, hosts manage on average 35 listings. This high mean value is the result of the process of professionalisation of Airbnb markets that makes it nowadays to be far from the original peer-to-peer sharing paradigm (Gil and Sequera 2020; Dogru et al. 2020). Indeed, only 36% of listings belong to single unit hosts.

Apart from the above-mentioned property and host characteristics, listings are georeferenced with longitude and latitude coordinates. Most of the listings are located in Mallorca (69%), followed by Ibiza (28%). The remaining 3% is evenly distributed in Formentera and Menorca.

#### Beach Characteristics

The Spanish *Ministerio para la Transición Ecológica y Cambio Demográfico* (MITECO) has made publicly available a cartographic tool that includes detailed geo-referenced information for all the beaches in the country.^6^ The dataset is updated annually and includes physical and environmental aspects, geographic extension data and facilities. We retrieved the corresponding dataset for the Balearic Islands in the year 2016. This contains information for a total of 263 beaches: 52% are located in Mallorca, 25% in Ibiza, and the remaining 23% in Menorca and Formentera. From the array of beach characteristics available, we select the following variables for the analysis:

- Length: beach extension (in kilometers)
- Width: width of the beach (in meters). This variable is the average of beach width during low tide and high tide.
- Sand type: dummy variables for the predominant type of sand: white (*Clear Sand*), gold (*Gold Sand*) or dark (*Dark Sand*).
- Type of coastal frontage: dummy variables capturing the type of environment behind the beach. There are five types of coastal frontage in the dataset: urban (*Urban front*), semi-urban (*Semiurban front*), cliff-type (*Cliff front*), mountain-type (*Mountain front*) and dune-type (*Dune front*).
- Vegetation: a dummy for the presence of vegetation in the beach (*Vegetation*).
- Protected area: a dummy indicator for whether the beach contains any protected space (*Protect. Area*), either in the form of *parque natural*, *paisaje protegido*, LIC (*Lugares de Importancia Comunitaria*) or ZEPA (*Zonas de Especial Protección para las Aves*).
- Tide: a dummy for predominant average calm tide (*Calm tide*) as opposed to heavy swell.
- Accessibility: dummy indicators for whether the beach is easily accessible on foot (*Easy Acc*), it has a difficult access (*Diff. Acc*) or it can only be accessed by boat (*Only by boat*).
- Degree of urbanization: this refers to the area in which the beach is located. Three types are distinguished depending on the number of buildings in the surroundings: isolated (*Isolated*), semi-urban (*Semi-Urban*) and urban (*Urban*).^7^

The definition of the beach characteristics presented before is based on objective environmental criteria set by experts at MITECO.^8^ The dataset offers other valuable information concerning the presence of different services (toilets, showers, public telephones, bins, cleaning services, tourist office, etc.), the tenure of a promenade or the availability of designed spaces in the beach for nudism, scuba diving, surf or children. However, preliminary analyses indicate all these variables are strongly correlated with the length and width of the beach. As such, their inclusion in the analysis will produce serious multicollinearity problems. The beach characteristics presented above and used for the analysis present by contrast low correlation levels so that their joint inclusion in a regression framework does not produce collinearity concerns (see Table A1 in Supplementary Material).^9^

Table 2 reports descriptive statistics of the beach variables introduced above. The average length is 360 m, with a mean width of 39 m. Nonetheless, there is notable variability in these two dimensions across beaches. Most beaches mainly have white sand (50%) or gold sand (43%). Concerning the type of coastal frontage, 25% and 32% of the beaches present an urban and semi-urban frontage, respectively. Around 16% have a cliff-type frontage while another 16% exhibits a mountain-type frontage. The remaining 8% has a dune-type frontage. Approximately 67% have coastal vegetation in the beach and 57% present calm tide. The share of beaches with protected areas inside them is 35%. The majority are easily accessible on foot (86%), although 2% can only be reached by boat and 9.5% have a difficult access. Finally, 38% are placed in isolated areas, 35% in semi-urban locations and 26% in urban zones.

**Table 2:** Definition and descriptive statistics for beach characteristics (N = 263)

Label	Definition	Mean (%)	SD	Min	Max
Length	Beach length (in kilometres)	0.36	0.685	0.01	4.60
Width	Beach width (in metres)	39.21	36.70	3	250
Clear Sand	= 1 if white sand	50.57			
Gold Sand	= 1 if golden sand	43.34			
Dark Sand	= 1 if dark sand	6.08			
Urban front	= 1 if urban frontage	25.85			
Semiurban front	= 1 if semi-urban frontage	32.69			
Cliff front	= 1 if cliffside beach	15.96			
Mountain front	= 1 if mountain-type frontage	16.73			
Dune front	= 1 if dune-type frontage	8.74			
Calm tide	= 1 if calm tide	57.03			
Vegetation	= 1 if coastal vegetation	67.30			
Protect. Area	= 1 if contains any protected space	35.36			
Easy Acc	= 1 if easily accessible on foot	86.69			
Diff. Acc	= 1 if difficult access on foot	9.50			
Only by boat	= 1 if only accessible boat	2.28			
Isolated	= 1 if isolated	38.02			
Semi-Urban	= 1 if semi-urban area	35.36			
Urban	= 1 if urban area	26.61			

To study the role of the above-presented beach characteristics on Airbnb property prices, we need a vis-à-vis matching between properties and beaches. Since each property is georeferenced with latitude and longitude coordinates, this was done by computing the Euclidean distance between each property and the shoreline of each beach. Subsequently, each property was only matched with the closest beach. As a result, each of the 16,663 listings in the dataset were linked to one of the 263 beaches in the islands. The average distance to the shoreline is 3.92 km. Due to its greater size, properties in Mallorca islands are on average far more distant (4.92 km on average), than in the other islands (1.95, 1.68 and 1.66 km for the case of Menorca, Ibiza and Formentera, respectively). Nevertheless, about 17% of properties are located within 500 m from the shoreline while about 33% lie within 1 km.

**Fig. 1:**
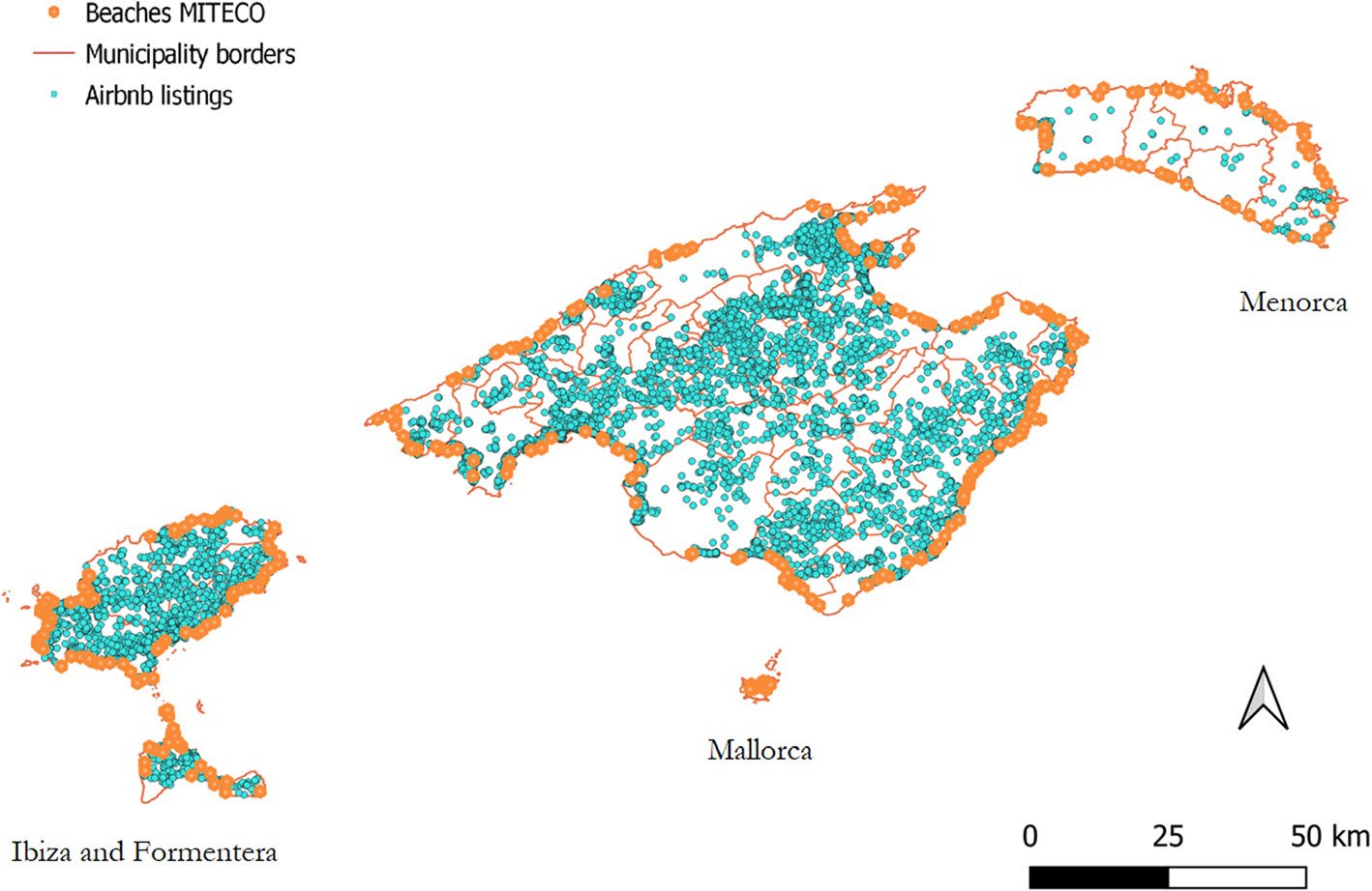
Geographical distribution of Airbnb listings and MITECO beaches across the Balearic Islands

**Fig. 2:**
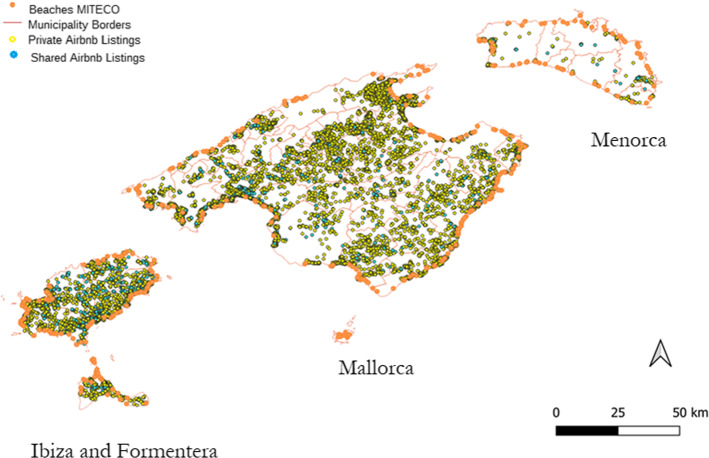
Geographical distribution of Airbnb listings and MITECO beaches across the Balearic Islands, by property type

Figure 1 plots the location of the listings and the beaches in the four islands. Light-blue points represent Airbnb listings, orange points represent beaches while pink lines delimit municipality borders.^10^ As can be seen, most of the listings are in Mallorca (69%), followed by Ibiza (28%), Formentera (1.5%) and Menorca (1.5%). However, Ibiza is the island with highest concentration (8.16 listings per km^2^), followed by Mallorca (3.15 listings per km^2^), Formentera (3.00 listings per km^2^) and Menorca (0.36 listings per km^2^). In Fig. 2, we distinguish between entire properties (yellow dots) and shared properties (light blue dots). Ibiza has the highest proportion of shared properties (25.6%), followed by Menorca (19.4%), Formentera (16.2%) and Mallorca (13.1%). Maps with average prices and number of properties per municipality are presented in Figures A3 and A4 in Supplementary Material.

## Econometric Modelling

Consistent with the theoretical framework presented in Sect. 3, the baseline empirical model to be estimated is the following:

7 $$Ln ADR_{i} = \alpha + \beta Beach Atrib_{i} + \theta \ln Distance_{i} + \gamma C_{i} + \delta H_{i} + Mun FE_{i} + \varepsilon_{i}$$

where $Ln ADR_{i}$ is the (log of) average daily rate, $Beach Atrib_{i}$ gathers the beach characteristics of interest, $\ln Distance_{i}$ measures the Euclidean distance between each listing and the shoreline (in logs), $C_{i}$ reflects listing structural characteristics, $H_{i}$ refers to host features, $Mun FE_{i}$ are municipality fixed effects and $\varepsilon_{i}$ is a normally distributed error term.

One unresolved issue when estimating hedonic price models is the appropriate functional form (see on this Cropper et al. 1988). Whereas some use linear specifications, the semi-log specification is by far the most widely used (Gibbons et al. 2014). In the hospitality accommodation context, Faye (2021) advocates for formally testing the appropriate functional form through a Box-Cox regression. Auxiliary Box-Cox regressions (Table A2 in Supplementary Material) provide support for the proposed functional form of the hedonic price function. Log transforming the dependent variable also helps it to resemble the normal distribution (Figure A2 in Supplementary Material).

The inclusion of municipality fixed effects intends to capture any omitted factor at the municipality level that impacts prices, like accessibility to transportation hubs, provision of public services or the sociodemographic composition of the area (Rigall-i-Torrent et al. 2011; Saló et al. 2014). Omitted municipality confounders are a common concern in related works (Leggett and Bockstael 2000; Landry et al. 2021). As shown by Kuminoff et al. (2010), adding spatial fixed effects substantially reduces the bias from omitted variables in cross-sectional data. Therefore, these fixed effects capture price shifts across submarkets, gathering the effect of all public amenities $Z_{i}$ other than beach characteristics.^11^

As shown in Figs. 1 and 2, listings are sparsely distributed throughout the islands. This results in some listings being close to the shoreline while others locating far away. Even though we control for it in the regression, the model in (7) assumes an equal impact of beach characteristics on daily rates for all the sample, regardless of listings’ proximity to the coast. Consistent with related studies (Landry and Hindsley 2011; Saló et al. 2014; Rigall-i-Torrent et al. 2011; Landry et al. 2021), we expect the capitalization effect of beach characteristics to decrease as distance to the shoreline increases. To capture this *distance-decay* effect, we expand the specification in (7) with interaction terms between the log of distance and beach amenities as follows:

8 $$Ln ADR_{i} = \alpha + \beta Beach Atrib_{i} + \theta \ln Distance_{i} + \tau Beach Atrib_{i} \times \ln Distance_{i} + \gamma C_{i} + \delta H_{i} + Mun FE_{i} + \varepsilon_{i}$$

The expanded specification with interactions allows us to test for edge and proximity effects in the sense of Walsh et al. (2011); that is, the exposure to a specific environmental amenity is moderated by the distance to the shoreline in a non-linear way through the log transformation.

Some aspects concerning our empirical strategy deserve mention. First, unlike other related studies (Landry and Hindsley 2011; Walsch et al. 2017; Catma 2020), we do not restrict the sample to those units that fall within a certain distance threshold, at least in the main analysis. We, instead, assume a continuous distance decay effect. This assumes that capitalization effects expand beyond the immediate vicinity. To inspect potential spatial discontinuities, prior to the analysis we conducted *binscatter* regression (Cattaneo et al. 2021).^12^ We do not detect any clear discontinuity, so we opted for considering the whole sample in the main analysis. A hedonic semiparametric regression controlling for structural characteristics and host attributes also indicates the price gradient with respect to distance from the shoreline is linearly decreasing (available upon request). Nonetheless, spatial discontinuities are examined in more detail later in the robustness checks section.

Second, we work with a cross-sectional database for the summer peak period rather than longitudinal data for two main reasons. On the one hand, it is widely known that panel datasets in a hedonic framework allows the research to control for unobserved quality in the form of fixed effects and lead to unbiased estimates. However, in our case study, the beach amenities are time invariant so their implicit values cannot be separately identified from property fixed effects.^13^ On the other hand, vacation rental markets exhibit high seasonality (particularly coastal ones) so that consumers change the mix of hedonic characteristics selected at different periods (Smith and Palmquist 1994), which produces shifts in the price function. As such, using panel data in this specific context would lead to implicit prices that conflate consumers’ WTP for the coastal amenity with intertemporal substitution effects. In this regard, some authors warn about pooling data for different periods since any temporal change in preferences, income or unobserved amenities changes the shape of the price function (Kuminoff et al. 2010; Banzhaf 2021). Moreover, in the presence of time-varying omitted variables panel data estimates do not produce more accurate estimates for implicit prices than cross-sectional regressions. Additionally, there is not much price dispersion within the month (Supplementary Material, Figure A9), thereby making the average daily rate an accurate indicator of prices. Therefore, we prefer to estimate the hedonic price function at a given point in time (August 2016).^14^

Third, a key aspect for the parameter identification of beach characteristics and distance to the shoreline conditional on the municipality fixed effects is the existence of sufficient variability in the number of beaches (and the associated distance to them) within municipalities. Table A3 in Supplementary Material presents the number of beaches and properties per municipality and the mean distance to the shoreline of all the listings located in each municipality. As shown there, properties are matched to several beaches within municipalities so that beach amenities and municipality fixed effects are separately identified.^15^

**Table 3:** WOLS hedonic price regression estimates under different model specifications

Dependent variable: Ln ADR	(1)	(2)
Explanatory variables	Coeff. (SE)	Coeff. (SE)
Ln distance	0.001	0.029
	(0.007)	(0.039)
Ln length	0.027***	0.028***
	(0.010)	(0.009)
Ln length × Ln distance		− 0.007
		(0.006)
Ln width	− 0.016	− 0.008
	(0.013)	(0.013)
Ln width × Ln distance		− 0.015
		(0.009)
Gold sand	0.014	0.010
	(0.025)	(0.025)
Gold sand × Ln distance		0.019
		(0.017)
Dark sand	− 0.040	− 0.052
	(0.043)	(0.038)
Dark sand × Ln distance		0.021
		(0.023)
Cliff front	0.094***	0.092***
	(0.033)	(0.034)
Cliff front. × Ln distance		− 0.014
		(0.027)
Semi-urban front	0.116***	0.119***
	(0.038)	(0.036)
Semi-urban front. × Ln distance		0.008
		(0.017)
Mountain front	0.090*	0.129***
	(0.046)	(0.046)
Mountain front. × Ln distance		− 0.059*
		(0.030)
Dune front	0.112**	0.103**
	(0.044)	(0.046)
Dune front. × Ln distance		0.017
		(0.031)
Calm tide	− 0.022	− 0.019
	(0.022)	(0.023)
Calm tide × Ln distance		− 0.006
		(0.013)
Vegetation	0.051**	0.045**
	(0.022)	(0.020)
Vegetation × Ln distance		0.026*
		(0.014)
Protec. area	− 0.007	0.030
	(0.026)	(0.027)
Protect. area × Ln distance		− 0.037**
		(0.017)
Diff. access	0.113***	0.206***
	(0.043)	(0.041)
Diff. access × Ln distance		− 0.106***
		(0.027)
Only by boat	0.094*	0.054
	(0.055)	(0.057)
Only by boat × Ln distance		0.020
		(0.043)
Isolated envir	− 0.069*	− 0.100***
	(0.039)	(0.037)
Isolated envir. × Ln distance		0.026
		(0.026)
Semi-urban envir	− 0.075**	− 0.078**
	(0.033)	(0.031)
Semi-urban envir. × Ln Distance		0.019
		(0.018)
Structural characteristics	YES	YES
Host characteristics	YES	YES
Municipality fixed effects	YES	YES
Constant	3.592***	3.569***
	(0.073)	(0.073)
VIF	4.09	5.69
Observations	16,663	16,663
R-squared	0.746	0.747

Clustered standard errors at the beach level in parentheses. ***p < 0.01, **p < 0.05, *p < 0.1

The reference categories are *Clear sand*, *Urban front*, *Easy Acc* and *Urban envir*

Notwithstanding this, the identification of the hedonic price function using cross-sectional data relies on some important assumptions and has some limitations (Gibbons et al. 2014; Landry et al. 2021). Conditional on the large set of controls for intrinsic attributes, host characteristics and municipality fixed effects, we assume independence between unobserved listing attributes and beach amenities. Fourth, standard errors are clustered at the beach level to correct for potential Moulton bias (Moulton 1990) when specifying aggregate level variables. This is because each Airbnb that is assigned to a specific beach shares a common component of the variance that is not entirely attributable either to their private attributes or to the rest of controls. If not accounted for, this produces the error terms of listings close to the same beach to be positively correlated, leading to a downward bias in the standard errors (see on this Abadie et al. 2017). The clustering adjustment also alleviates potential omitted variable bias from unmeasured beach characteristics.

Finally, to reflect market equilibrium prices, our dataset is restricted to those properties that have been rented at least one night during August 2016. However, as presented in Table 2, there is nonnegligible variation in the number of days each of the retained listings has been occupied.^16^ In line with a large literature on sales-weighted hedonic price indexes (Reis and Santos Silva 2006; Silver 1999), observations are weighted by the number of days the property has been booked during the month (*Num. days booked*). In this way, the estimation of capitalization effects in a quantity-weighted hedonic regression will diminish the influence of unrepresentative prices (Diewert 2003).^17^ Consequently, Eqs. (7) and (8) are estimated by Weighted Least Squares (WLS).

## Results

### Main Analysis

**Fig. 3:**
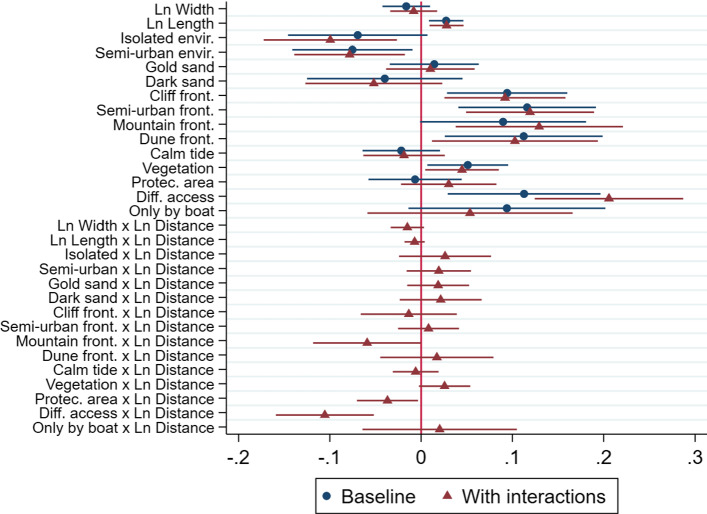
Coefficient estimates and confidence intervals (Table 3) for the beach characteristics and interactions with the log of distance

Table 3 presents the results for the hedonic regressions. Model 1 reports the estimates from the specification in (7) with no interaction effects; Model 2 shows the results from the full specification in (8). We only report the coefficients for the coastal amenities to save space, but the parameter estimates for the rest of controls are presented in Supplementary Material, Table A4. A plot of the coefficient estimates and confidence intervals is presented in Fig. 3.

The distance to the shoreline is not significant for explaining the ADR (neither the partial derivative, see column 2). A regression including proximity dummy variables to allow for non-linearities also produces insignificant coefficients (Supplementary Material, Table A4). This is contrary to our expectations, since one would expect daily rates to decrease as we move away from the beach. As shown in Figure A8, Panel D in Supplementary Material, this result is due to the inclusion of municipality fixed effects, which already capture common level differences associated with closeness to the beach.^18^

Beach length is positively associated with listings’ daily rates. This is consistent with previous studies showing that tourists attach value to longer coastlines (Hamilton 2007; Onofri and Nunes 2013). Surprisingly, beach width is not found to exert significant effects on prices.^19^ This is contrary to prior works focusing on housing prices (Catma 2020; Gopalakrishnan et al. 2016b; Landry and Hindsley 2011; Landry et al. 2021). Nonetheless, the positive effects of beach width documented in related studies are typically detected for properties in close proximity to the beach. For instance, Landry and Hindsley (2011) indicate beach width exerts a negative effect on prices in regressions that consider properties threshold points of up to 500 or 600 m from the shoreline. Moreover, Rigall-i-Torrent et al. (2011) document that beach width is negatively associated with hotel prices in Costa Brava, possibly through a crowding mechanism. Moreover, whereas beach width offers recreational and protection value against storm surge in the sales market, it only has recreational value in the rental market, which might partially explain its non-significance. Additionally, no price differences are detected based on the sand colour.

Concerning the type of beach frontage, listings located close to beaches with semi-urban, cliff-type and mountain-type frontages, respectively (relative to an urban frontage) are highly priced. Plausibly, this finding is explained by aesthetic motives based on subjective evaluations. In this regard, people have been shown to attach value to open green spaces and scenic amenities (Gibbons et al. 2014; Athukorala et al. 2019). Aesthetics and visual quality have been also revealed as key factors driving tourism demand (Onofri and Nunes 2013) and residential properties (Lansford and Jones 1995). For instance, dunes have been found to capitalized into property values (Landry and Hindley 2011). Furthermore, Airbnb guests seem to value more beaches in highly urbanized areas. This likely reflects the fact that beaches with a large number of buildings in its surroundings might convey greater accessibility to ancillary facilities like shops, restaurants or bars and public services like transportation hubs (Rigall-i-Torrent and Fluvià 2011; Saló et al. 2014).

Interestingly, ADRs do not vary depending on whether the beach exhibits an average calm tide. Similarly, the presence of protected natural spaces does not convey any price premium either. However, vegetation in the beach is associated with higher ADRs. This suggests the green spaces are key attributes for coastal quality, plausibly through their aesthetic value. Regarding the role of accessibility, properties with a difficult access on foot exhibit higher prices relative to comparable accommodations with an easy access (reference category). This finding falls in line with Rigall-i-Torrent and Fluvià (2011) and might be interpreted in terms of strong preferences for exclusivity. Beaches with a difficult access might be less crowded, thereby offering users more privacy and space for recreation. In this case, the interaction term with the log of distance is negative and significant, implying that the price premium of exclusivity decreases as we move away from the shoreline.

Overall, we find little evidence for distance decay capitalization when considering properties located in the vicinity of the shoreline and properties in inland areas. Most of the interaction terms are not significant. Although this could be partially due to the inclusion of municipality fixed effects in the regression and the clustered standard errors, we believe this might also reflect that capitalization effects in tourism markets operate differently from the housing market, being potentially wider and less concentrated around the shoreline.

Concerning the effect of the rest of control variables, the estimates are consistent with Airbnb hedonic price studies. Entire properties are more expensive, with daily rates being positively correlated with the number of bedrooms and the minimum stay (Ert et al. 2016; Gibbs et al. 2018). Chalets and villas convey significant price premiums. Properties with strict cancellation policies are more expensive (Faye 2021; Moreno-Izquierdo et al. 2020), whereas enabling the instant booking option is associated with lower prices (Gibbs et al. 2018; Casamatta et al. 2022). Daily rates increase with host experience and the number of photos (Moreno-Izquierdo et al. 2020; Casamatta et al. 2022). However, holding the *Superhost* badge is not found to be significant. Finally, rates positively increase with the number of listings the host has on property, as found in Faye (2021), Moreno-Izquierdo et al. (2020) and Gibbs et al. (2018). This result is consistent with our theoretical arguments about the potential exercise of market power: multi-property host are more likely to better assess the price elasticity of demand and therefore to exercise market power, particularly during the peak season (Casamatta et al. 2022).

### Price Premiums

The price premiums associated to each beach characteristic, *ceteris paribus*, are obtained by partially differentiating the hedonic price function. Table 4 presents the average marginal effects (AME) for each beach amenity (i.e., $\mathop \sum \limits_{i = 1}^{N} \frac{1}{n}\frac{\partial \ln ADR}{{\partial X}}$), the corresponding price premiums in percentage terms and in euros.^20^ For the non-significant variables, price premiums are taken as zero.

**Table 4:** Average marginal effects and price premiums of beach characteristics

Variable	AME	Price premium (%)	Price premium (€)
Ln length	0.024***	2.42	6.24
Ln width	− 0.015		
Gold sand	0.019		
Dark sand	− 0.040		
Cliff. front	0.084***	8.76	22.60
Semi-urban front	0.123***	13.08	33.74
Mountain front	0.098**	10.29	26.54
Dune front	0.111***	11.73	30.26
Calm tide	− 0.021		
Vegetation	0.058***	5.97	15.40
Protec. area	0.011		
Diff. access	0.151***	16.20	41.79
Only by boat	0.064		
Isolated envir	− 0.086**	− 8.24	− 21.25
Semi-urban envir	− 0.068**	− 6.57	− 16.95

***p < 0.01, **p < 0.05, *p < 0.1

The elasticity of ADRs with respect to beach length is 2.4, which implies that, on average, tourists are willing to pay €6.24 more for a one percent increase in the beach length. Compared to beaches with an urban frontage, beaches with cliff-type, semi-urban type, mountain-type and dune-type frontage register price premiums between 8 and 13%. This implies tourists are willing to pay between €22–€30 to locate in beaches with non-urban frontages. The presence of vegetation is associated with a price premium of around 6%, which corresponds to €15.4. Properties close to beaches with difficult access exhibit a 16.2% price premium, which represents around €42 more relative to easily accessible beaches. Finally, properties in semi-urban areas are less valued, with tourists’ willingness to pay being €17 lower than for properties in urbanized areas. The disamenity value is slightly larger for isolated environments, for whom tourists are willing to pay €21 less per day.

### Distance Thresholds

As mentioned before, the related literature has documented that beach capitalization effects diminish with distance to the shoreline. However, conditional on the municipality fixed effects, our regressions do not detect distance decay effects except for the difficult access dummy. To inspect whether our findings could be affected by the spatial extent from the shoreline considered, we repeated the estimation considering different subsamples.

First, to capture potential capitalization effects associated with beachfront view, we restricted the sample to listings in close proximity to the beach falling into distance thresholds of 100, 200, and 300 m (Supplementary Material, Table A5). Very few variables are found to be significant, which is likely to be due to the small sample sizes and potential identification problems associated with the reduced variability in properties’ characteristics close to the beach (Kuminoff et al. 2010). Indeed, descriptive statistics point to some sorting in structural characteristics over space (Supplementary Material, Table A6). This also holds when we repeat the regressions without controls and with non-clustered standard errors (available upon request).

Next, we considered subsamples of listings that are located up to 500, 750, 1000, 2000, 3000, 4000 and 5000 m away from the shoreline (Supplementary Material, Table A7). Overall, the results of these regressions are in line with those presented in Table 3. Beach length is positively and significantly associated with ADR, but the magnitude of the effect decreases as we move to subsamples that consider listings located more distant from the beach. Non-urban frontage types are associated with higher prices, with their capitalization effects decreasing as inland listings are added to the sample. Difficult accessibility is consistently found to translate into price premiums, especially when considering subsamples based on small distance thresholds to the shoreline.

Finally, to deal with the above-mentioned potential sorting-in-characteristics issue, we adopted the methodology proposed by Davis (2011). First, we defined several distance thresholds dummies (less than 500, 1000, 1500, 2000 and 3000 m) and computed the propensity scores, obtained as the conditional probabilities from probit regressions of the distance dummies on the structural characteristics of the properties. Second, we re-estimated the model using the whole sample but weighting observations by the propensity scores from the first step (Supplementary Material, Table A8). By doing so, we aim at balancing the mean characteristics of properties over space. The results are very consistent across distance thresholds and very similar to the ones presented in Table 3 (Supplementary Material, Tables A9–A10). Therefore, once we balance properties’ structural characteristics, the capitalization effects of amenity values are robust across different distance thresholds.

### Robustness Checks and Extensions

We performed a battery of robustness checks and extensions to our main analysis. First, we conducted a stepwise estimation in which the blocks of explanatory variables were sequentially included in the regression (Supplementary Material, Table A11). Results prove the importance of controlling for listing structural characteristics and host variables to get finer estimates. The inclusion of municipality fixed effects appears to be particularly relevant as it produces notable changes in magnitude and significance in the estimates. As discussed in Kuminoff et al. (2010), spatial fixed effects are an effective strategy for addressing spatially correlated omitted variables. Second, we re-estimated the model considering different standard error clustering structures. Specifically, we first clustered standard errors at the host, postal code and municipality level, separately (Supplementary Material, Table A12). Second, we also run the regressions using two-way standard error clustering following Cameron et al. (2011) (Supplementary Material, Table A13). Furthermore, we implemented the arbitrary cluster correlation proposal originally developed by Conley (1999) and recently reformulated by Colella et al. (2019) considering different distance thresholds (Supplementary Material, Table A14). Consistent with the econometric literature on the topic (e.g., Abadie et al. 2017), these results highlight the relevance of allowing for cross-sectional dependence in the residuals, as early illustrated in Moulton (1990). Nonetheless, the statistical significance of the variables remains barely unchanged across the different clustering structures as compared to Table 3.

Third, we re-estimated the model using listings’ closeness to the beach (inverse of distance) rather than Euclidean distance (Leggett and Bockstael 2000; Landry et al. 2021). Results are presented in Supplementary Material (Table A15) and are about the same as in the main analysis. Fourth, we conducted separate regressions by type of property, distinguishing between entire and shared/private bedroom options (Supplementary Material, Tables A16 and A17 and Figure A12). Although the point estimates slightly differ across types of properties, Wald tests do not reject the null hypothesis of coefficient equality for most of the attributes. Similarly, we run separate regressions by island, since they could be considered as separate markets (Supplementary Material, Tables A18 and A19). A comparison of the coefficients is presented in Figure A13 in Supplementary Material. Interestingly, as opposed to the pooled analysis, we find no significant price differences based on beach length, the type of beach frontage and the urban environment for Mallorca. This holds even when municipality fixed effects are excluded. In contrast, Ibiza and Formentera exhibit significant premiums for these characteristics. These results hold when we restrict the sample to properties within 1000 m from the beach. This suggests there is relevant heterogeneity in the value of coastal amenities across areas, plausibly related to unobserved quality. Differences in the capitalization effects of environmental amenities across geographical areas are also presented in Gibbons et al. (2014).

Fifth, authors like Gopalakrishnan et al. (2016b) and Landry et al. (2021) warn about potential measurement error in beach width that can produce attenuation bias in the coefficient estimates. To explore this, we perform IV regression using binary indicators for the presence of a tourism office, a telephone cabin, access for disabled people and a yacht club in the beach as instruments. We assume these variables are strongly correlated with beach width but uncorrelated with Airbnb prices, conditional on the rest of beach attributes and controls. Table A20 in Supplementary Material presents the corresponding first stage and 2SLS estimates. The F from first stage (F = 440) and individual t-test statistics indicate the instruments are strongly correlated with (the log of) beach width. An auxiliary placebo regression indicates these variables satisfy the exclusion restrictions, since they are not significantly correlated with ln ADR conditional on the remaining controls. Sargan test does not reject the null hypothesis that the overidentifying restrictions are valid (*p*-value = 0.141). Durbin Wu-Hausman test does not reject the null hypotheses of exogeneity (*p*-value = 0.932). Similar to Landry et al. (2021), the 2SLS point estimate for ln Width is very close to that obtained from OLS (column 1 in Table 3). All in all, we conclude there is no evidence of endogeneity in beach width due to potential measurement error.

Sixth, our main analysis uses average daily prices over the days the property was booked. Using the raw daily data, we re-estimated the model using a Pooled OLS and a panel random effects regressions (at the property level) on the daily frequency including a full set of daily fixed effects to absorb all time variation in prices. The estimation results are presented in Table A21 and remain similar to the cross-sectional analysis in Table 3. Interestingly, the Pooled OLS and the RE regression on a daily basis produce very close estimates. Figure A14 plots the daily fixed effects obtained from the RE regression. Compared to August 1, prices remain largely unchanged during the first half. In contrast, prices decrease by the end of the month, plausibly through a lower demand mechanism.

Seventh, we re-estimated the model considering only properties that were occupied for at least 15 days during the month of August (Supplementary Material, Table A22). In this way, we examine capitalization effects among the highly demanded segment. The results remain largely unchanged. Moreover, to inspect the sensitivity of our findings to the weighting scheme, we re-estimated the model without weights (Supplementary Material, Table A23). The coefficients are pretty similar.

Eighth, we re-estimated the model using postal code fixed effects (instead of municipality fixed effects) (Supplementary Material, Table A24). Beach length and the type of sand indicators lose their significance, potentially through the high overlapping between beaches and postal codes. That is why we prefer to use municipality fixed effects in the main analysis to control for unmeasured geographical aspects.

Finally, we re-estimated the model in (9) replacing the municipality fixed effects by the following municipality characteristics: population (*Pop*), average age (*Av. Age*), percentage of foreign residents *(% Foreign*), percentage of low educated residents *(% Low educ*), average household size (*Av. House size*), percentage of large dwellings *(% Large dwellings*), gross income (*Gross Income*), Gini inequality index (*Gini*), number of Airbnb properties (*Airbnb listings*) and number of hotel beds (*Hotel beds*). A short description of the variables, data sources, descriptive statistics and their construction is presented in the Supplementary Material, Table A25. The estimation results are shown in Table A26 in Supplementary Material. We find Airbnb daily rates increase with the number of competitors and the Gini index but decrease with the average age of the population in the neighbourhood. The rest of variables are not significant. In any case, a comparison with the results in Table 3 shows there are notable differences in the point estimates between the two model specifications. This indicates that including the full set of municipality fixed effects better captures all geographic-level confounders. Indeed, scatter plots of the estimated municipality fixed effects over the municipality controls detect some relevant correlations (Supplementary Material, Figures A15–A26).

## Conclusions

The current study has investigated how peer-to-peer tourist accommodations capitalize the environmental and recreational value of several beach amenities. Although peer-to-peer accommodations are associated with some negative externalities, on the positive side they stimulate local economies, represent additional income for residents, satisfy the needs of new consumer segments and provide potential additional revenues through tourist tax rates. We have used data for 16,663 Airbnb listings in the Balearic Islands that were booked at least one night during August 2016. Using hedonic price modelling, we have estimated the capitalization effects of several coastal attributes like beach length and width, sand type, presence of vegetation, coastal frontage or accessibility. Our model specification has explicitly allowed for the potential moderating effect of distance documented in previous studies through interaction terms.

According to our results, tourists are willing to pay around €6.24 more per day for a percentage increase in beach length, everything else being equal. Tourists are found to attach value to the presence of vegetation (+ €15.4) and prefer beaches with a difficult access over easily accessible ones (+ €41.8), which is interpreted in terms of demand for exclusivity and lower occupancy. Interestingly, we find notable price premiums depending on the coastal frontage. In particular, tourists are willing to pay €30.2 and €26.5 per night if the beach has a dune-type of mountain-type frontage over an urban one, *ceteris paribus*. Concerning the degree of urbanization, Airbnb guests prefer urbanized areas (− €17 and − €21.2 per day in the case of beaches in semi-urban or isolated environments, respectively). This likely captures preferences for amenities that correlate with the number of buildings in the surroundings of the beach. On the contrary, no significant capitalization effects are detected for the type of sand, calm tide, beach width or the presence of protected natural spaces. Furthermore, we do not detect prices to decrease as distance to the shoreline increases, conditional on municipality fixed effects. Overall, the hedonic estimates suggest Airbnb users attach economic value for non-marketed aspects like beach exclusivity, the presence of aesthetic natural environments and beach length.

The work has relevant implications for coastal management policies and tourism planning. As discussed in several works (Ghermandi 2015; Roig-Munar et al. 2019), the Balearic Islands stand as a region with high recreation values but with poor protection status and high erosion risk. Beaches represent ecological habitat, aesthetic amenities and serve as a natural protective barrier against storm surge. Beyond that, they represent major attraction factors for tourism activities that need to be preserved. Coastal management policies implemented during the last 50 years has been mostly devoted to ensuring leisure activities rather than rehabilitating and protecting the shoreline, leading to serious ecological threats. In a recent report commissioned by the local Economic and Social Council, shoreline retreat was identified as one of the main dangers that climate change is imposing on the islands, requiring urgent mitigation and adaptation policies by 2030 (De Vílchez et al. 2019). Similar concerns are presented in Enríquez et al. (2017) and Torres et al. (2021). Experts warn that the current uncontrolled *touristification* of environmental resources is likely to accelerate erosion and the loss of beach width, which will paradoxically result in a loss of tourism attractiveness for the islands.

Uncovering the economic value that tourists attach to coastal amenities offers relevant information for local authorities when developing benefit–cost analyses for potential beach nourishment projects. In areas with strong dependence on the tourism sector, empirical estimates of the value of beaches for tourism activities can therefore play an important role in the design and implementation of conservation policies to adapt to sea-level rise. Additionally, the estimates of capitalization effects could also be valuable for the development of green infrastructure investments aimed at protecting beach vegetation and natural species, which we found to be economically valued by visitors.

Mitigation policies are particularly relevant for the sustainable recovery of the tourism industry after COVID-19. The need for social distancing has shifted tourists’ preferences towards the practice of outdoor activities. The expected rebound in tourism flows when the pandemic is over requires policy responses to avoid massification and overtourism that could further damage coastal ecosystems. As discussed in Enríquez and Bujosa (2020), tourists visiting the Balearic Islands exhibit a positive attitude towards beach protection and get disutility for beach retreat and beach closure. Maintaining high levels of beach quality therefore adds significant value to the tourism experience and likely enhances repeat visit behaviour. Marketing campaigns should not only promote the islands’ environmental resources as an attraction factor but also encourage responsible consumption and sustainable recreational practices among visitors.

Our analysis has some limitations that we consider as avenues for future research. First, we cannot completely rule out potential biases stemming from omitted variables. This is a common risk in related studies using cross-sectional data. As discussed before, because of the reduced time variability in beach characteristics, the use of longitudinal data makes little sense in the context. In any case, since we control for a wide array of observable characteristics, we consider omitted variable biases are minimized. Second, we do not consider potential spatial dependences in price formation. Although this is partially controlled for through the municipality fixed effects and the standard error clustering structure at the beach level, future studies could expand our analysis using spatial econometrics methods. Finally, for the reasons discussed earlier in the paper, we have used data for the high season (August 2016). Future research should deepen into potential seasonal differences in the capitalization of environmental amenities between low and high seasons.

## Supplementary Information

Below is the link to the electronic supplementary material.

Supplementary file1 (DOCX 2318 KB)

## Footnotes

1 The peer-to-peer economy (also known as collaborative consumption or sharing economy) has disrupted traditional business practices in the accommodation sector (Guttentag 2015). It consists of the use of under-utilized inventory through fee-based sharing that has been framed as a more sustainable form of consumption that could foster innovation (Martin 2016).

2 An important advantage of using these data is that nourishment projects have not been undertaken in the beaches belonging to the Balearic Islands, thereby avoiding problems of reverse causality in beach width (Gopalakrishnan et al. 2016b; Landry et al. 2021).

3 This characterization is static in nature. Rosen’s framework defines the hedonic price function as a time-specific equilibrium between supply and demand. As discussed in Banzhaf (2021) and Kuminoff et al. (2010), any temporal shock to preferences, income or amenities can change the shape of the hedonic price function over time.

4 As in Feenstra (1995) and Cotteleer et al. (2008), we assume host characteristics (as a proxy of market power) do not interact with the private or public characteristics.

5 To become a *Superhost*, the host needs to meet the following criteria: (i) completed a minimum of 10 stays that sum up to 100 nights; (ii) maintained a response rate of 90% or higher; (iii) maintained a cancellation rate of 1% or less; and (v) maintained a general rate of 4.8/5 in the last 365 days (Airbnb 2021).

6 The latest version of the dataset can be downloaded at https://www.miteco.gob.es/es/cartografia-y-sig/ide/descargas/costas-medio-marino/guia-playas-descargas.aspx

7 Although some related studies have paid attention to the economic value of water quality in the real estate market (Leggett and Bockstael 2000; Walsh et al. 2011, 2017), this is not considered. On the one hand, all the beaches in the islands are highly homogeneous in this dimension at the period of analysis, exhibiting high levels of water quality (Consejería de Salud y Consumo 2016). Therefore, there is not enough water quality variability for identification (Leggett and Bockstael 2000). On the other hand, unlike it happens with other beach amenities, the level of water quality is likely to be an unobserved attribute to potential tourists.

8 While a single annual snapshot of beach characteristics is not ideal, it is the best available information and the common way to proceed in related studies (Landry et al. 2021).

9 The only exceptions are the categories *Urban* (degree of urbanization) and *Urban front.* (type of coastal frontage), whose correlation amounts to 0.72. For this reason, they are both left as the reference category in each case.

10 The municipality raster was downloaded from the website of the Spanish *Centro de Descargas*. https://centrodedescargas.cnig.es/CentroDescargas/linkUnMD. Accessed on December 10th 2021.

11 Postal code fixed effects are used as a robustness check. Figures A5-A6 in Supplementary Material plot the municipalities and postal codes of the islands.

12 This consists of first computing the residuals from auxiliary regressions of ln ADR and Ln Distance on the control variables and then *binscatter* the means within 20 equal-sized bins. See Figure A8 in Supplementary Material.

13 If the individual effects are treated as ‘random’, that imposes the same restrictive assumption that unobserved quality is uncorrelated with the structural characteristics. Mundlak correlated random effects modelling requires the regressors to be time variant.

14 Another reason for the use of panel datasets in hedonic studies using residential property values is that when buying a house consumers consider the future levels of local amenities (they are forward looking), thereby potentially requiring dynamic models for appropriate inference (Bishop and Murphy 2019). However, the static cross-sectional version of the hedonic price function seems to be appropriate in our setting because consumers demand Airbnb listings for reduced stays, so that they do not care about prospects in the future evolution of environmental amenities.

15 The reader might notice that the sum of beaches in each municipality in Table A3 is over 263 (the total number of beaches in the sample). This is because depending on their geographic location, properties within municipalities are matched with the closest beach, which in some cases could be a beach that is physically located in another municipality.

16 Figure A10 in Supplementary Material presents a clear negative relationship between the number of days booked and the ADR as predicted by microeconomic theory.

17 Weights are also recommended when working with averaged data (Machado and Santos Silva 2006).

18 A scatterplot between the estimates of the municipality fixed effects and the mean distance to the beach of all properties within the same municipality (Figure A11 in Supplementary Material) indicates the expected distance decay pattern is captured by the municipality fixed effects.

19 Potential attenuation bias from measurement error is inspected in robustness checks.

20 The price premiums for the dummy variables are calculated as PP = (exp(AME)-1)*100. The WTP is computed by multiplying the price premium by the average daily rate in the sample (€258).
