# Supplementary material for: The Economic Value of Coastal Amenities: Evidence from Beach Capitalization Effects in Peer-to-Peer Markets
Source: Environ Resour Econ (Dordr). 2022 Oct 1;84(2):529–57. doi: 10.1007/s10640-022-00735-5 (PMC9526391; doi:10.1007/s10640-022-00735-5)
Supplement: Supplementary file 1 — Supplementary file1 (DOCX 2318 KB) [file 10640_2022_735_MOESM1_ESM.docx]

**SUPPLEMENTARY MATERIAL FOR**

**The economic value of coastal amenities: Evidence from beach capitalization effects in peer-to-peer markets**


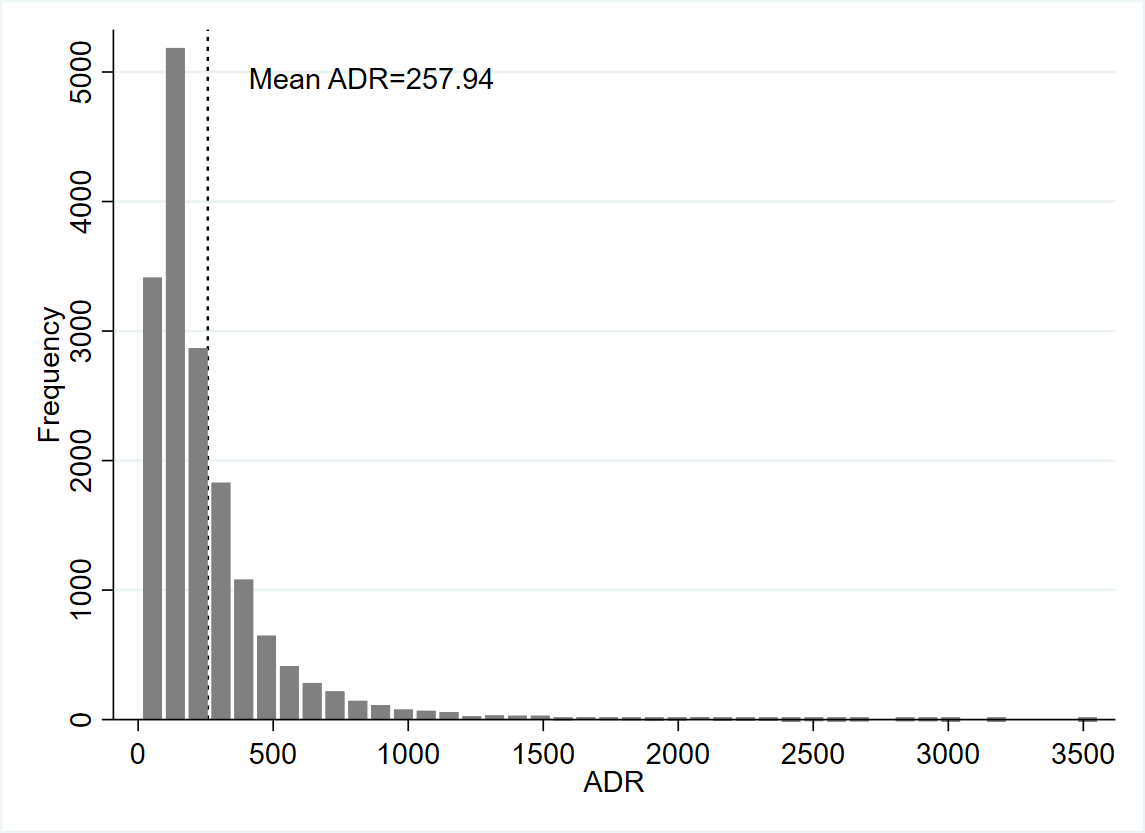


**Figure A1.** Histogram of ADR


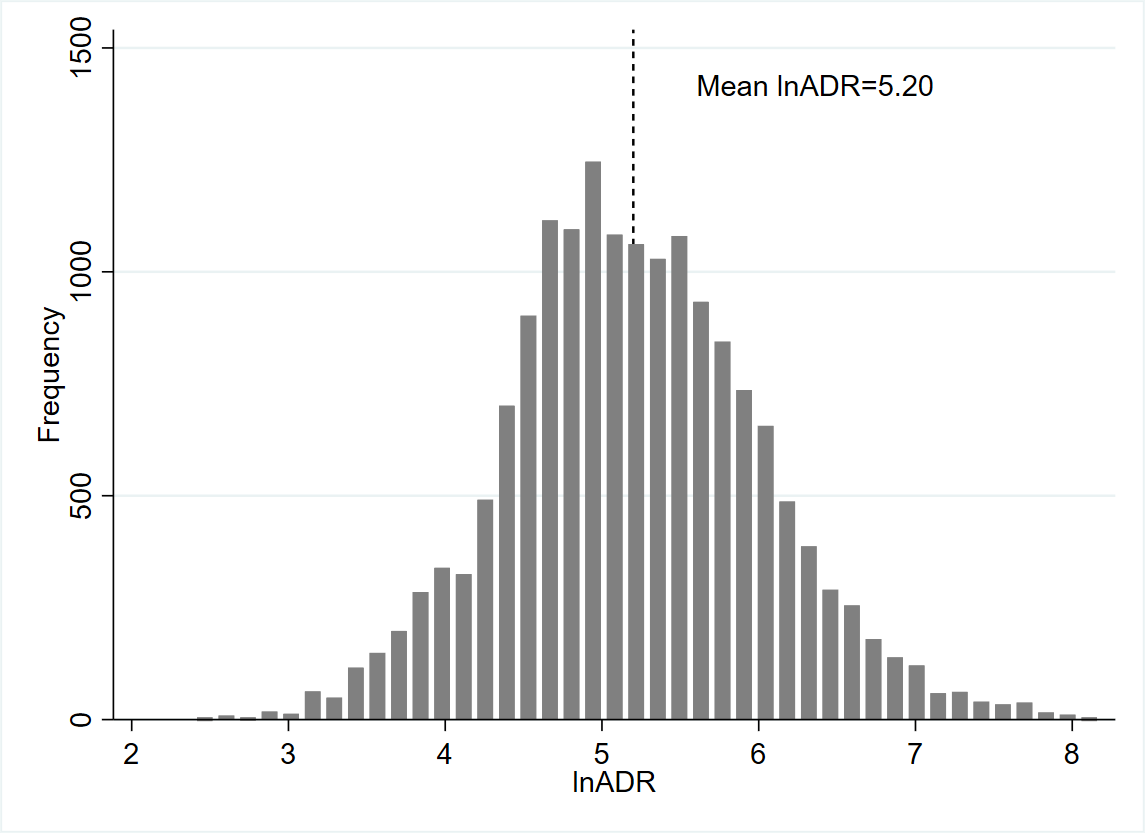


**Figure A2.** Histogram of ln ADR

**
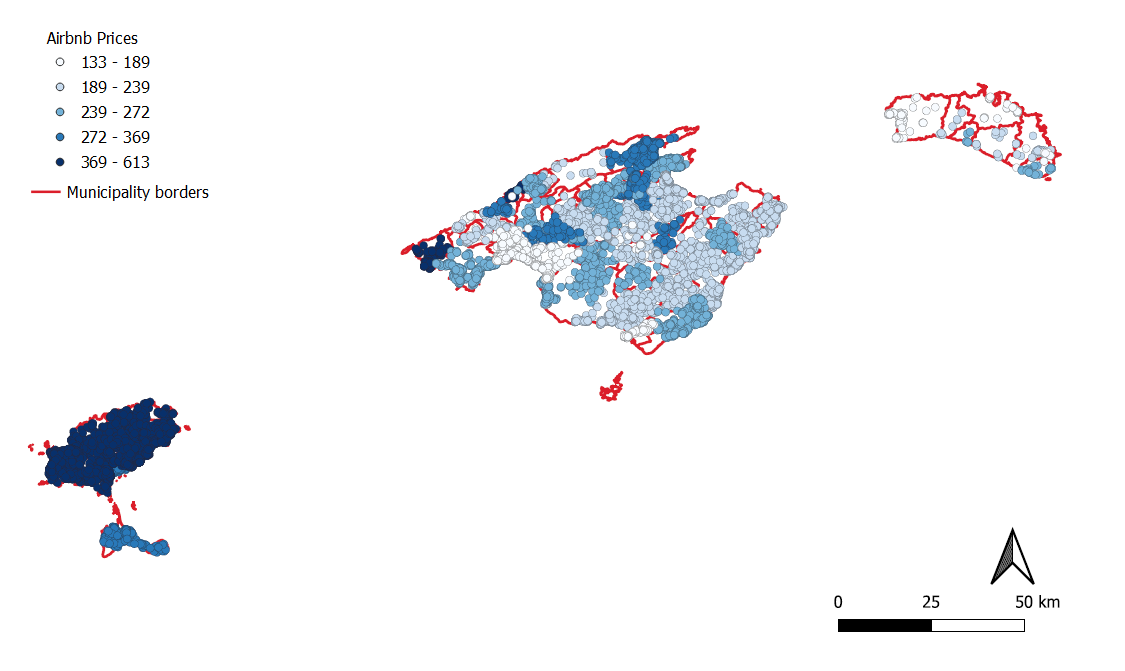
**

**Figure A3.** Average daily rates per municipality (August 2016)


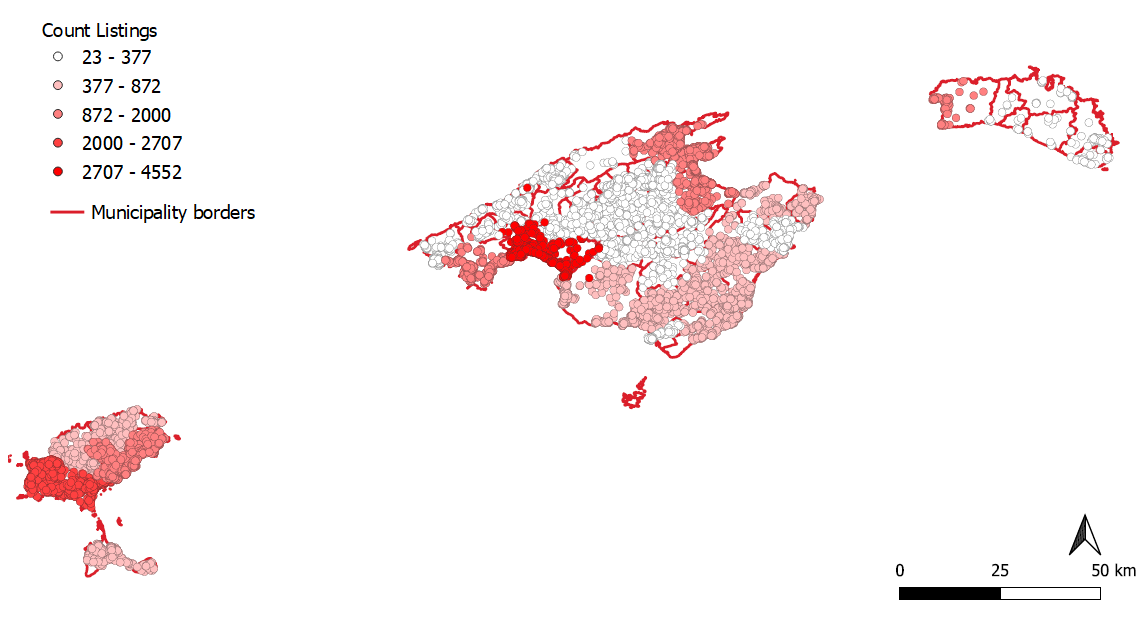


**Figure A4.** Number of properties per municipality (August 2016)

|  | Length | Width | Isolated | Semi-urban | *Urban* | Gold sand | Dark sand | *White sand* | *Urban front.* | Semi-urban front. | Cliff front. | Mount. front. | Dune front. | Calm tide | Veget. | Protec. area | *Easy access* | Diff. access | Only by boat |
| --- | --- | --- | --- | --- | --- | --- | --- | --- | --- | --- | --- | --- | --- | --- | --- | --- | --- | --- | --- |
| Length | 1.000 |  |  |  |  |  |  |  |  |  |  |  |  |  |  |  |  |  |  |
| Width | 0.020 | 1.000 |  |  |  |  |  |  |  |  |  |  |  |  |  |  |  |  |  |
| Isolated | -0.035 | -0.143 | 1.000 |  |  |  |  |  |  |  |  |  |  |  |  |  |  |  |  |
| Semi-urban | -0.074 | 0.164 | -0.582 | 1.000 |  |  |  |  |  |  |  |  |  |  |  |  |  |  |  |
| *Urban* | 0.121 | -0.020 | -0.469 | -0.443 | 1.000 |  |  |  |  |  |  |  |  |  |  |  |  |  |  |
| Gold sand | -0.111 | -0.313 | 0.204 | -0.162 | -0.048 | 1.000 |  |  |  |  |  |  |  |  |  |  |  |  |  |
| Dark sand | 0.002 | -0.119 | 0.062 | 0.044 | -0.116 | -0.222 | 1.000 |  |  |  |  |  |  |  |  |  |  |  |  |
| *White sand* | 0.108 | 0.367 | -0.232 | 0.140 | 0.103 | -0.884 | -0.259 | 1.000 |  |  |  |  |  |  |  |  |  |  |  |
| *Urban front.* | 0.138 | 0.005 | -0.460 | -0.197 | 0.722 | -0.033 | -0.113 | 0.087 | 1.000 |  |  |  |  |  |  |  |  |  |  |
| Semi-urban front. | -0.019 | 0.163 | -0.331 | 0.483 | -0.159 | -0.198 | 0.059 | 0.168 | -0.409 | 1.000 |  |  |  |  |  |  |  |  |  |
| Cliff front. | -0.132 | -0.157 | 0.299 | 0.085 | -0.237 | 0.060 | 0.105 | -0.110 | -0.256 | -0.305 | 1.000 |  |  |  |  |  |  |  |  |
| Mount. front. | -0.168 | 0.122 | 0.487 | -0.248 | -0.268 | 0.206 | -0.029 | -0.190 | -0.263 | -0.314 | -0.196 | 1.000 |  |  |  |  |  |  |  |
| Dune front. | 0.214 | 0.085 | 0.228 | -0.061 | 0.185 | 0.029 | -0.022 | -0.018 | -0.181 | -0.216 | -0.135 | -0.139 | 1.000 |  |  |  |  |  |  |
| Calm tide | 0.049 | 0.078 | -0.093 | 0.034 | 0.065 | -0.159 | -0.132 | 0.221 | 0.051 | 0.034 | -0.102 | -0.041 | 0.052 | 1.000 |  |  |  |  |  |
| Veget. | 0.074 | 0.181 | 0.057 | 0.156 | -0.233 | -0.153 | 0.040 | 0.133 | -0.191 | 0.137 | -0.230 | 0.203 | 0.097 | -0.109 | 1.000 |  |  |  |  |
| Protec. area | 0.168 | 0.034 | 0.418 | -0.150 | 0.298 | 0.078 | 0.077 | -0.115 | -0.306 | -0.161 | 0.132 | 0.179 | 0.333 | -0.175 | 0.207 | 1.000 |  |  |  |
| *Easy access* | 0.169 | 0.130 | -0.361 | 0.150 | 0.234 | -0.179 | 0.053 | 0.152 | 0.230 | 0.250 | -0.317 | -0.363 | 0.121 | 0.201 | -0.008 | -0.248 | 1.000 |  |  |
| Diff. access | -0.144 | -0.081 | 0.306 | -0.132 | -0.194 | 0.137 | -0.028 | -0.122 | -0.190 | -0.227 | 0.247 | 0.340 | -0.100 | -0.137 | 0.086 | 0.220 | -0.827 | 1.000 |  |
| Only by boat | 0.060 | -0.125 | 0.195 | -0.113 | -0.091 | 0.124 | -0.039 | -0.104 | -0.089 | -0.107 | 0.211 | 0.067 | -0.047 | -0.124 | -0.166 | 0.099 | -0.389 | -0.049 | 1.000 |

**Table A1.** Correlation matrix for beach characteristics (N=262)

Note: The variables in italics are the excluded category for the regressions

| Dependent variable: ADR | (1) | (2) | (3) | (4) |
| --- | --- | --- | --- | --- |
| Lambda | -0.027*** | -0.028*** | -0.027*** | -0.027*** |
|  | (0.005) | (0.005) | (0.005) | (0.005) |
| Beach variables | YES | YES | YES | YES |
| Structural characteristics | YES | YES | YES | YES |
| Host characteristics | YES | YES | YES | YES |
| Municipality fixed effects | YES | YES | YES | YES |
| Observations | 16,663 | 16,663 | 16,663 | 16,663 |

**Table A2.** Box Cox regressions assuming a common lambda parameter. Standard errors in parentheses. *** p<0.01, ** p<0.05, * p<0.1

Note: Column 1 reports the results from a common transformation for ADR, Length, Width and Distance. Column 2 only computes the required transformation for ADR and Length; Column 3 only for ADR and Width; Column 4 only for ADR and Distance.

The estimated value of lambda is in all cases statistically significant. Because the point estimate is closer to zero than to one, the Box Cox regression provides greater support for a log-log model (Cameron and Trivedi, 2009, p.94).


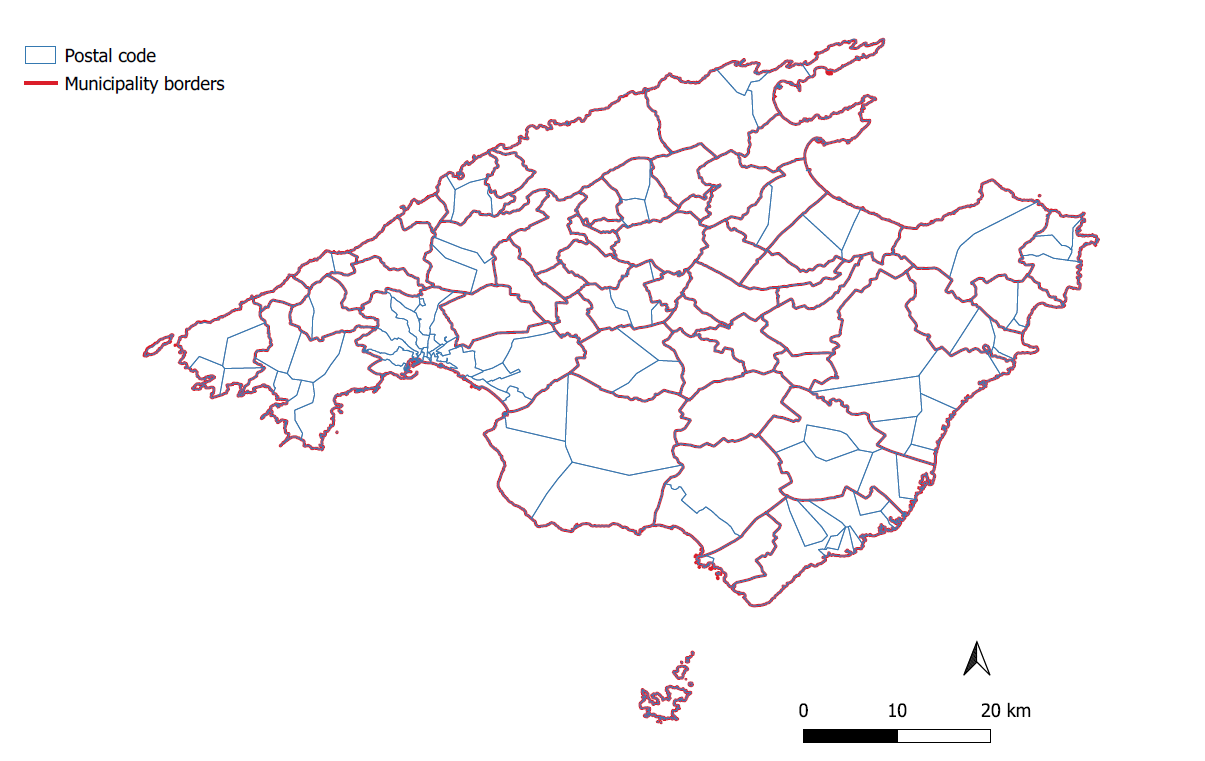


**Figure A5.** Municipalities and postal code areas of Mallorca Island


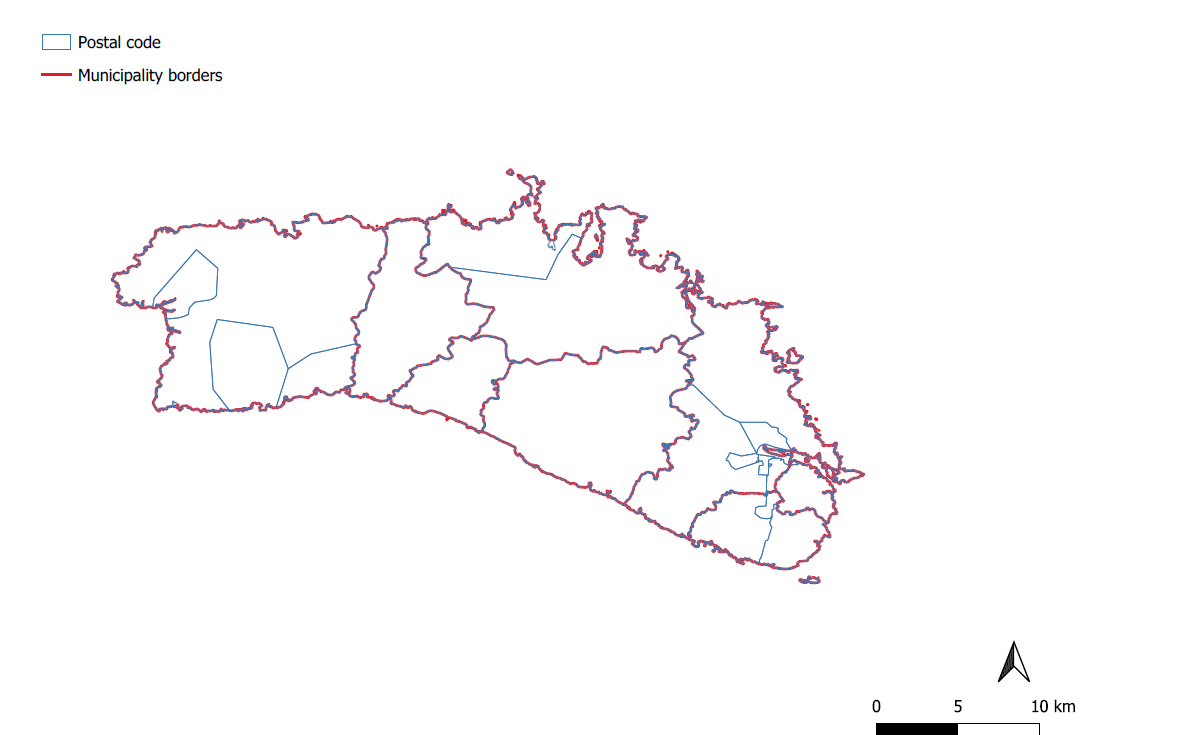


**Figure A6.** Municipalities and postal code areas of Menorca Island


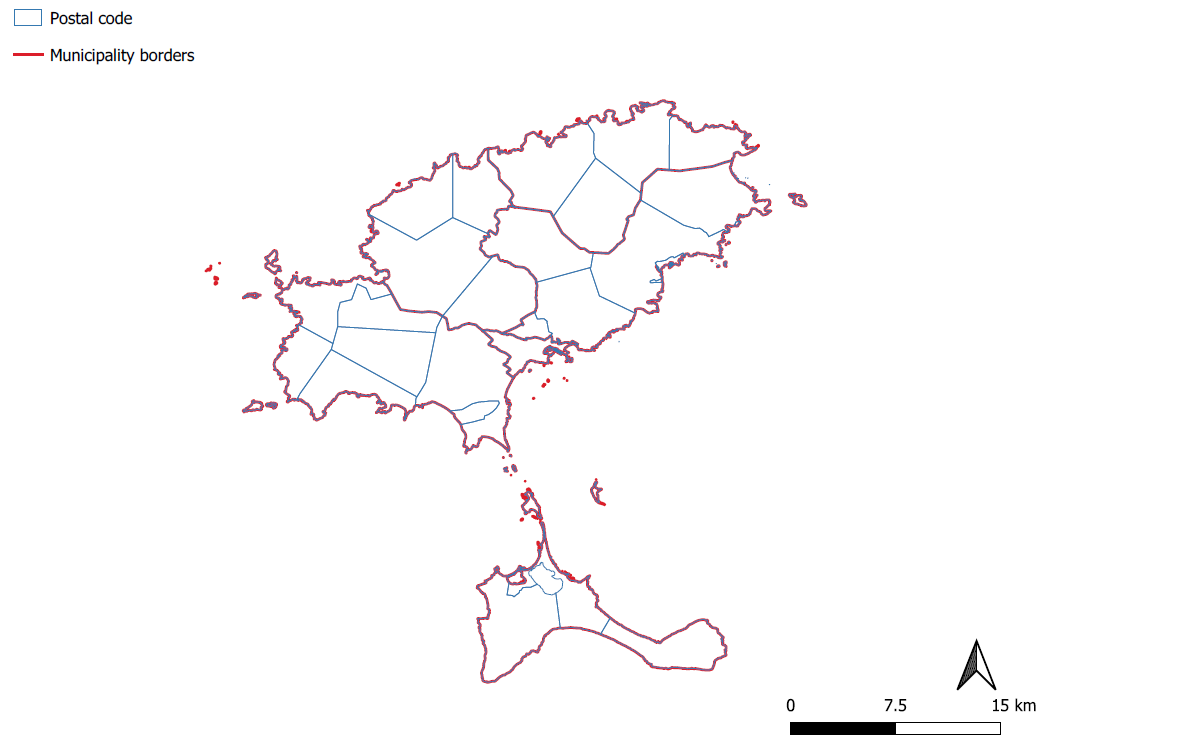


**Figure A7.** Municipalities and postal code areas of Ibiza and Formentera Islands

**Figure A8.** Binscatter regression plot of ln ADR on ln Distance.


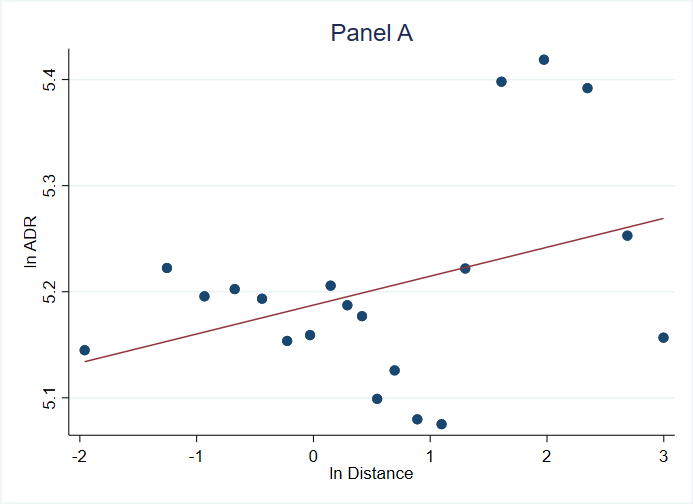

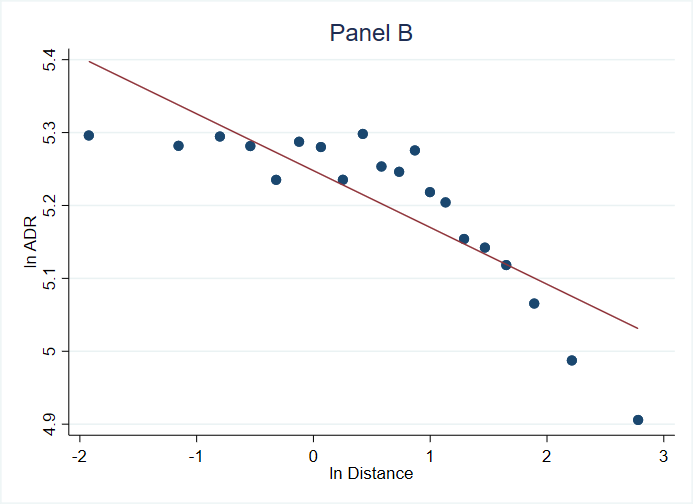

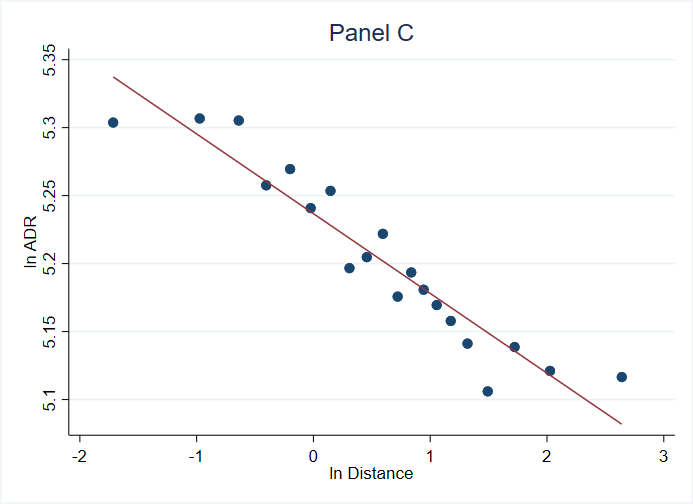

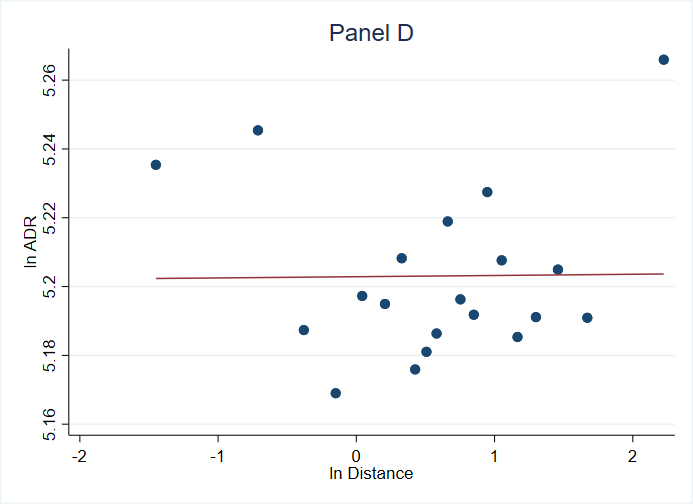


Note: Panel A presents the unconditional relationship between ln ADR and ln Distance to the shoreline. Panel B controls for listing and host characteristics. Panel C adds beach characteristics as an additional control. Panel D further adds municipality fixed effects. The inclusion of municipality fixed effects reverts the negative relationship between ln ADR and ln Distance to the shoreline as they capture level differences in listings’ closeness to the shoreline across municipalities (see Table A3 below).


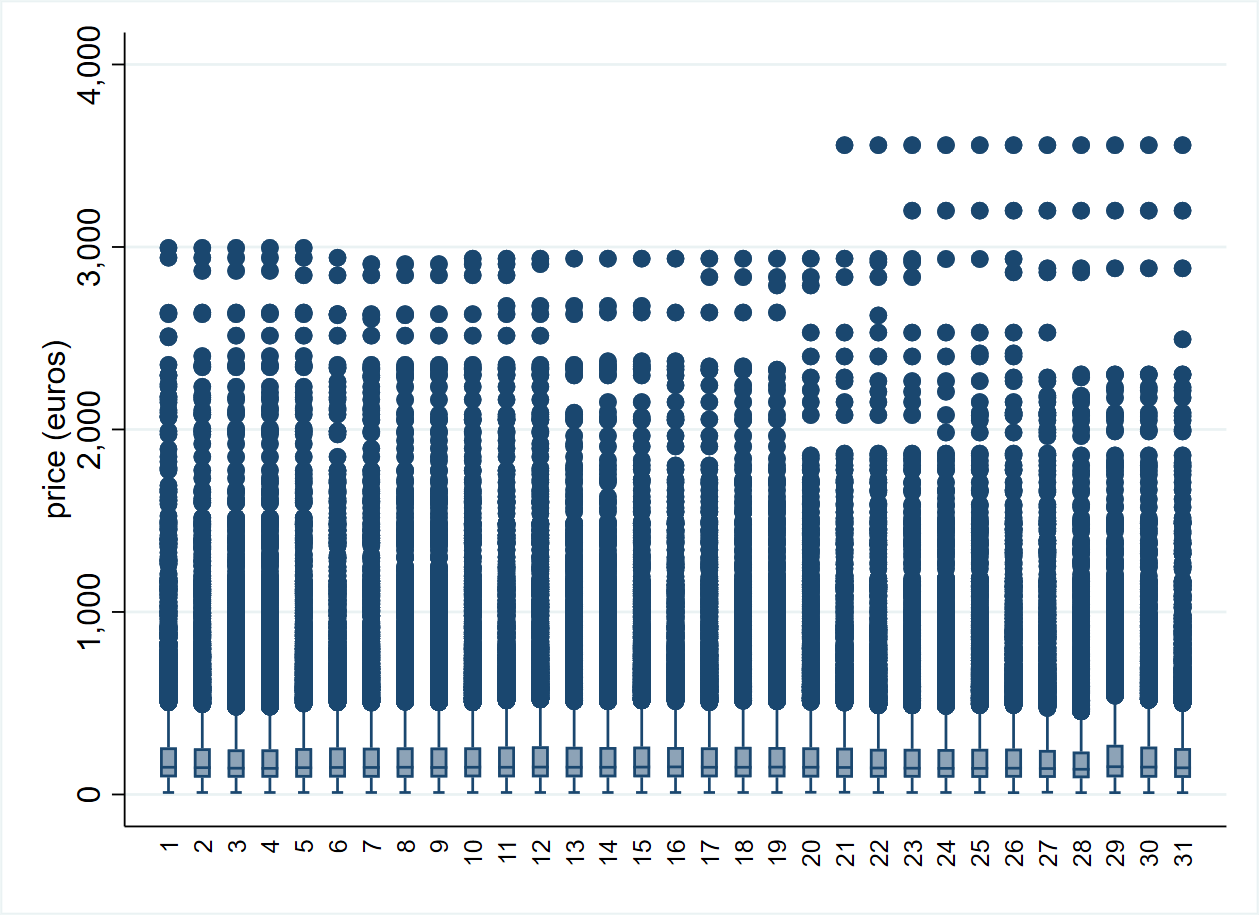


**Figure A9.** Boxplots of daily prices


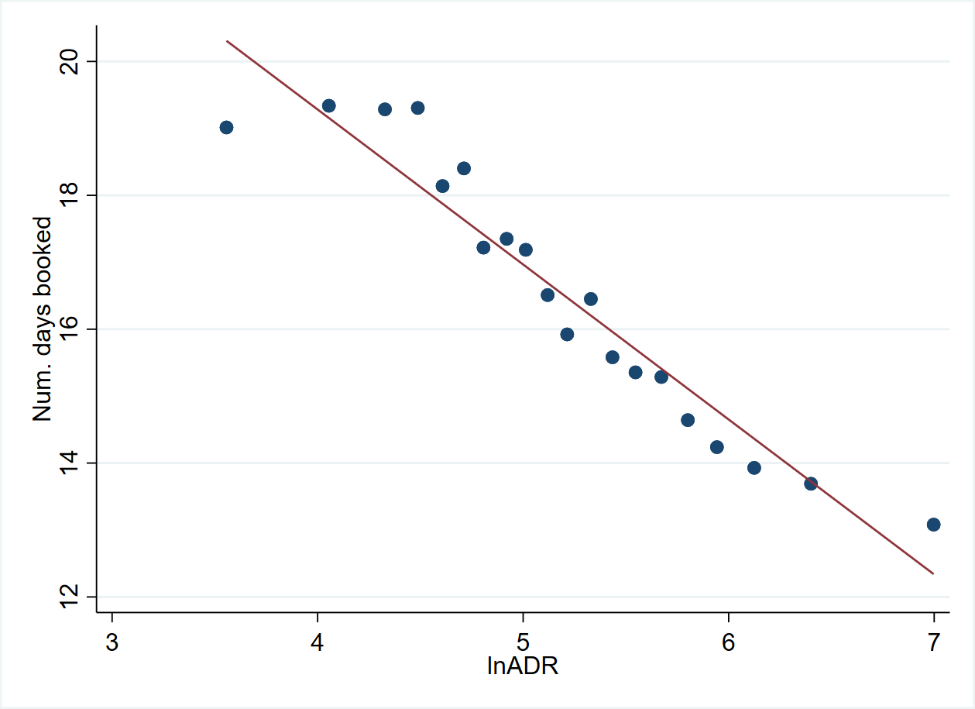


**Figure A10.** Binscatter relationship between the number of days the property is booked and the (log of) ADR


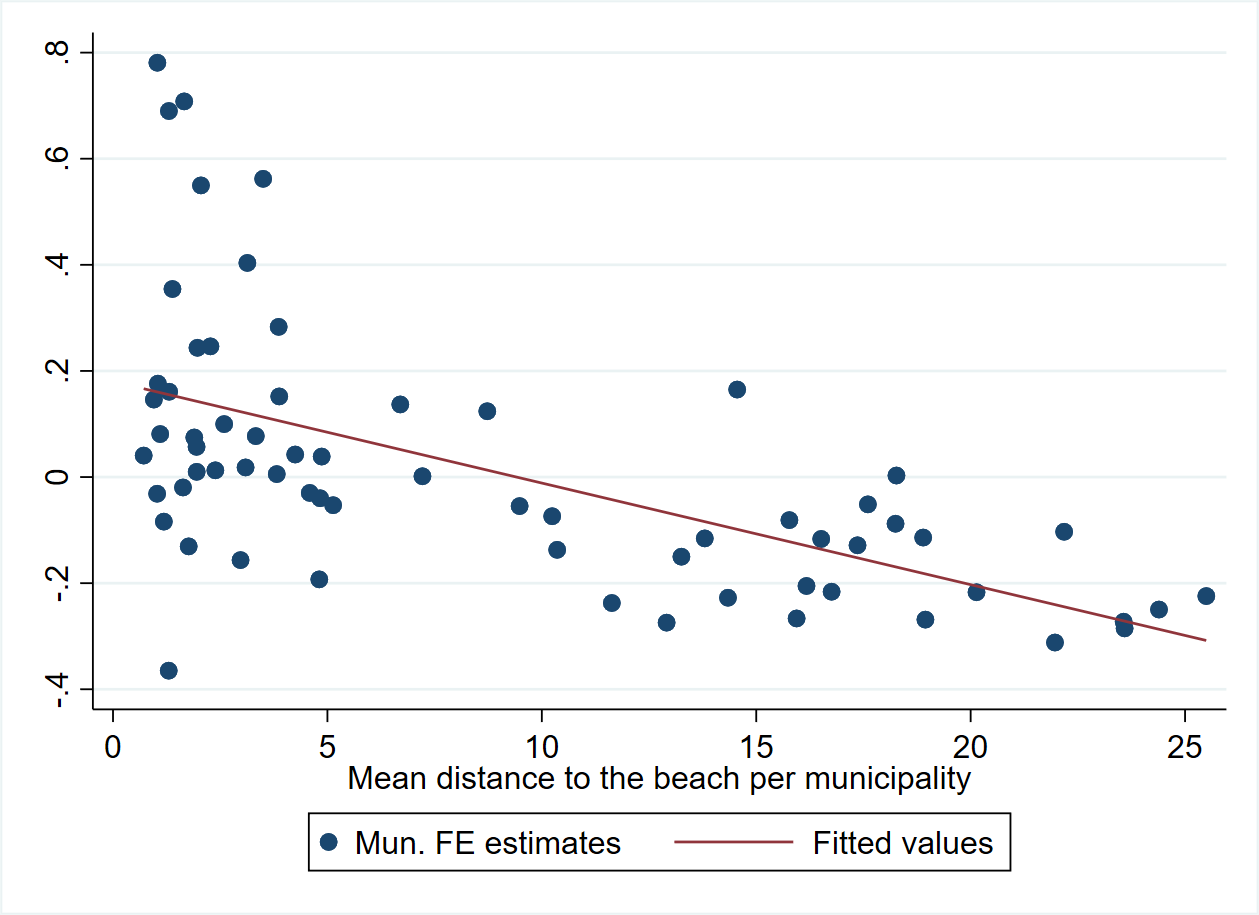


**Figure A11.** Scatterplot of the municipality fixed effects versus the mean distance to the beach per municipality

| **Municipality** | **#distinct beaches** | **#Airbnb properties** | **Mean distance to the shoreline (km)** |
| --- | --- | --- | --- |
| *Alaró* | 2 | 68 | 14.712 |
| *Alcudia* | 6 | 809 | 1.632 |
| *Algaida* | 3 | 108 | 17.362 |
| *Andrach* | 13 | 173 | 1.969 |
| *Ariany* | 3 | 28 | 13.254 |
| *Artá* | 10 | 218 | 3.819 |
| *Bañalbufar* | 1 | 28 | 0.715 |
| *Binisalem* | 4 | 76 | 18.893 |
| *Búger* | 3 | 81 | 16.757 |
| *Buñola* | 8 | 76 | 10.242 |
| *Calviá* | 13 | 661 | 1.047 |
| *Campanet* | 3 | 82 | 15.942 |
| *Campos* | 12 | 516 | 4.588 |
| *Capdepera* | 12 | 251 | 1.029 |
| *Consell* | 2 | 19 | 18.270 |
| *Costitx* | 5 | 52 | 24.392 |
| *Deyá* | 3 | 82 | 1.386 |
| *Escorca* | 4 | 19 | 4.249 |
| *Esporlas* | 3 | 88 | 4.811 |
| *Estellenchs* | 1 | 17 | 1.100 |
| *Felanich* | 8 | 240 | 7.216 |
| *Fornalutx* | 2 | 57 | 5.133 |
| *Inca* | 3 | 135 | 18.945 |
| *Lloret de Vista Alegre* | 2 | 25 | 25.495 |
| *Lloseta* | 2 | 51 | 16.172 |
| *Llubí* | 1 | 91 | 16.515 |
| *Lluchmayor* | 7 | 319 | 4.866 |
| *Manacor* | 19 | 481 | 4.830 |
| *Mancor del Valle* | 1 | 30 | 12.910 |
| *María de la Salud* | 2 | 37 | 13.800 |
| *Marrachí* | 4 | 98 | 9.482 |
| *Montuiri* | 1 | 50 | 23.568 |
| *Muro* | 1 | 69 | 10.358 |
| *Palma de Mallorca* | 12 | 2,933 | 1.951 |
| *Petra* | 3 | 34 | 18.248 |
| *Pollensa* | 8 | 879 | 3.330 |
| *Porreras* | 4 | 55 | 17.604 |
| *La Puebla* | 3 | 173 | 11.633 |
| *Puigpuñent* | 3 | 43 | 6.700 |
| *Las Salinas* | 8 | 177 | 1.765 |
| *San Juan* | 4 | 35 | 23.591 |
| *San Lorenzo de Cardessar* | 9 | 83 | 8.728 |
| *Sancellas* | 3 | 75 | 21.967 |
| *Santa Eugenia* | 1 | 36 | 15.773 |
| *Santa Margarita* | 7 | 530 | 2.974 |
| *Santa María del Camino* | 4 | 51 | 14.556 |
| *Santañy* | 14 | 437 | 1.895 |
| *Selva* | 2 | 157 | 14.341 |
| *Sineu* | 2 | 53 | 20.137 |
| *Sóller* | 3 | 250 | 2.592 |
| *Son Servera* | 9 | 223 | 1.186 |
| *Valldemosa* | 4 | 95 | 3.091 |
| *Villafranca de Bonany* | 3 | 42 | 22.180 |
| *Eivissa* | 5 | 1,59 | 1.034 |
| *Santa Eulalia del Río* | 26 | 1,054 | 2.051 |
| *Sant Joan de Labritja* | 16 | 262 | 3.133 |
| *Sant Jusep de sa Talaia* | 33 | 1,303 | 1.304 |
| *Sant Antoni de Portmany* | 11 | 406 | 3.501 |
| *Ciutadella* | 12 | 99 | 0.953 |
| *Ferreries* | 3 | 11 | 2.272 |
| *Es Mercadal* | 4 | 25 | 1.948 |
| *Es Migjorn Gran* | 2 | 6 | 1.300 |
| *Alaior* | 3 | 15 | 3.864 |
| *Maó* | 5 | 43 | 3.876 |
| *Es Castell* | 2 | 12 | 2.386 |
| *Sant Lluis* | 4 | 26 | 1.309 |
| *Formentera* | 12 | 315 | 1.660 |

**Table A3.** List of municipalities in the sample, number of beaches and properties per municipality, and mean distance to the shoreline per municipality.

| Dependent variable: Ln ADR | (1) |  | (2) |
| --- | --- | --- | --- |
| Explanatory variables | Coeff. (SE) |  | Coeff. (SE) |
| Ln Distance | 0.001 |  | 0.029 |
|  | (0.007) |  | (0.039) |
| Distance dummy: 200 metres |  | 0.033 |  |
|  |  | (0.027) |  |
| Distance dummy: 400 metres |  | 0.031 |  |
|  |  | (0.026) |  |
| Distance dummy: 500 metres |  | -0.024 |  |
|  |  | (0.026) |  |
| Distance dummy: 1000 metres |  | -0.033* |  |
|  |  | (0.019) |  |
| Distance dummy: 1500 metres |  | -0.021 |  |
|  |  | (0.029) |  |
| Distance dummy: 2000 metres |  | 0.005 |  |
|  |  | (0.026) |  |
| Distance dummy: 2500 metres |  | 0.017 |  |
|  |  | (0.026) |  |
| Ln Length | 0.027*** | 0.029*** | 0.028*** |
|  | (0.010) | (0.010) | (0.009) |
| Ln Length x Ln distance |  |  | -0.007 |
|  |  |  | (0.006) |
| Ln Width | -0.016 | -0.016 | -0.008 |
|  | (0.013) | (0.013) | (0.013) |
| Ln Width x Ln distance |  |  | -0.015 |
|  |  |  | (0.009) |
| Gold sand | 0.014 | 0.014 | 0.010 |
|  | (0.025) | (0.025) | (0.025) |
| Gold sand x Ln Distance |  |  | 0.019 |
|  |  |  | (0.017) |
| Dark sand | -0.040 | -0.042 | -0.052 |
|  | (0.043) | (0.043) | (0.038) |
| Dark sand x Ln Distance |  |  | 0.021 |
|  |  |  | (0.023) |
| Cliff front. | 0.094*** | 0.097*** | 0.092*** |
|  | (0.033) | (0.034) | (0.034) |
| Cliff front. x Ln Distance |  |  | -0.014 |
|  |  |  | (0.027) |
| Semi-urban front. | 0.116*** | 0.121*** | 0.119*** |
|  | (0.038) | (0.038) | (0.036) |
| Semi-urban front. x Ln Distance |  |  | 0.008 |
|  |  |  | (0.017) |
| Mountain front. | 0.090* | 0.092** | 0.129*** |
|  | (0.046) | (0.045) | (0.046) |
| Mountain front. x Ln Distance |  |  | -0.059* |
|  |  |  | (0.030) |
| Dune front. | 0.112** | 0.119*** | 0.103** |
|  | (0.044) | (0.043) | (0.046) |
| Dune front. x Ln Distance |  |  | 0.017 |
|  |  |  | (0.031) |
| Calm tide | -0.022 | -0.023 | -0.019 |
|  | (0.022) | (0.022) | (0.023) |
| Calm tide x Ln Distance |  |  | -0.006 |
|  |  |  | (0.013) |
| Vegetation | 0.051** | 0.054** | 0.045** |
|  | (0.022) | (0.023) | (0.020) |
| Vegetation x Ln Distance |  |  | 0.026* |
|  |  |  | (0.014) |
| Protec. area | -0.007 | -0.012 | 0.030 |
|  | (0.026) | (0.026) | (0.027) |
| Protect. area x Ln Distance |  |  | -0.037** |
|  |  |  | (0.017) |
| Diff. access | 0.113*** | 0.116*** | 0.206*** |
|  | (0.043) | (0.042) | (0.041) |
| Diff. access x Ln Distance |  |  | -0.106*** |
|  |  |  | (0.027) |
| Only by boat | 0.094* | 0.100* | 0.054 |
|  | (0.055) | (0.054) | (0.057) |
| Only by boat x Ln Distance |  |  | 0.020 |
|  |  |  | (0.043) |
| Isolated envir. | -0.069* | -0.064 | -0.100*** |
|  | (0.039) | (0.039) | (0.037) |
| Isolated envir. x Ln Distance |  |  | 0.026 |
|  |  |  | (0.026) |
| Semi-urban envir. | -0.075** | -0.072** | -0.078** |
|  | (0.033) | (0.034) | (0.031) |
| Semi-urban envir. x Ln Distance |  |  | 0.019 |
|  |  |  | (0.018) |
| Apartment | -0.107*** | -0.107*** | -0.108*** |
|  | (0.023) | (0.022) | (0.023) |
| House | 0.037 | 0.037 | 0.033 |
|  | (0.023) | (0.024) | (0.024) |
| Villa | 0.283*** | 0.284*** | 0.278*** |
|  | (0.026) | (0.026) | (0.026) |
| Chalet | 0.100*** | 0.099*** | 0.094*** |
|  | (0.026) | (0.026) | (0.026) |
| Entire | 0.652*** | 0.650*** | 0.651*** |
|  | (0.036) | (0.035) | (0.037) |
| Minimum stay | 0.008*** | 0.008*** | 0.008*** |
|  | (0.003) | (0.003) | (0.003) |
| Bedrooms | 0.265*** | 0.266*** | 0.265*** |
|  | (0.009) | (0.009) | (0.009) |
| Number of photos | 0.003*** | 0.003*** | 0.003*** |
|  | (0.001) | (0.001) | (0.001) |
| Never rated | 0.274*** | 0.274*** | 0.274*** |
|  | (0.019) | (0.019) | (0.019) |
| High rated | 0.088*** | 0.087*** | 0.088*** |
|  | (0.009) | (0.009) | (0.009) |
| Low rated | 0.060*** | 0.060*** | 0.061*** |
|  | (0.014) | (0.014) | (0.014) |
| Canc. policy: Moderate | -0.025 | -0.024 | -0.024 |
|  | (0.017) | (0.017) | (0.017) |
| Canc. Policy: Strict | 0.115*** | 0.115*** | 0.114*** |
|  | (0.012) | (0.012) | (0.012) |
| Instant booking | -0.038*** | -0.038*** | -0.037*** |
|  | (0.012) | (0.012) | (0.012) |
| Superhost | 0.002 | 0.002 | 0.004 |
|  | (0.018) | (0.018) | (0.018) |
| Host experience | 5.2e-05*** | 5.2e-05*** | -5.2e-05*** |
|  | (1.5e-05) | (1.5e-05) | (1.5e-05) |
| Host number of listings | 8.5e-05** | 8.5e-05** | 8.8e-05** |
|  | (3.9e-05) | (3.9e-05) | (3.9e-05) |
| Municipality fixed effects | YES |  | YES |
| Constant | 3.592*** |  | 3.569*** |
|  | (0.073) |  | (0.073) |
| Observations | 16,663 |  | 16,663 |
| R-squared | 0.746 |  | 0.747 |

**Table A4.** WLS hedonic price regression estimates (full) with and without interaction terms (Table 3 in the text). Clustered standard errors at the beach level in parentheses. *** p<0.01, ** p<0.05, * p<0.1

Note: The reference categories are *Clear sand*, *Urban front*, *Easy Ac, Urban envir., Other, Shared/private, Medium rate* and *Flexible Canc.*

|  | (1) | (2) | (3) |
| --- | --- | --- | --- |
| Dependent variable: Ln ADR | Less than 100 m. | Less than 200 m | Less than 300 m. |
| Explanatory variables | Coeff. (SE) | Coeff. (SE) | Coeff. (SE) |
|  |  |  |  |
| Ln Distance | 0.070 | -0.050 | -0.013 |
|  | (0.069) | (0.033) | (0.020) |
| Ln Length | 0.088* | 0.024 | 0.022 |
|  | (0.048) | (0.025) | (0.019) |
| Ln Width | 0.040 | 0.036 | 0.047*** |
|  | (0.064) | (0.025) | (0.018) |
| Gold sand | 0.047 | 0.058 | 0.017 |
|  | (0.146) | (0.059) | (0.042) |
| Dark sand | -0.330 | 0.026 | -0.086 |
|  | (0.365) | (0.106) | (0.067) |
| Cliff front. | 0.190 | -0.051 | -0.001 |
|  | (0.222) | (0.097) | (0.070) |
| Semi-urban front. | 0.286* | 0.112* | 0.060* |
|  | (0.168) | (0.067) | (0.035) |
| Mountain front. | 0.371 | 0.124 | 0.031 |
|  | (0.253) | (0.126) | (0.097) |
| Dune front. | 1.324** | -0.262* | 0.048 |
|  | (0.658) | (0.156) | (0.104) |
| Calm tide | -0.036 | -0.083 | -0.114** |
|  | (0.135) | (0.065) | (0.052) |
| Vegetation | 0.173* | 0.076* | 0.070* |
|  | (0.101) | (0.045) | (0.038) |
| Protec. area | -0.678 | -0.096 | -0.093 |
|  | (0.518) | (0.120) | (0.075) |
| Diff. access | 0.112 | 0.196 | 0.290*** |
|  | (0.379) | (0.122) | (0.107) |
| Isolated envir. | -0.279 | -0.096 | 0.094 |
|  | (0.205) | (0.123) | (0.081) |
| Semi-urban envir. | -0.169 | -0.121** | -0.087** |
|  | (0.134) | (0.053) | (0.036) |
| Structural characteristics | YES | YES | YES |
| Host characteristics | YES | YES | YES |
| Municipality fixed effects | YES | YES | YES |
| Constant | 3.421*** | 3.120*** | 3.388*** |
|  | (0.659) | (0.213) | (0.140) |
| Number of matched beaches | 71 | 126 | 163 |
| Observations | 150 | 620 | 1,360 |
| R-squared | 0.795 | 0.736 | 0.728 |

**Table A5.** WLS hedonic price regression estimates considering listings in close proximity to the beach. Clustered standard errors at the beach level in parentheses. *** p<0.01, ** p<0.05, * p<0.1

Note: The reference categories are *Clear sand*, *Urban front*, *Easy Ac* and *Urban envir*

|  | Less 100 metres | | Less 200 metres | | Less 500 metres | | Less 1km | | Less 2km | | All | |
| --- | --- | --- | --- | --- | --- | --- | --- | --- | --- | --- | --- | --- |
| Variable | Mean  (%) | SD | Mean  (%) | SD | Mean  (%) | SD | Mean  (%) | SD | Mean  (%) | SD | Mean  (%) | SD |
| Apartment | 73.33 |  | 72.74 |  | 68.10 |  | 62.39 |  | 61.73 |  | 45.21 |  |
| House | 18.66 |  | 17.09 |  | 18.81 |  | 22.04 |  | 22.22 |  | 34.43 |  |
| Villa | 3.33 |  | 3.87 |  | 4.93 |  | 6.42 |  | 6.75 |  | 10.35 |  |
| Chalet | 0.00 |  | 0.96 |  | 1.74 |  | 2.05 |  | 1.88 |  | 2.37 |  |
| Other | 4.66 |  | 6.30 |  | 8.14 |  | 9.14 |  | 9.23 |  | 7.64 |  |
| Entire | 91.33 |  | 89.03 |  | 87.12 |  | 84.54 |  | 83.88 |  | 83.34 |  |
| Shared/private | 8.66 |  | 10.96 |  | 12.86 |  | 15.44 |  | 16.11 |  | 16.66 |  |
| Min. Stay | 4.07 | 2.40 | 3.91 | 2.31 | 3.77 | 2.73 | 3.75 | 2.45 | 3.70 | 2.31 | 3.86 | 2.29 |
| Bedrooms | 2.20 | 1.28 | 2.03 | 1.22 | 2.07 | 1.20 | 2.14 | 1.29 | 2.145 | 1.31 | 2.38 | 1.45 |
| Num. Photos | 20.42 | 10.73 | 20.78 | 11.54 | 20.48 | 12.12 | 20.66 | 12.51 | 20.66 | 12.44 | 22.71 | 15.70 |
| Never rated | 26.00 |  | 19.51 |  | 24.34 |  | 24.45 |  | 23.89 |  | 27.69 |  |
| High rate | 38.66 |  | 40.00 |  | 36.76 |  | 37.75 |  | 39.66 |  | 41.10 |  |
| Medium rate | 22.66 |  | 24.83 |  | 23.60 |  | 23.12 |  | 23.02 |  | 19.79 |  |
| Low rate | 12.66 |  | 15.64 |  | 15.28 |  | 14.66 |  | 13.40 |  | 11.40 |  |
| Flexible Canc. | 16.66 |  | 16.12 |  | 17.45 |  | 18.07 |  | 17.85 |  | 17.23 |  |
| Moderate Canc. | 10.66 |  | 11.93 |  | 10.94 |  | 11.34 |  | 11.62 |  | 11.87 |  |
| Strict. Canc. | 70.66 |  | 71.12 |  | 70.72 |  | 69.84 |  | 69.83 |  | 69.92 |  |
| Instant Booking | 31.33 |  | 27.41 |  | 24.02 |  | 23.66 |  | 23.41 |  | 25.92 |  |
| Superhost | 4.66 |  | 5.48 |  | 5.38 |  | 6.07 |  | 6.49 |  | 7.38 |  |
| Host Experience | 446.35 | 406.08 | 458.80 | 405.19 | 438.38 | 402.59 | 437.12 | 402.14 | 435.02 | 403.02 | 444.47 | 408.17 |
| Num. listings | 42.97 | 139.78 | 32.97 | 120.12 | 29.48 | 111.13 | 27.14 | 105.16 | 26.10 | 26.10 | 35.58 | 118.58 |
| Observations | 150 | | 620 | | 2,859 | | 5,544 | | 9,592 | | 16,663 | |

**Table A6.** Descriptive statistics of property structural characteristics for subsamples based on distance to the closest beach

|  | (1) | (2) | (3) | (4) | (5) | (6) | (7) |
| --- | --- | --- | --- | --- | --- | --- | --- |
| Dependent variable: Ln ADR | Less than 500 m. | Less than 750 m. | Less than 1,000 m | Less than 2,000 m. | Less than 3,000 m. | Less than 4,000 m | Less than 5,000 m |
| Explanatory variables | Coeff. (SE) | Coeff. (SE) | Coeff. (SE) | Coeff. (SE) | Coeff. (SE) | Coeff. (SE) | Coeff. (SE) |
|  |  |  |  |  |  |  |  |
| Ln Distance | -0.000 | 0.106 | 0.006 | 0.102 | 0.122** | 0.090* | 0.085* |
|  | (0.113) | (0.083) | (0.073) | (0.067) | (0.054) | (0.053) | (0.049) |
| Ln Length | -0.005 | 0.041** | 0.040*** | 0.038*** | 0.038*** | 0.037*** | 0.034*** |
|  | (0.025) | (0.020) | (0.015) | (0.012) | (0.011) | (0.010) | (0.010) |
| Ln Length x Ln distance | -0.015 | 0.012 | 0.011 | 0.008 | 0.009 | 0.006 | 0.004 |
|  | (0.014) | (0.012) | (0.011) | (0.011) | (0.008) | (0.008) | (0.007) |
| Ln Width | 0.019 | -0.037 | -0.013 | -0.028* | -0.023 | -0.019 | -0.014 |
|  | (0.043) | (0.029) | (0.024) | (0.016) | (0.014) | (0.013) | (0.013) |
| Ln Width x Ln distance | -0.012 | -0.047** | -0.029 | -0.042** | -0.040*** | -0.033*** | -0.029** |
|  | (0.026) | (0.019) | (0.018) | (0.017) | (0.013) | (0.013) | (0.011) |
| Gold sand | -0.008 | -0.027 | 0.005 | -0.002 | -0.009 | -0.002 | -0.004 |
|  | (0.082) | (0.058) | (0.048) | (0.025) | (0.024) | (0.024) | (0.024) |
| Gold sand x Ln Distance | 0.003 | -0.009 | 0.011 | -0.004 | -0.020 | -0.010 | -0.011 |
|  | (0.049) | (0.034) | (0.028) | (0.029) | (0.024) | (0.022) | (0.021) |
| Dark sand | -0.152 | -0.022 | -0.042 | -0.067 | -0.074** | -0.073** | -0.078** |
|  | (0.165) | (0.110) | (0.069) | (0.041) | (0.034) | (0.034) | (0.035) |
| Dark sand x Ln Distance | -0.034 | 0.068 | 0.048 | 0.010 | -0.023 | -0.002 | -0.008 |
|  | (0.102) | (0.079) | (0.051) | (0.039) | (0.038) | (0.038) | (0.038) |
| Cliff front. | 0.074 | 0.033 | 0.111** | 0.104** | 0.109*** | 0.121*** | 0.114*** |
|  | (0.082) | (0.054) | (0.054) | (0.045) | (0.040) | (0.037) | (0.036) |
| Cliff front. x Ln Distance | -0.001 | -0.025 | 0.046 | 0.040 | 0.047 | 0.054* | 0.039 |
|  | (0.055) | (0.041) | (0.038) | (0.034) | (0.032) | (0.029) | (0.027) |
| Semi-urban front. | -0.130* | -0.021 | 0.041 | 0.148*** | 0.135*** | 0.135*** | 0.138*** |
|  | (0.072) | (0.042) | (0.041) | (0.046) | (0.040) | (0.035) | (0.033) |
| Semi-urban front. x Ln Distance | -0.088* | -0.027 | 0.009 | 0.076* | 0.057* | 0.055** | 0.052** |
|  | (0.048) | (0.034) | (0.039) | (0.046) | (0.033) | (0.027) | (0.023) |
| Mountain front. | -0.030 | 0.227** | 0.240*** | 0.181*** | 0.161*** | 0.145*** | 0.146*** |
|  | (0.182) | (0.105) | (0.091) | (0.063) | (0.055) | (0.048) | (0.047) |
| Mountain front. x Ln Distance | -0.071 | 0.067 | 0.089 | 0.027 | 0.016 | -0.024 | -0.025 |
|  | (0.095) | (0.076) | (0.074) | (0.057) | (0.046) | (0.041) | (0.040) |
| Dune front. | 0.177 | -0.127 | 0.084 | 0.148** | 0.127** | 0.123** | 0.132** |
|  | (0.282) | (0.198) | (0.126) | (0.058) | (0.059) | (0.056) | (0.051) |
| Dune front. x Ln Distance | 0.075 | -0.213 | -0.031 | 0.018 | -0.054 | -0.012 | -0.001 |
|  | (0.247) | (0.230) | (0.167) | (0.085) | (0.065) | (0.063) | (0.055) |
| Calm tide | -0.125** | -0.038 | 0.007 | -0.018 | -0.017 | -0.022 | -0.030 |
|  | (0.062) | (0.051) | (0.037) | (0.027) | (0.026) | (0.024) | (0.023) |
| Calm tide x Ln Distance | 0.001 | 0.039 | 0.060** | 0.017 | 0.007 | 0.003 | -0.010 |
|  | (0.036) | (0.031) | (0.027) | (0.025) | (0.018) | (0.017) | (0.016) |
| Vegetation | 0.067 | 0.042 | 0.067 | 0.037 | 0.028 | 0.036 | 0.036 |
|  | (0.064) | (0.050) | (0.043) | (0.029) | (0.025) | (0.022) | (0.022) |
| Vegetation x Ln Distance | -0.014 | -0.019 | 0.006 | -0.003 | -0.011 | 0.008 | 0.010 |
|  | (0.037) | (0.028) | (0.024) | (0.022) | (0.019) | (0.018) | (0.017) |
| Protec. area | 0.055 | -0.019 | -0.040 | -0.018 | 0.004 | 0.015 | 0.013 |
|  | (0.125) | (0.080) | (0.056) | (0.039) | (0.034) | (0.031) | (0.029) |
| Protect. area x Ln Distance | 0.130 | 0.073 | 0.037 | 0.006 | 0.002 | -0.014 | -0.025 |
|  | (0.083) | (0.057) | (0.042) | (0.030) | (0.026) | (0.027) | (0.025) |
| Diff. access | 0.625*** | 0.238* | 0.196* | 0.233*** | 0.218*** | 0.220*** | 0.211*** |
|  | (0.178) | (0.144) | (0.115) | (0.064) | (0.052) | (0.043) | (0.043) |
| Diff. access x Ln Distance | 0.218** | -0.012 | -0.055 | -0.046 | -0.088* | -0.096* | -0.109** |
|  | (0.099) | (0.109) | (0.102) | (0.062) | (0.047) | (0.054) | (0.044) |
| Only by boat | -5.116*** | -0.065 | -0.114 | 0.017 | 0.052 | 0.066 | 0.070 |
|  | (0.793) | (0.281) | (0.117) | (0.087) | (0.078) | (0.070) | (0.069) |
| Only by boat x Ln Distance | -5.583*** | -0.493 | -0.723*** | -0.305*** | -0.250*** | -0.158* | -0.117 |
|  | (0.859) | (0.468) | (0.099) | (0.095) | (0.092) | (0.094) | (0.083) |
| Isolated envir. | -0.067 | -0.012 | -0.084 | -0.071 | -0.067 | -0.069* | -0.076* |
|  | (0.104) | (0.083) | (0.068) | (0.052) | (0.046) | (0.041) | (0.040) |
| Isolated envir. x Ln Distance | -0.061 | -0.013 | -0.069 | -0.026 | 0.011 | 0.032 | 0.028 |
|  | (0.077) | (0.074) | (0.067) | (0.047) | (0.038) | (0.037) | (0.035) |
| Semi-urban envir. | -0.065 | 0.016 | -0.020 | -0.079* | -0.084** | -0.081** | -0.078** |
|  | (0.072) | (0.047) | (0.043) | (0.046) | (0.039) | (0.034) | (0.033) |
| Semi-urban envir. x Ln Distance | 0.013 | 0.064** | 0.030 | -0.018 | -0.008 | -0.001 | 0.001 |
|  | (0.045) | (0.029) | (0.027) | (0.036) | (0.028) | (0.024) | (0.022) |
| Municipality fixed effects | YES | YES | YES | YES | YES | YES | YES |
| Constant | 3.448*** | 3.683*** | 3.517*** | 3.572*** | 3.562*** | 3.537*** | 3.522*** |
|  | (0.201) | (0.153) | (0.122) | (0.078) | (0.071) | (0.068) | (0.067) |
| Number of matched beaches | 195 | 213 | 222 | 234 | 240 | 249 | 255 |
| Observations | 2,859 | 4,329 | 5,544 | 9,592 | 11,263 | 12,376 | 12,931 |
| R-squared | 0.733 | 0.727 | 0.733 | 0.749 | 0.760 | 0.765 | 0.767 |

**Table A7.** WLS hedonic price regression estimates considering different distance thresholds. Clustered standard errors at the beach level in parentheses. *** p<0.01, ** p<0.05, * p<0.1

Note: The reference categories are *Clear sand*, *Urban front*, *Easy Ac* and *Urban envir.*

|  | (1) | (2) | (3) | (4) | (5) |
| --- | --- | --- | --- | --- | --- |
| Dependent variable: Ln ADR | Prob  (d_500=1) | Prob  (d_1000=1) | Prob  (d_1500=1) | Prob  (d_2000=1) | Prob  (d_3000=1) |
| Explanatory variables | Coeff. (SE) | Coeff. (SE) | Coeff. (SE) | Coeff. (SE) | Coeff. (SE) |
|  |  |  |  |  |  |
| Apartment | 0.340*** | 0.341*** | 0.446*** | 0.585*** | 0.736*** |
|  | (0.047) | (0.041) | (0.039) | (0.040) | (0.043) |
| House | -0.284*** | -0.311*** | -0.402*** | -0.464*** | -0.478*** |
|  | (0.052) | (0.044) | (0.042) | (0.042) | (0.043) |
| Villa | -0.348*** | -0.320*** | -0.395*** | -0.425*** | -0.421*** |
|  | (0.066) | (0.054) | (0.052) | (0.051) | (0.052) |
| Chalet | -0.108 | -0.058 | -0.145* | -0.214*** | -0.110 |
|  | (0.094) | (0.078) | (0.075) | (0.075) | (0.076) |
| Entire | 0.270*** | 0.147*** | 0.135*** | 0.229*** | 0.110*** |
|  | (0.038) | (0.033) | (0.032) | (0.033) | (0.035) |
| Minimum stay | 0.009 | 0.010* | 0.008 | -0.000 | -0.005 |
|  | (0.006) | (0.005) | (0.005) | (0.005) | (0.006) |
| Bedrooms | -0.033*** | -0.008 | 0.005 | -0.012 | 0.004 |
|  | (0.012) | (0.010) | (0.010) | (0.010) | (0.010) |
| Number of photos | -0.002** | -0.004*** | -0.006*** | -0.006*** | -0.007*** |
|  | (0.001) | (0.001) | (0.001) | (0.001) | (0.001) |
| Never rated | -0.031 | -0.083** | -0.121*** | -0.171*** | -0.288*** |
|  | (0.038) | (0.033) | (0.033) | (0.034) | (0.036) |
| High rated | -0.093*** | -0.127*** | -0.143*** | -0.149*** | -0.193*** |
|  | (0.033) | (0.029) | (0.028) | (0.030) | (0.032) |
| Low rated | 0.121*** | 0.144*** | 0.132*** | 0.106*** | 0.044 |
|  | (0.042) | (0.038) | (0.038) | (0.040) | (0.044) |
| Canc. policy: Moderate | -0.067 | -0.081** | -0.066* | -0.091** | -0.059 |
|  | (0.045) | (0.039) | (0.038) | (0.039) | (0.041) |
| Canc. Policy: Strict | 0.028 | 0.014 | 0.075*** | 0.077*** | 0.095*** |
|  | (0.032) | (0.028) | (0.028) | (0.028) | (0.030) |
| Instant booking | -0.071** | -0.098*** | -0.137*** | -0.196*** | -0.170*** |
|  | (0.028) | (0.025) | (0.024) | (0.024) | (0.026) |
| Superhost | -0.157*** | -0.114*** | -0.155*** | -0.155*** | -0.123*** |
|  | (0.051) | (0.042) | (0.041) | (0.041) | (0.043) |
| Host experience | 2.6e-05 | -3.5e-05 | -9.3e-05*** | -9.9e-05*** | -1.3e-04*** |
|  | (3.0e-05) | (2.6e-05) | (2.6e-05) | (2.6e-05) | (2.8e-05) |
| Host number of listings | -1.6e-04 | -4.0e-04*** | -5.7e-04*** | -5.9e-04*** | -7.2e-04*** |
|  | (1.1e-04) | (1.0e-04) | (9.7e-05) | (9.7e-05) | (9.7e-05) |
| Constant | -1.089*** | -0.407*** | 0.014 | 0.333*** | 0.753*** |
|  | (0.062) | (0.053) | (0.051) | (0.052) | (0.055) |
| Observations | 16,663 | 16,663 | 16,663 | 16,663 | 16,663 |

**Table A8.** Propensity scores of probabilities of being located up to 500, 1000, 1500, 2000 and 2500 metres from the shoreline (probit regression). *** p<0.01, ** p<0.05, * p<0.1

Note: The reference categories are *Other, Shared/private, Medium rate* and *Flexible Canc.*

|  | (1) | (2) | (3) | (4) | (5) |
| --- | --- | --- | --- | --- | --- |
| Dependent variable: Ln ADR | Weighted by prop. Scores located within 500 m | Weighted by prop. Scores located within 1000 m | Weighted by prop. Scores located within 1500 m | Weighted by prop. Scores located within 2000 m | Weighted by prop. Scores located within 3000 m |
| Explanatory variables | Coeff. (SE) | Coeff. (SE) | Coeff. (SE) | Coeff. (SE) | Coeff. (SE) |
|  |  |  |  |  |  |
| Ln Distance | -0.008 | -0.006 | -0.006 | -0.006 | -0.005 |
|  | (0.007) | (0.007) | (0.007) | (0.007) | (0.007) |
| Ln Length | 0.033*** | 0.031*** | 0.031*** | 0.030*** | 0.030*** |
|  | (0.012) | (0.011) | (0.011) | (0.011) | (0.011) |
| Ln Width | -0.020 | -0.021 | -0.022 | -0.022 | -0.022* |
|  | (0.014) | (0.013) | (0.013) | (0.013) | (0.013) |
| Gold sand | -0.007 | -0.002 | -0.002 | -0.001 | 0.002 |
|  | (0.026) | (0.025) | (0.025) | (0.025) | (0.025) |
| Dark sand | -0.054 | -0.062 | -0.064 | -0.063 | -0.063 |
|  | (0.043) | (0.041) | (0.041) | (0.041) | (0.041) |
| Cliff front. | 0.090** | 0.098** | 0.100** | 0.101** | 0.104*** |
|  | (0.041) | (0.039) | (0.039) | (0.039) | (0.038) |
| Semi-urban front. | 0.128*** | 0.132*** | 0.132*** | 0.132*** | 0.133*** |
|  | (0.043) | (0.043) | (0.043) | (0.043) | (0.043) |
| Mountain front. | 0.120** | 0.122** | 0.124** | 0.124** | 0.124** |
|  | (0.053) | (0.053) | (0.053) | (0.052) | (0.052) |
| Dune front. | 0.145*** | 0.134*** | 0.136*** | 0.137*** | 0.131*** |
|  | (0.051) | (0.050) | (0.050) | (0.050) | (0.049) |
| Calm tide | -0.025 | -0.025 | -0.026 | -0.026 | -0.027 |
|  | (0.025) | (0.024) | (0.024) | (0.024) | (0.023) |
| Vegetation | 0.044* | 0.045* | 0.045* | 0.045* | 0.046* |
|  | (0.026) | (0.025) | (0.025) | (0.025) | (0.024) |
| Protec. area | -0.006 | -0.006 | -0.007 | -0.008 | -0.007 |
|  | (0.026) | (0.025) | (0.025) | (0.025) | (0.025) |
| Diff. access | 0.133*** | 0.121*** | 0.122*** | 0.120*** | 0.112*** |
|  | (0.045) | (0.044) | (0.044) | (0.043) | (0.043) |
| Only by boat | 0.004 | 0.015 | 0.016 | 0.017 | 0.023 |
|  | (0.063) | (0.060) | (0.060) | (0.059) | (0.058) |
| Isolated envir. | -0.082* | -0.082* | -0.083* | -0.082** | -0.082** |
|  | (0.043) | (0.042) | (0.042) | (0.042) | (0.041) |
| Semi-urban envir. | -0.079** | -0.081** | -0.082** | -0.082** | -0.084** |
|  | (0.038) | (0.037) | (0.037) | (0.036) | (0.036) |
| Municipality fixed effects | YES | YES | YES | YES | YES |
| Constant | 3.448*** | 3.683*** | 3.517*** | 3.572*** | 3.562*** |
|  | (0.201) | (0.153) | (0.122) | (0.078) | (0.071) |
| Observations | 16,663 | 16,663 | 16,663 | 16,663 | 16,663 |
| R-squared | 0.709 | 0.724 | 0.728 | 0.727 | 0.731 |

**Table A9.** WLS hedonic price regression estimates obtained by weighting observations by the propensity scores obtained from Table A8 (no interactions). Clustered standard errors at the beach level in parentheses. *** p<0.01, ** p<0.05, * p<0.1

Note: The reference categories are *Clear sand*, *Urban front*, *Easy Ac* and *Urban envir.*

|  | (1) | (2) | (3) | (4) | (5) |
| --- | --- | --- | --- | --- | --- |
| Dependent variable: Ln ADR | Weighted by prop. Scores located within 500 m | Weighted by prop. Scores located within 1000 m | Weighted by prop. Scores located within 1500 m | Weighted by prop. Scores located within 2000 m | Weighted by prop. Scores located within 3000 m |
| Explanatory variables | Coeff. (SE) | Coeff. (SE) | Coeff. (SE) | Coeff. (SE) | Coeff. (SE) |
|  |  |  |  |  |  |
| Ln Distance | 0.011 | 0.013 | 0.013 | 0.013 | 0.015 |
|  | (0.032) | (0.033) | (0.033) | (0.033) | (0.033) |
| Ln Length | 0.033*** | 0.032*** | 0.031*** | 0.031*** | 0.030*** |
|  | (0.011) | (0.011) | (0.011) | (0.011) | (0.010) |
| Ln Length x Ln distance | -0.007 | -0.007 | -0.007 | -0.007 | -0.007 |
|  | (0.005) | (0.005) | (0.005) | (0.005) | (0.005) |
| Ln Width | -0.015 | -0.016 | -0.016 | -0.016 | -0.015 |
|  | (0.014) | (0.014) | (0.014) | (0.014) | (0.013) |
| Ln Width x Ln distance | -0.013 | -0.012 | -0.012 | -0.013 | -0.012 |
|  | (0.008) | (0.008) | (0.008) | (0.008) | (0.008) |
| Gold sand | -0.007 | -0.004 | -0.004 | -0.003 | -0.001 |
|  | (0.025) | (0.024) | (0.024) | (0.024) | (0.024) |
| Gold sand x Ln Distance | 0.008 | 0.010 | 0.010 | 0.010 | 0.011 |
|  | (0.015) | (0.015) | (0.015) | (0.015) | (0.015) |
| Dark sand | -0.057 | -0.063 | -0.065* | -0.063* | -0.064* |
|  | (0.041) | (0.039) | (0.038) | (0.038) | (0.038) |
| Dark sand x Ln Distance | 0.008 | 0.007 | 0.007 | 0.007 | 0.008 |
|  | (0.023) | (0.022) | (0.022) | (0.022) | (0.022) |
| Cliff front. | 0.082* | 0.091** | 0.093** | 0.094** | 0.098** |
|  | (0.043) | (0.041) | (0.041) | (0.040) | (0.040) |
| Cliff front. x Ln Distance | 0.008 | 0.003 | 0.002 | 0.002 | -0.001 |
|  | (0.025) | (0.024) | (0.024) | (0.024) | (0.024) |
| Semi-urban front. | 0.134*** | 0.137*** | 0.137*** | 0.136*** | 0.136*** |
|  | (0.040) | (0.040) | (0.040) | (0.040) | (0.040) |
| Semi-urban front. x Ln Distance | 0.023 | 0.021 | 0.021 | 0.021 | 0.020 |
|  | (0.019) | (0.018) | (0.018) | (0.018) | (0.017) |
| Mountain front. | 0.152*** | 0.156*** | 0.157*** | 0.158*** | 0.159*** |
|  | (0.053) | (0.052) | (0.052) | (0.052) | (0.051) |
| Mountain front. x Ln Distance | -0.050* | -0.053* | -0.052* | -0.052* | -0.053* |
|  | (0.030) | (0.030) | (0.030) | (0.030) | (0.030) |
| Dune front. | 0.159*** | 0.146*** | 0.148*** | 0.149*** | 0.142*** |
|  | (0.056) | (0.055) | (0.055) | (0.055) | (0.054) |
| Dune front. x Ln Distance | -0.006 | -0.006 | -0.005 | -0.004 | -0.003 |
|  | (0.030) | (0.029) | (0.029) | (0.029) | (0.028) |
| Calm tide | -0.024 | -0.025 | -0.025 | -0.026 | -0.026 |
|  | (0.024) | (0.024) | (0.024) | (0.024) | (0.024) |
| Calm tide x Ln Distance | -0.001 | -0.003 | -0.003 | -0.003 | -0.004 |
|  | (0.012) | (0.012) | (0.012) | (0.012) | (0.012) |
| Vegetation | 0.038* | 0.038* | 0.039* | 0.039* | 0.039* |
|  | (0.022) | (0.022) | (0.022) | (0.022) | (0.022) |
| Vegetation x Ln Distance | 0.016 | 0.017 | 0.018 | 0.018 | 0.018 |
|  | (0.012) | (0.012) | (0.012) | (0.012) | (0.012) |
| Protec. area | 0.005 | 0.010 | 0.010 | 0.010 | 0.013 |
|  | (0.027) | (0.027) | (0.027) | (0.027) | (0.027) |
| Protect. area x Ln Distance | -0.040** | -0.040** | -0.040** | -0.040** | -0.040** |
|  | (0.017) | (0.017) | (0.017) | (0.017) | (0.017) |
| Diff. access | 0.200*** | 0.193*** | 0.193*** | 0.191*** | 0.186*** |
|  | (0.051) | (0.049) | (0.048) | (0.048) | (0.048) |
| Diff. access x Ln Distance | -0.081** | -0.084** | -0.084** | -0.084** | -0.086*** |
|  | (0.033) | (0.032) | (0.033) | (0.032) | (0.032) |
| Only by boat | -0.042 | -0.036 | -0.035 | -0.032 | -0.030 |
|  | (0.079) | (0.072) | (0.071) | (0.071) | (0.068) |
| Only by boat x Ln Distance | 0.015 | 0.020 | 0.021 | 0.020 | 0.024 |
|  | (0.044) | (0.041) | (0.041) | (0.041) | (0.040) |
| Isolated envir. | -0.108*** | -0.108*** | -0.108*** | -0.107*** | -0.107*** |
|  | (0.041) | (0.041) | (0.041) | (0.040) | (0.040) |
| Isolated envir. x Ln Distance | 0.044* | 0.041* | 0.039 | 0.038 | 0.036 |
|  | (0.024) | (0.025) | (0.025) | (0.025) | (0.025) |
| Semi-urban envir. | -0.075** | -0.079** | -0.080** | -0.080** | -0.083** |
|  | (0.036) | (0.035) | (0.035) | (0.035) | (0.034) |
| Semi-urban envir. x Ln Distance | 0.019 | 0.020 | 0.020 | 0.020 | 0.021 |
|  | (0.018) | (0.018) | (0.017) | (0.017) | (0.017) |
| Municipality fixed effects | YES | YES | YES | YES | YES |
| Constant | 3.611*** | 3.620*** | 3.622*** | 3.620*** | 3.614*** |
|  | (0.067) | (0.064) | (0.063) | (0.063) | (0.063) |
| Observations | 16,663 | 16,663 | 16,663 | 16,663 | 16,663 |
| R-squared | 0.711 | 0.726 | 0.729 | 0.728 | 0.733 |

**Table A10.** WLS hedonic price regression estimates obtained by weighting observations by the propensity scores obtained from Table A8 (with interactions). Clustered standard errors at the beach level in parentheses. *** p<0.01, ** p<0.05, * p<0.1

Note: The reference categories are *Clear sand*, *Urban front*, *Easy Ac* and *Urban envir.*

| Dependent variable: Ln ADR | (1) | (2) | (3) | (4) | (5) |
| --- | --- | --- | --- | --- | --- |
| Explanatory variables | Coeff. (SE) | Coeff. (SE) | Coeff. (SE) | Coeff. (SE) | Coeff. (SE) |
|  |  |  |  |  |  |
| Ln Distance | 0.002 | -0.068*** | -0.063*** | 0.001 | 0.029 |
|  | (0.018) | (0.017) | (0.016) | (0.007) | (0.039) |
| Ln Length | 0.017 | 0.032 | 0.030 | 0.027*** | 0.028*** |
|  | (0.031) | (0.027) | (0.025) | (0.010) | (0.009) |
| Ln Length x Ln distance | 0.000 |  |  |  | -0.007 |
|  |  |  |  |  | (0.006) |
| Ln Width | -0.055 | -0.085** | -0.080** | -0.016 | -0.008 |
|  | (0.044) | (0.037) | (0.034) | (0.013) | (0.013) |
| Ln Width x Ln distance |  |  |  |  | -0.015 |
|  |  |  |  |  | (0.009) |
| Gold sand | 0.106 | 0.259*** | 0.247*** | 0.014 | 0.010 |
|  | (0.089) | (0.070) | (0.065) | (0.025) | (0.025) |
| Gold sand x Ln Distance |  |  |  |  | 0.019 |
|  |  |  |  |  | (0.017) |
| Dark sand | -0.058 | 0.056 | 0.050 | -0.040 | -0.052 |
|  | (0.098) | (0.102) | (0.093) | (0.043) | (0.038) |
| Dark sand x Ln Distance |  |  |  |  | 0.021 |
|  |  |  |  |  | (0.023) |
| Cliff front. | 0.006 | 0.095 | 0.083 | 0.094*** | 0.092*** |
|  | (0.176) | (0.112) | (0.105) | (0.033) | (0.034) |
| Cliff front. x Ln Distance |  |  |  |  | -0.014 |
|  |  |  |  |  | (0.027) |
| Semi-urban front. | 0.328** | 0.175 | 0.164 | 0.116*** | 0.119*** |
|  | (0.149) | (0.117) | (0.110) | (0.038) | (0.036) |
| Semi-urban front. x Ln Distance |  |  |  |  | 0.008 |
|  |  |  |  |  | (0.017) |
| Mountain front. | 0.110 | 0.225* | 0.205* | 0.090* | 0.129*** |
|  | (0.172) | (0.124) | (0.116) | (0.046) | (0.046) |
| Mountain front. x Ln Distance |  |  |  |  | -0.059* |
|  |  |  |  |  | (0.030) |
| Dune front. | 0.154 | 0.136 | 0.122 | 0.112** | 0.103** |
|  | (0.188) | (0.153) | (0.143) | (0.044) | (0.046) |
| Dune front. x Ln Distance |  |  |  |  | 0.017 |
|  |  |  |  |  | (0.031) |
| Calm tide | 0.233* | 0.265*** | 0.247*** | -0.022 | -0.019 |
|  | (0.122) | (0.085) | (0.079) | (0.022) | (0.023) |
| Calm tide x Ln Distance |  |  |  |  | -0.006 |
|  |  |  |  |  | (0.013) |
| Vegetation | 0.134 | -0.001 | -0.004 | 0.051** | 0.045** |
|  | (0.091) | (0.073) | (0.068) | (0.022) | (0.020) |
| Vegetation x Ln Distance |  |  |  |  | 0.026* |
|  |  |  |  |  | (0.014) |
| Protec. area | -0.145 | -0.112 | -0.103 | -0.007 | 0.030 |
|  | (0.089) | (0.077) | (0.071) | (0.026) | (0.027) |
| Protect. area x Ln Distance |  |  |  |  | -0.037** |
|  |  |  |  |  | (0.017) |
| Diff. access | 0.119 | 0.051 | 0.063 | 0.113*** | 0.206*** |
|  | (0.134) | (0.097) | (0.090) | (0.043) | (0.041) |
| Diff. access x Ln Distance |  |  |  |  | -0.106*** |
|  |  |  |  |  | (0.027) |
| Only by boat | 0.139 | -0.155 | -0.144 | 0.094* | 0.054 |
|  | (0.155) | (0.117) | (0.108) | (0.055) | (0.057) |
| Only by boat x Ln Distance |  |  |  |  | 0.020 |
|  |  |  |  |  | (0.043) |
| Isolated envir. | 0.266* | -0.019 | -0.013 | -0.069* | -0.100*** |
|  | (0.148) | (0.104) | (0.097) | (0.039) | (0.037) |
| Isolated envir. x Ln Distance |  |  |  |  | 0.026 |
|  |  |  |  |  | (0.026) |
| Semi-urban envir. | 0.065 | -0.003 | 0.006 | -0.075** | -0.078** |
|  | (0.120) | (0.092) | (0.086) | (0.033) | (0.031) |
| Semi-urban envir. x Ln Distance |  |  |  |  | 0.019 |
|  |  |  |  |  | (0.018) |
| Structural characteristics | NO | YES | YES | YES | YES |
| Host characteristics | NO | NO | YES | YES | YES |
| Municipality fixed effects | NO | NO | NO | YES | YES |
| Constant | 4.924*** | 3.867*** | 3.844*** | 3.592*** | 3.569*** |
|  | (0.245) | (0.195) | (0.185) | (0.073) | (0.073) |
| Observations | 16,663 | 16,663 | 16,663 | 16,663 | 16,663 |
| R-squared | 0.075 | 0.643 | 0.658 | 0.746 | 0.747 |

**Table A11.** WLS hedonic price regression estimates under stepwise estimation. Clustered standard errors at the beach level in parentheses. *** p<0.01, ** p<0.05, * p<0.1

Note: The reference categories are *Clear sand*, *Urban front*, *Easy Ac* and *Urban envir.*

| Dependent variable: Ln ADR | (1) | (2) | (3) | (4) |
| --- | --- | --- | --- | --- |
|  | Default | Clustered at host level | Clustered at postal code level | Clustered at municipality level |
| Explanatory variables | Coeff. (SE) | Coeff. (SE) | Coeff. (SE) | Coeff. (SE) |
|  |  |  |  |  |
| Ln Distance | 0.029 | 0.029 | 0.029 | 0.029 |
|  | (0.022) | (0.026) | (0.034) | (0.035) |
| Ln Length | 0.028*** | 0.028*** | 0.028** | 0.028** |
|  | (0.005) | (0.006) | (0.011) | (0.013) |
| Ln Length x Ln distance | -0.007* | -0.007 | -0.007 | -0.007 |
|  | (0.004) | (0.004) | (0.005) | (0.006) |
| Ln Width | -0.008 | -0.008 | -0.008 | -0.008 |
|  | (0.007) | (0.009) | (0.014) | (0.015) |
| Ln Width x Ln distance | -0.015*** | -0.015** | -0.015* | -0.015* |
|  | (0.006) | (0.007) | (0.008) | (0.008) |
| Gold sand | 0.010 | 0.010 | 0.010 | 0.010 |
|  | (0.013) | (0.015) | (0.026) | (0.029) |
| Gold sand x Ln Distance | 0.019** | 0.019 | 0.019 | 0.019 |
|  | (0.010) | (0.011) | (0.016) | (0.016) |
| Dark sand | -0.052* | -0.052 | -0.052* | -0.052* |
|  | (0.030) | (0.033) | (0.030) | (0.031) |
| Dark sand x Ln Distance | 0.021 | 0.021 | 0.021 | 0.021 |
|  | (0.020) | (0.023) | (0.023) | (0.023) |
| Cliff front. | 0.092*** | 0.092*** | 0.092** | 0.092** |
|  | (0.021) | (0.024) | (0.037) | (0.041) |
| Cliff front. x Ln Distance | -0.014 | -0.014 | -0.014 | -0.014 |
|  | (0.016) | (0.019) | (0.028) | (0.038) |
| Semi-urban front. | 0.119*** | 0.119*** | 0.119*** | 0.119*** |
|  | (0.013) | (0.016) | (0.034) | (0.034) |
| Semi-urban front. x Ln Distance | 0.008 | 0.008 | 0.008 | 0.008 |
|  | (0.011) | (0.013) | (0.022) | (0.027) |
| Mountain front. | 0.129*** | 0.129*** | 0.129** | 0.129*** |
|  | (0.032) | (0.036) | (0.053) | (0.044) |
| Mountain front. x Ln Distance | -0.059** | -0.059** | -0.059* | -0.059 |
|  | (0.023) | (0.027) | (0.032) | (0.036) |
| Dune front. | 0.103*** | 0.103** | 0.103** | 0.103** |
|  | (0.033) | (0.041) | (0.046) | (0.046) |
| Dune front. x Ln Distance | 0.017 | 0.017 | 0.017 | 0.017 |
|  | (0.025) | (0.032) | (0.030) | (0.032) |
| Calm tide | -0.019 | -0.019 | -0.019 | -0.019 |
|  | (0.012) | (0.015) | (0.029) | (0.029) |
| Calm tide x Ln Distance | -0.006 | -0.006 | -0.006 | -0.006 |
|  | (0.008) | (0.010) | (0.011) | (0.009) |
| Vegetation | 0.045*** | 0.045*** | 0.045** | 0.045** |
|  | (0.012) | (0.014) | (0.019) | (0.020) |
| Vegetation x Ln Distance | 0.026*** | 0.026** | 0.026 | 0.026 |
|  | (0.009) | (0.011) | (0.017) | (0.021) |
| Protec. area | 0.030 | 0.030 | 0.030 | 0.030 |
|  | (0.021) | (0.023) | (0.027) | (0.027) |
| Protect. area x Ln Distance | -0.037*** | -0.037*** | -0.037** | -0.037 |
|  | (0.013) | (0.014) | (0.018) | (0.023) |
| Diff. access | 0.206*** | 0.206*** | 0.206*** | 0.206*** |
|  | (0.034) | (0.039) | (0.047) | (0.053) |
| Diff. access x Ln Distance | -0.106*** | -0.106*** | -0.106*** | -0.106*** |
|  | (0.024) | (0.029) | (0.031) | (0.039) |
| Only by boat | 0.054 | 0.054 | 0.054 | 0.054 |
|  | (0.069) | (0.077) | (0.064) | (0.068) |
| Only by boat x Ln Distance | 0.020 | 0.020 | 0.020 | 0.020 |
|  | (0.041) | (0.050) | (0.056) | (0.063) |
| Isolated envir. | -0.100*** | -0.100*** | -0.100** | -0.100** |
|  | (0.023) | (0.027) | (0.043) | (0.041) |
| Isolated envir. x Ln Distance | 0.026 | 0.026 | 0.026 | 0.026 |
|  | (0.018) | (0.020) | (0.026) | (0.031) |
| Semi-urban envir. | -0.078*** | -0.078*** | -0.078** | -0.078** |
|  | (0.016) | (0.018) | (0.031) | (0.032) |
| Semi-urban envir. x Ln Distance | 0.019 | 0.019 | 0.019 | 0.019 |
|  | (0.012) | (0.014) | (0.018) | (0.022) |
| Structural characteristics | YES | YES | YES | YES |
| Host characteristics | YES | YES | YES | YES |
| Municipality fixed effects | YES | YES | YES | YES |
| Constant | 3.569*** | 3.569*** | 3.569*** | 3.569*** |
|  | (0.076) | (0.087) | (0.073) | (0.080) |
| Observations | 16,663 | 16,663 | 16,663 | 16,663 |
| R-squared | 0.747 | 0.747 | 0.747 | 0.747 |

**Table A12.** WLS hedonic price regression estimates under different standard error clustering structures (in parentheses). *** p<0.01, ** p<0.05, * p<0.1

Note: The reference categories are *Clear sand*, *Urban front*, *Easy Ac* and *Urban envir.*

| Dependent variable: Ln ADR | (1) | (2) | (3) | (4) | (5) |
| --- | --- | --- | --- | --- | --- |
|  | Clustered at host and municipality level | Clustered at host and beach level | Clustered at beach and municipality level | Clustered at host and postal code level | Clustered at beach and postal code level |
| Explanatory variables | Coeff. (SE) | Coeff. (SE) | Coeff. (SE) | Coeff. (SE) | Coeff. (SE) |
|  |  |  |  |  |  |
| Ln Distance | 0.029 | 0.029 | 0.029 | 0.029 | 0.029 |
|  | (0.036) | (0.039) | (0.038) | (0.035) | (0.036) |
| Ln Length | 0.028** | 0.028*** | 0.028** | 0.028** | 0.028** |
|  | (0.014) | (0.010) | (0.014) | (0.011) | (0.011) |
| Ln Length x Ln distance | -0.007 | -0.007 | -0.007 | -0.007 | -0.007 |
|  | (0.006) | (0.006) | (0.006) | (0.006) | (0.005) |
| Ln Width | -0.008 | -0.008 | -0.008 | -0.008 | -0.008 |
|  | (0.016) | (0.013) | (0.016) | (0.014) | (0.014) |
| Ln Width x Ln distance | -0.015* | -0.015 | -0.015* | -0.015* | -0.015* |
|  | (0.008) | (0.010) | (0.009) | (0.008) | (0.009) |
| Gold sand | 0.010 | 0.010 | 0.010 | 0.010 | 0.010 |
|  | (0.030) | (0.025) | (0.031) | (0.026) | (0.026) |
| Gold sand x Ln Distance | 0.019 | 0.019 | 0.019 | 0.019 | 0.019 |
|  | (0.017) | (0.018) | (0.018) | (0.017) | (0.018) |
| Dark sand | -0.052 | -0.052 | -0.052 | -0.052* | -0.052* |
|  | (0.032) | (0.039) | (0.033) | (0.031) | (0.031) |
| Dark sand x Ln Distance | 0.021 | 0.021 | 0.021 | 0.021 | 0.021 |
|  | (0.025) | (0.024) | (0.024) | (0.024) | (0.023) |
| Cliff front. | 0.092** | 0.092*** | 0.092** | 0.092** | 0.092*** |
|  | (0.042) | (0.034) | (0.042) | (0.038) | (0.034) |
| Cliff front. x Ln Distance | -0.014 | -0.014 | -0.014 | -0.014 | -0.014 |
|  | (0.039) | (0.027) | (0.039) | (0.029) | (0.030) |
| Semi-urban front. | 0.119*** | 0.119*** | 0.119*** | 0.119*** | 0.119*** |
|  | (0.035) | (0.036) | (0.038) | (0.036) | (0.038) |
| Semi-urban front. x Ln Distance | 0.008 | 0.008 | 0.008 | 0.008 | 0.008 |
|  | (0.028) | (0.017) | (0.026) | (0.022) | (0.022) |
| Mountain front. | 0.129*** | 0.129*** | 0.129*** | 0.129** | 0.129** |
|  | (0.045) | (0.046) | (0.046) | (0.053) | (0.054) |
| Mountain front. x Ln Distance | -0.059 | -0.059* | -0.059 | -0.059* | -0.059* |
|  | (0.037) | (0.031) | (0.036) | (0.033) | (0.032) |
| Dune front. | 0.103** | 0.103** | 0.103** | 0.103** | 0.103** |
|  | (0.047) | (0.045) | (0.048) | (0.046) | (0.044) |
| Dune front. x Ln Distance | 0.017 | 0.017 | 0.017 | 0.017 | 0.017 |
|  | (0.033) | (0.031) | (0.031) | (0.031) | (0.030) |
| Calm tide | -0.019 | -0.019 | -0.019 | -0.019 | -0.019 |
|  | (0.030) | (0.023) | (0.029) | (0.029) | (0.028) |
| Calm tide x Ln Distance | -0.006 | -0.006 | -0.006 | -0.006 | -0.006 |
|  | (0.010) | (0.015) | (0.011) | (0.012) | (0.012) |
| Vegetation | 0.045** | 0.045** | 0.045** | 0.045** | 0.045** |
|  | (0.020) | (0.021) | (0.020) | (0.019) | (0.019) |
| Vegetation x Ln Distance | 0.026 | 0.026* | 0.026 | 0.026 | 0.026 |
|  | (0.022) | (0.015) | (0.022) | (0.018) | (0.018) |
| Protec. area | 0.030 | 0.030 | 0.030 | 0.030 | 0.030 |
|  | (0.028) | (0.027) | (0.027) | (0.028) | (0.028) |
| Protect. area x Ln Distance | -0.037 | -0.037** | -0.037 | -0.037** | -0.037** |
|  | (0.023) | (0.018) | (0.023) | (0.018) | (0.018) |
| Diff. access | 0.206*** | 0.206*** | 0.206*** | 0.206*** | 0.206*** |
|  | (0.054) | (0.042) | (0.054) | (0.048) | (0.047) |
| Diff. access x Ln Distance | -0.106** | -0.106*** | -0.106*** | -0.106*** | -0.106*** |
|  | (0.040) | (0.028) | (0.039) | (0.032) | (0.031) |
| Only by boat | 0.054 | 0.054 | 0.054 | 0.054 | 0.054 |
|  | (0.070) | (0.059) | (0.064) | (0.066) | (0.058) |
| Only by boat x Ln Distance | 0.020 | 0.020 | 0.020 | 0.020 | 0.020 |
|  | (0.065) | (0.046) | (0.058) | (0.060) | (0.052) |
| Isolated envir. | -0.100** | -0.100*** | -0.100** | -0.100** | -0.100** |
|  | (0.041) | (0.037) | (0.043) | (0.043) | (0.044) |
| Isolated envir. x Ln Distance | 0.026 | 0.026 | 0.026 | 0.026 | 0.026 |
|  | (0.031) | (0.026) | (0.030) | (0.027) | (0.027) |
| Semi-urban envir. | -0.078** | -0.078** | -0.078** | -0.078** | -0.078** |
|  | (0.032) | (0.031) | (0.033) | (0.032) | (0.033) |
| Semi-urban envir. x Ln Distance | 0.019 | 0.019 | 0.019 | 0.019 | 0.019 |
|  | (0.023) | (0.018) | (0.022) | (0.019) | (0.019) |
| Structural characteristics | YES | YES | YES | YES | YES |
| Host characteristics | YES | YES | YES | YES | YES |
| Municipality fixed effects | YES | YES | YES | YES | YES |
| Constant | 3.569*** | 3.569*** | 3.569*** | 3.569*** | 3.569*** |
|  | (0.078) | (0.071) | (0.085) | (0.071) | (0.077) |
| Observations | 16,663 | 16,663 | 16,663 | 16,663 | 16,663 |
| R-squared | 0.747 | 0.747 | 0.747 | 0.747 | 0.747 |

**Table A13.** WLS hedonic price regression estimates considering different two-way standard error clustering (in parentheses). *** p<0.01, ** p<0.05, * p<0.1

Note: The reference categories are *Clear sand*, *Urban front*, *Easy Ac* and *Urban envir*

| Dependent variable: Ln ADR | (1) | (2) | (3) | (4) |
| --- | --- | --- | --- | --- |
|  | Dist. Cutoff=250 m | Dist. Cutoff=500 m | Dist. Cutoff=750 m | Dist. Cutoff=1000 m |
| Explanatory variables | Coeff. (SE) | Coeff. (SE) | Coeff. (SE) | Coeff. (SE) |
|  |  |  |  |  |
| Ln Distance | 0.029 | 0.029 | 0.029 | 0.029 |
|  | (0.032) | (0.031) | (0.029) | (0.026) |
| Ln Length | 0.028*** | 0.028*** | 0.028*** | 0.028*** |
|  | (0.009) | (0.009) | (0.008) | (0.007) |
| Ln Length x Ln distance | -0.007 | -0.007 | -0.007 | -0.007 |
|  | (0.006) | (0.006) | (0.005) | (0.005) |
| Ln Width | -0.008 | -0.008 | -0.008 | -0.008 |
|  | (0.012) | (0.011) | (0.010) | (0.009) |
| Ln Width x Ln distance | -0.015* | -0.015** | -0.015** | -0.015** |
|  | (0.008) | (0.007) | (0.007) | (0.006) |
| Gold sand | 0.010 | 0.010 | 0.010 | 0.010 |
|  | (0.021) | (0.020) | (0.019) | (0.016) |
| Gold sand x Ln Distance | 0.019 | 0.019 | 0.019 | 0.019* |
|  | (0.014) | (0.013) | (0.013) | (0.011) |
| Dark sand | -0.052 | -0.052 | -0.052 | -0.052* |
|  | (0.032) | (0.032) | (0.032) | (0.031) |
| Dark sand x Ln Distance | 0.021 | 0.021 | 0.021 | 0.021 |
|  | (0.026) | (0.025) | (0.024) | (0.022) |
| Cliff front. | 0.092** | 0.092*** | 0.092*** | 0.092*** |
|  | (0.036) | (0.034) | (0.031) | (0.026) |
| Cliff front. x Ln Distance | -0.014 | -0.014 | -0.014 | -0.014 |
|  | (0.025) | (0.024) | (0.022) | (0.019) |
| Semi-urban front. | 0.119*** | 0.119*** | 0.119*** | 0.119*** |
|  | (0.031) | (0.030) | (0.027) | (0.021) |
| Semi-urban front. x Ln Distance | 0.008 | 0.008 | 0.008 | 0.008 |
|  | (0.016) | (0.016) | (0.015) | (0.013) |
| Mountain front. | 0.129*** | 0.129*** | 0.129*** | 0.129*** |
|  | (0.045) | (0.043) | (0.040) | (0.036) |
| Mountain front. x Ln Distance | -0.059** | -0.059** | -0.059** | -0.059** |
|  | (0.030) | (0.029) | (0.028) | (0.026) |
| Dune front. | 0.103* | 0.103** | 0.103** | 0.103** |
|  | (0.053) | (0.051) | (0.048) | (0.041) |
| Dune front. x Ln Distance | 0.017 | 0.017 | 0.017 | 0.017 |
|  | (0.037) | (0.037) | (0.035) | (0.031) |
| Calm tide | -0.019 | -0.019 | -0.019 | -0.019 |
|  | (0.023) | (0.022) | (0.020) | (0.016) |
| Calm tide x Ln Distance | -0.006 | -0.006 | -0.006 | -0.006 |
|  | (0.012) | (0.012) | (0.011) | (0.010) |
| Vegetation | 0.045** | 0.045** | 0.045*** | 0.045*** |
|  | (0.019) | (0.019) | (0.017) | (0.015) |
| Vegetation x Ln Distance | 0.026* | 0.026** | 0.026** | 0.026** |
|  | (0.013) | (0.013) | (0.012) | (0.011) |
| Protec. area | 0.030 | 0.030 | 0.030 | 0.030 |
|  | (0.027) | (0.026) | (0.025) | (0.023) |
| Protect. area x Ln Distance | -0.037** | -0.037** | -0.037** | -0.037** |
|  | (0.016) | (0.016) | (0.016) | (0.014) |
| Diff. access | 0.206*** | 0.206*** | 0.206*** | 0.206*** |
|  | (0.044) | (0.043) | (0.042) | (0.039) |
| Diff. access x Ln Distance | -0.106*** | -0.106*** | -0.106*** | -0.106*** |
|  | (0.031) | (0.030) | (0.029) | (0.027) |
| Only by boat | 0.054 | 0.054 | 0.054 | 0.054 |
|  | (0.082) | (0.082) | (0.080) | (0.076) |
| Only by boat x Ln Distance | 0.020 | 0.020 | 0.020 | 0.020 |
|  | (0.053) | (0.052) | (0.050) | (0.046) |
| Isolated envir. | -0.100*** | -0.100*** | -0.100*** | -0.100*** |
|  | (0.035) | (0.034) | (0.031) | (0.027) |
| Isolated envir. x Ln Distance | 0.026 | 0.026 | 0.026 | 0.026 |
|  | (0.025) | (0.024) | (0.022) | (0.020) |
| Semi-urban envir. | -0.078*** | -0.078*** | -0.078*** | -0.078*** |
|  | (0.025) | (0.023) | (0.022) | (0.019) |
| Semi-urban envir. x Ln Distance | 0.019 | 0.019 | 0.019 | 0.019 |
|  | (0.016) | (0.015) | (0.014) | (0.013) |
| Structural characteristics | YES | YES | YES | YES |
| Host characteristics | YES | YES | YES | YES |
| Municipality fixed effects | YES | YES | YES | YES |
| Constant | 3.569*** | 3.569*** | 3.569*** | 3.569*** |
|  | (0.084) | (0.083) | (0.085) | (0.083) |
| Observations | 16,663 | 16,663 | 16,663 | 16,663 |
| R-squared | 0.747 | 0.747 | 0.747 | 0.747 |

**Table A14.** WLS hedonic price regression estimates under arbitrary standard error clustering (Conley, 1999) with different distance cutoffs. *** p<0.01, ** p<0.05, * p<0.1

Note: The reference categories are *Clear sand*, *Urban front*, *Easy Ac* and *Urban envir.*

| Dependent variable: Ln ADR | (1) | (3) |
| --- | --- | --- |
| Explanatory variables | Coeff. (SE) | Coeff. (SE) |
|  |  |  |
| Ln Closeness | -0.001 | -0.048 |
|  | (0.007) | (0.046) |
| Ln Length | 0.027*** | 0.028*** |
|  | (0.010) | (0.009) |
| Ln Length x Ln Closeness |  | 0.007 |
|  |  | (0.006) |
| Ln Width | -0.016 | -0.008 |
|  | (0.013) | (0.013) |
| Ln Width x Ln Closeness |  | 0.015 |
|  |  | (0.009) |
| Gold sand | 0.014 | 0.010 |
|  | (0.025) | (0.025) |
| Gold sand x Ln Closeness |  | -0.019 |
|  |  | (0.017) |
| Dark sand | -0.040 | -0.052 |
|  | (0.043) | (0.038) |
| Dark sand x Ln Closeness |  | -0.021 |
|  |  | (0.023) |
| Cliff front. | 0.094*** | 0.092*** |
|  | (0.033) | (0.034) |
| Cliff front. x Ln Closeness |  | 0.014 |
|  |  | (0.027) |
| Semi-urban front. | 0.116*** | 0.119*** |
|  | (0.038) | (0.036) |
| Semi-urban front. x Ln Closeness |  | -0.008 |
|  |  | (0.017) |
| Mountain front. | 0.090* | 0.129*** |
|  | (0.046) | (0.046) |
| Mountain front. x Ln Closeness |  | 0.059* |
|  |  | (0.030) |
| Dune front. | 0.112** | 0.103** |
|  | (0.044) | (0.046) |
| Dune front. x Ln Closeness |  | -0.017 |
|  |  | (0.031) |
| Calm tide | -0.022 | -0.019 |
|  | (0.022) | (0.023) |
| Calm tide x Ln Closeness |  | 0.006 |
|  |  | (0.013) |
| Vegetation | 0.051** | 0.045** |
|  | (0.022) | (0.020) |
| Vegetation x Ln Closeness |  | -0.026* |
|  |  | (0.014) |
| Protec. area | -0.007 | 0.030 |
|  | (0.026) | (0.027) |
| Protect. area x Ln Closeness |  | 0.037** |
|  |  | (0.017) |
| Diff. access | 0.113*** | 0.206*** |
|  | (0.043) | (0.041) |
| Diff. access x Ln Closeness |  | 0.106*** |
|  |  | (0.027) |
| Only by boat | 0.094* | 0.054 |
|  | (0.055) | (0.057) |
| Only by boat x Ln Closeness |  | -0.020 |
|  |  | (0.043) |
| Isolated envir. | -0.069* | -0.100*** |
|  | (0.039) | (0.037) |
| Isolated envir. x Ln Closeness |  | -0.007 |
|  |  | (0.021) |
| Semi-urban envir. | -0.075** | -0.078** |
|  | (0.033) | (0.031) |
| Semi-urban envir. x Ln Closeness |  | 0.019 |
|  |  | (0.018) |
| Structural characteristics | YES | YES |
| Host characteristics | YES | YES |
| Municipality fixed effects | YES | YES |
| Constant | 3.592*** | 3.569*** |
|  | (0.073) | (0.073) |
| Observations | 16,663 | 16,663 |
| R-squared | 0.746 | 0.747 |

**Table A15.** WLS hedonic price regression estimates using closeness (i.e., 1/distance) instead of distance. Clustered standard errors at the beach level in parentheses. *** p<0.01, ** p<0.05, * p<0.1

Note: The reference categories are *Clear sand*, *Urban front*, *Easy Ac* and *Urban envir.*

| Dependent variable: Ln ADR | (1) | (2) | (3) | (4) |
| --- | --- | --- | --- | --- |
|  | Entire | Entire | Shared/private | Shared/private |
| Explanatory variables | Coeff. (SE) | Coeff. (SE) | Coeff. (SE) | Coeff. (SE) |
|  |  |  |  |  |
| Ln Distance | -0.010 | 0.024 | 0.025 | 0.040 |
|  | (0.007) | (0.036) | (0.017) | (0.108) |
| Ln Length | 0.033*** | 0.032*** | 0.025 | 0.025 |
|  | (0.010) | (0.010) | (0.021) | (0.020) |
| Ln Length x Ln distance |  | -0.008 |  | -0.001 |
|  |  | (0.005) |  | (0.017) |
| Ln Width | -0.019 | -0.010 | 0.002 | 0.004 |
|  | (0.014) | (0.014) | (0.030) | (0.027) |
| Ln Width x Ln distance |  | -0.014* |  | -0.015 |
|  |  | (0.008) |  | (0.028) |
| Gold sand | 0.009 | 0.011 | 0.061 | 0.039 |
|  | (0.024) | (0.024) | (0.057) | (0.057) |
| Gold sand x Ln Distance |  | 0.008 |  | 0.027 |
|  |  | (0.016) |  | (0.040) |
| Dark sand | -0.042 | -0.061 | -0.102 | -0.090 |
|  | (0.047) | (0.042) | (0.073) | (0.074) |
| Dark sand x Ln Distance |  | 0.022 |  | -0.026 |
|  |  | (0.023) |  | (0.072) |
| Cliff front. | 0.059* | 0.055 | 0.198*** | 0.191*** |
|  | (0.035) | (0.036) | (0.073) | (0.073) |
| Cliff front. x Ln Distance |  | -0.017 |  | -0.012 |
|  |  | (0.028) |  | (0.046) |
| Semi-urban front. | 0.112*** | 0.116*** | 0.095 | 0.089 |
|  | (0.037) | (0.035) | (0.066) | (0.073) |
| Semi-urban front. x Ln Distance |  | 0.005 |  | 0.012 |
|  |  | (0.019) |  | (0.044) |
| Mountain front. | 0.088* | 0.139*** | -0.025 | -0.046 |
|  | (0.045) | (0.051) | (0.113) | (0.095) |
| Mountain front. x Ln Distance |  | -0.084*** |  | 0.024 |
|  |  | (0.029) |  | (0.079) |
| Dune front. | 0.109** | 0.079 | 0.023 | 0.114 |
|  | (0.047) | (0.050) | (0.109) | (0.114) |
| Dune front. x Ln Distance |  | 0.034 |  | -0.106 |
|  |  | (0.029) |  | (0.099) |
| Calm tide | -0.021 | -0.018 | -0.031 | -0.025 |
|  | (0.022) | (0.023) | (0.047) | (0.048) |
| Calm tide x Ln Distance |  | -0.004 |  | 0.006 |
|  |  | (0.012) |  | (0.035) |
| Vegetation | 0.036 | 0.030 | 0.115** | 0.101** |
|  | (0.024) | (0.022) | (0.053) | (0.050) |
| Vegetation x Ln Distance |  | 0.016 |  | 0.049 |
|  |  | (0.014) |  | (0.038) |
| Protec. area | -0.015 | 0.026 | 0.065 | 0.096 |
|  | (0.026) | (0.028) | (0.071) | (0.074) |
| Protect. area x Ln Distance |  | -0.039** |  | -0.029 |
|  |  | (0.018) |  | (0.054) |
| Diff. access | 0.128*** | 0.201*** | 0.163 | 0.256** |
|  | (0.043) | (0.049) | (0.120) | (0.099) |
| Diff. access x Ln Distance |  | -0.081*** |  | -0.128 |
|  |  | (0.028) |  | (0.095) |
| Only by boat | 0.050 | 0.046 | 0.319** | 0.088 |
|  | (0.053) | (0.061) | (0.135) | (0.148) |
| Only by boat x Ln Distance |  | -0.010 |  | 0.167 |
|  |  | (0.042) |  | (0.114) |
| Isolated envir. | -0.054 | -0.092** | -0.014 | -0.012 |
|  | (0.041) | (0.041) | (0.089) | (0.091) |
| Isolated envir. x Ln Distance |  | 0.038 |  | -0.016 |
|  |  | (0.026) |  | (0.068) |
| Semi-urban envir. | -0.062* | -0.066* | -0.017 | -0.036 |
|  | (0.036) | (0.034) | (0.073) | (0.073) |
| Semi-urban envir. x Ln Distance |  | 0.014 |  | 0.029 |
|  |  | (0.018) |  | (0.054) |
| Structural characteristics | YES | YES | YES | YES |
| Host characteristics | YES | YES | YES | YES |
| Municipality fixed effects | YES | YES | YES | YES |
| Constant | 4.113*** | 4.097*** | 4.120*** | 4.150*** |
|  | (0.079) | (0.095) | (0.147) | (0.172) |
| Observations | 13,887 | 13,887 | 2,776 | 2,776 |
| R-squared | 0.695 | 0.697 | 0.497 | 0.502 |

**Table A16.** Separate WLS hedonic price regression estimates by type of property. Clustered standard errors at the beach level in parentheses. *** p<0.01, ** p<0.05, * p<0.1

Note: The reference categories are *Clear sand*, *Urban front*, *Easy Ac* and *Urban envir*


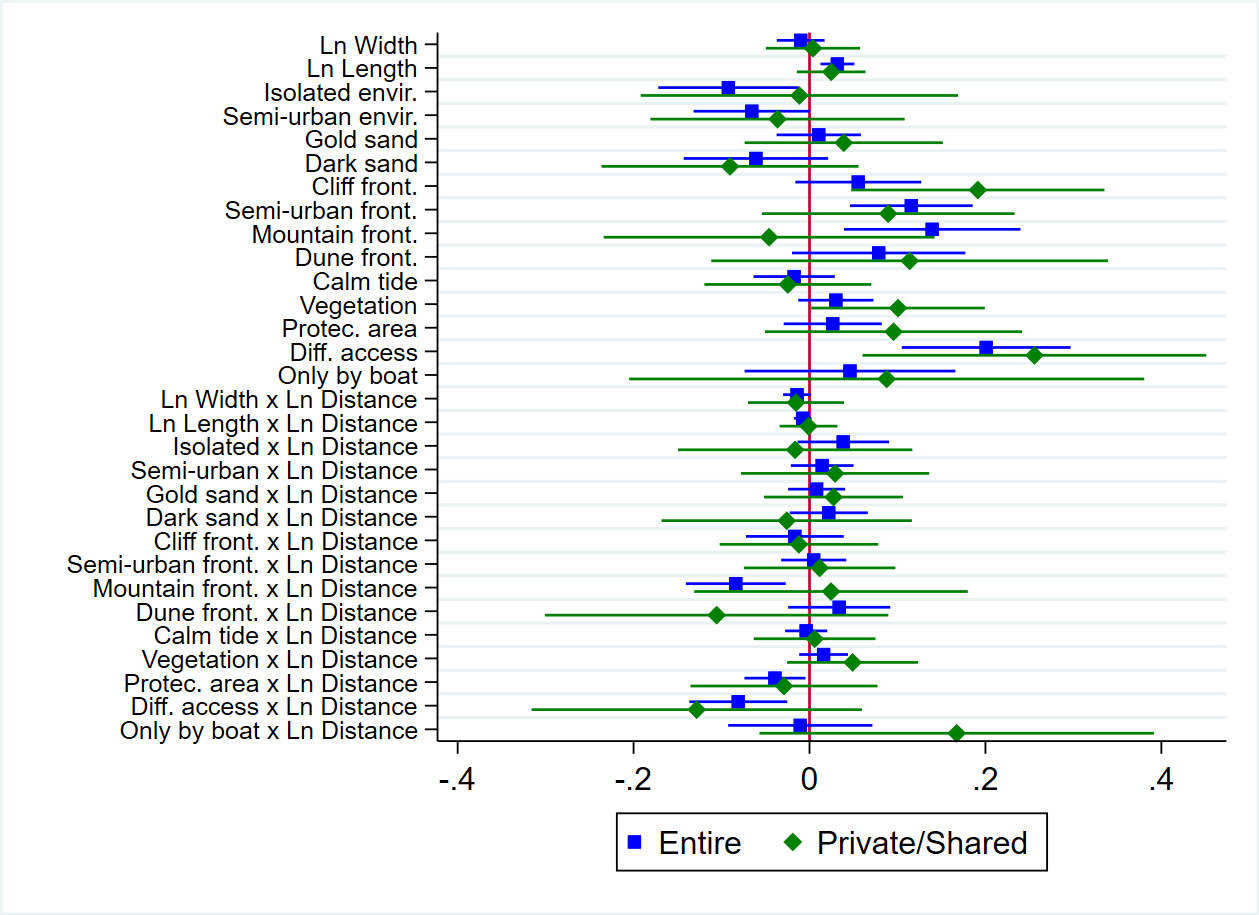


**Figure A12.** Coefficient estimates for separate regressions per island

|  | (1) |
| --- | --- |
|  | Entire-Shared/private |
| Explanatory variables | Chi2(1) [pvalue] |
|  |  |
| Ln Distance | 0.03 |
|  | [0.861] |
| Ln Length | 0.01 |
|  | [0.975] |
| Ln Length x Ln distance | 0.02 |
|  | [0.880] |
| Ln Width | 0.02 |
|  | [0.887] |
| Ln Width x Ln distance | 0.38 |
|  | [0.536] |
| Gold sand | 0.05 |
|  | [0.827] |
| Gold sand x Ln Distance | 0.60 |
|  | [0.439] |
| Dark sand | 0.28 |
|  | [0.595] |
| Dark sand x Ln Distance | 0.95 |
|  | [0.330] |
| Cliff front. | 2.88 |
|  | [0.089] |
| Cliff front. x Ln Distance | 0.08 |
|  | [0.782] |
| Semi-urban front. | 0.07 |
|  | [0.797] |
| Semi-urban front. x Ln Distance | 0.43 |
|  | [0.510] |
| Mountain front. | 4.42 |
|  | [0.035] |
| Mountain front. x Ln Distance | 3.25 |
|  | [0.071] |
| Dune front. | 0.49 |
|  | [0.482] |
| Dune front. x Ln Distance | 2.25 |
|  | [0.134] |
| Calm tide | 0.16 |
|  | [0.693] |
| Calm tide x Ln Distance | 0.01 |
|  | [0.970] |
| Vegetation | 3.89 |
|  | [0.048] |
| Vegetation x Ln Distance | 1.18 |
|  | [0.277] |
| Protec. area | 0.30 |
|  | [0.581] |
| Protect. area x Ln Distance | 0.05 |
|  | [0.831] |
| Diff. access | 0.22 |
|  | [0.635] |
| Diff. access x Ln Distance | 1.24 |
|  | [0.265] |
| Only by boat | 0.01 |
|  | [0.925] |
| Only by boat x Ln Distance | 4.24 |
|  | [0.039] |
| Isolated envir. | 0.61 |
|  | [0.434] |
| Isolated envir. x Ln Distance | 0.63 |
|  | [0.427] |
| Semi-urban envir. | 0.59 |
|  | [0.440] |
| Semi-urban envir. x Ln Distance | 0.02 |
|  | [0.889] |
| All | 64737.87 |
|  | [<0.001] |

**Table A17.** Pairwise coefficient comparison (chi squared test) from separate regressions by type of property (Table A16)

| Dependent variable: Ln ADR | (1) | (2) | (3) | (4) | (5) | (6) | (7) | (8) |
| --- | --- | --- | --- | --- | --- | --- | --- | --- |
|  | Mallorca (All) | Mallorca (All) | Mallorca (Less 1,000 metres) | Ibiza and Formentera (All) | Ibiza and Formentera (All) | Ibiza and Formentera (Less 1,000 metres) | Menorca (All) | Menorca (All) |
| Explanatory variables | Coeff. (SE) | Coeff. (SE) |  | Coeff. (SE) | Coeff. (SE) |  |  |  |
|  |  |  |  |  |  |  |  |  |
| Ln Distance | 2.4e-04 | 0.037 | 0.128 | 0.009 | -0.020 | -0.012 | 0.019 | 0.255 |
|  | (0.008) | (0.044) | (0.084) | (0.014) | (0.067) | (0.076) | (0.040) | (0.386) |
| Ln Length | 0.007 | 0.005 | -0.001 | 0.046*** | 0.039*** | 0.038*** | 0.161*** | 0.128*** |
|  | (0.009) | (0.009) | (0.014) | (0.014) | (0.012) | (0.014) | (0.044) | (0.045) |
| Ln Length x Ln distance |  | -0.003 | -0.012 |  | -0.023* | -0.023* |  | 0.004 |
|  |  | (0.005) | (0.012) |  | (0.012) | (0.013) |  | (0.034) |
| Ln Width | -0.027* | -0.020 | -0.041 | 0.003 | 0.009 | 0.005 | 0.134 | 0.210* |
|  | (0.016) | (0.016) | (0.028) | (0.015) | (0.013) | (0.015) | (0.110) | (0.118) |
| Ln Width x Ln distance |  | -0.010 | -0.049** |  | -0.010 | -0.015 |  | -0.150 |
|  |  | (0.011) | (0.022) |  | (0.016) | (0.017) |  | (0.130) |
| Gold sand | -0.030 | -0.029 | -0.073 | 0.035 | 0.038 | 0.041 | 0.067 | 0.077 |
|  | (0.026) | (0.028) | (0.056) | (0.031) | (0.031) | (0.033) | (0.095) | (0.053) |
| Gold sand x Ln Distance |  | -0.006 | -0.056 |  | 0.019 | 0.023 |  | 0.140 |
|  |  | (0.026) | (0.039) |  | (0.024) | (0.026) |  | (0.121) |
| Dark sand | -0.046 | -0.095* | -0.111 | -0.026 | -0.066 | -0.066 | -0.058 | 0.005 |
|  | (0.057) | (0.051) | (0.114) | (0.050) | (0.047) | (0.068) | (0.283) | (0.307) |
| Dark sand x Ln Distance |  | 0.048 | 0.036 |  | 0.058 | 0.131* |  | -0.165 |
|  |  | (0.033) | (0.089) |  | (0.064) | (0.075) |  | (0.188) |
| Cliff front. | 0.013 | 0.007 | 0.017 | 0.185*** | 0.201*** | 0.204*** | -0.384** | -0.521*** |
|  | (0.032) | (0.029) | (0.057) | (0.062) | (0.056) | (0.067) | (0.144) | (0.147) |
| Cliff front. x Ln Distance |  | -0.020 | 0.037 |  | -0.153** | -0.170*** |  | -0.215 |
|  |  | (0.028) | (0.045) |  | (0.059) | (0.061) |  | (0.150) |
| Semi-urban front. | 0.055* | 0.062** | -0.034 | 0.130*** | 0.141*** | 0.139*** | -0.178 | -0.129 |
|  | (0.030) | (0.027) | (0.057) | (0.040) | (0.034) | (0.035) | (0.141) | (0.122) |
| Semi-urban front. x Ln Distance |  | -0.004 | -0.061 |  | 0.019 | 0.021 |  | -0.219* |
|  |  | (0.020) | (0.048) |  | (0.020) | (0.020) |  | (0.112) |
| Mountain front. | -0.060 | 0.048 | 0.176 | 0.245*** | 0.218*** | 0.230*** | 0.401* | 0.523** |
|  | (0.050) | (0.063) | (0.146) | (0.069) | (0.057) | (0.063) | (0.227) | (0.231) |
| Mountain front. x Ln Distance |  | -0.089** | 0.013 |  | -0.101* | -0.119** |  | -0.273 |
|  |  | (0.035) | (0.078) |  | (0.052) | (0.053) |  | (0.268) |
| Dune front. | 0.033 | -2.6e-04 | -0.101 | 0.212*** | 0.201*** | 0.223** | -0.261 | -0.163 |
|  | (0.047) | (0.045) | (0.135) | (0.062) | (0.066) | (0.096) | (0.193) | (0.155) |
| Dune front. x Ln Distance |  | 0.049 | -0.193 |  | -0.033 | -0.021 |  | -0.696*** |
|  |  | (0.035) | (0.169) |  | (0.080) | (0.094) |  | (0.200) |
| Calm tide | -0.012 | -0.003 | -0.009 | -0.039 | -0.022 | -0.017 | -0.150 | 0.012 |
|  | (0.023) | (0.023) | (0.046) | (0.032) | (0.033) | (0.038) | (0.212) | (0.213) |
| Calm tide x Ln Distance |  | -0.020 | -0.017 |  | 0.021 | 0.024 |  | 0.176* |
|  |  | (0.015) | (0.035) |  | (0.038) | (0.044) |  | (0.092) |
| Vegetation | 0.033 | 0.022 | 0.035 | 0.049* | 0.049* | 0.054* | -0.172* | -0.261** |
|  | (0.023) | (0.023) | (0.046) | (0.027) | (0.026) | (0.027) | (0.090) | (0.101) |
| Vegetation x Ln Distance |  | 0.028* | 0.032 |  | 0.025 | 0.034 |  | 0.043 |
|  |  | (0.016) | (0.031) |  | (0.027) | (0.028) |  | (0.056) |
| Protec. area | -0.013 | 0.025 | -0.008 | -0.018 | 0.041 | 0.008 | -0.186 | -0.043 |
|  | (0.029) | (0.030) | (0.072) | (0.072) | (0.063) | (0.074) | (0.131) | (0.108) |
| Protect. area x Ln Distance |  | -0.023 | 0.018 |  | 0.004 | 0.045 |  | 0.497*** |
|  |  | (0.019) | (0.055) |  | (0.041) | (0.052) |  | (0.115) |
| Diff. access | 0.063 | 0.111** | 0.091 | 0.225*** | 0.276*** | 0.240*** | 0.759** | 2.249* |
|  | (0.046) | (0.048) | (0.193) | (0.052) | (0.048) | (0.059) | (0.356) | (1.260) |
| Diff. access x Ln Distance |  | -0.034 | -0.061 |  | -0.162*** | -0.155*** |  | -0.730 |
|  |  | (0.035) | (0.116) |  | (0.037) | (0.045) |  | (0.799) |
| Only by boat | 0.109* | -1.6e-04 | -0.244 | 0.014 | 0.206* | 0.166 | - | - |
|  | (0.064) | (0.084) | (0.182) | (0.066) | (0.120) | (0.116) |  |  |
| Only by boat x Ln Distance |  | -0.012 | -0.836*** |  | -0.213 | -0.137 |  | - |
|  |  | (0.028) | (0.095) |  | (0.133) | (0.102) |  |  |
| Isolated envir. | 0.025 | 0.019 | 0.006 | -0.177*** | -0.209*** | -0.204*** | -1.071*** | 2.428** |
|  | (0.046) | (0.041) | (0.109) | (0.049) | (0.038) | (0.043) | (0.336) | (0.925) |
| Isolated envir. x Ln Distance |  | 0.015 | -0.078 |  | 0.099*** | 0.095** |  | -2.472*** |
|  |  | (0.018) | (0.099) |  | (0.036) | (0.038) |  | (0.636) |
| Semi-urban envir. | 0.020 | 0.006 | 0.064 | -0.149*** | -0.165*** | -0.163*** | 0.001 | 0.061 |
|  | (0.033) | (0.031) | (0.046) | (0.043) | (0.046) | (0.048) | (0.102) | (0.113) |
| Semi-urban envir. x Ln Distance |  | 0.081* | 0.044 |  | -0.040 | -0.034 |  | 0.311** |
|  |  | (0.045) | (0.043) |  | (0.037) | (0.039) |  | (0.119) |
| Structural characteristics | YES | YES |  | YES | YES |  | YES | YES |
| Host characteristics | YES | YES |  | YES | YES |  | YES | YES |
| Municipality fixed effects | YES | YES |  | YES | YES |  | YES | YES |
| Constant | 3.681*** | 3.659*** |  | 4.284*** | 4.237*** |  | 4.454*** | 3.745*** |
|  | (0.083) | (0.083) |  | (0.104) | (0.095) |  | (0.291) | (0.288) |
| Observations | 11,496 | 11,496 |  | 4,930 | 4,930 |  | 237 | 237 |
| R-squared | 0.709 | 0.710 |  | 0.773 | 0.776 |  | 0.761 | 0.820 |

**Table A18.** Separate WLS hedonic price regression estimates by island. Clustered standard errors at the beach level in parentheses. *** p<0.01, ** p<0.05, * p<0.1

Note: The reference categories are *Clear sand*, *Urban front*, *Easy Ac* and *Urban envir*


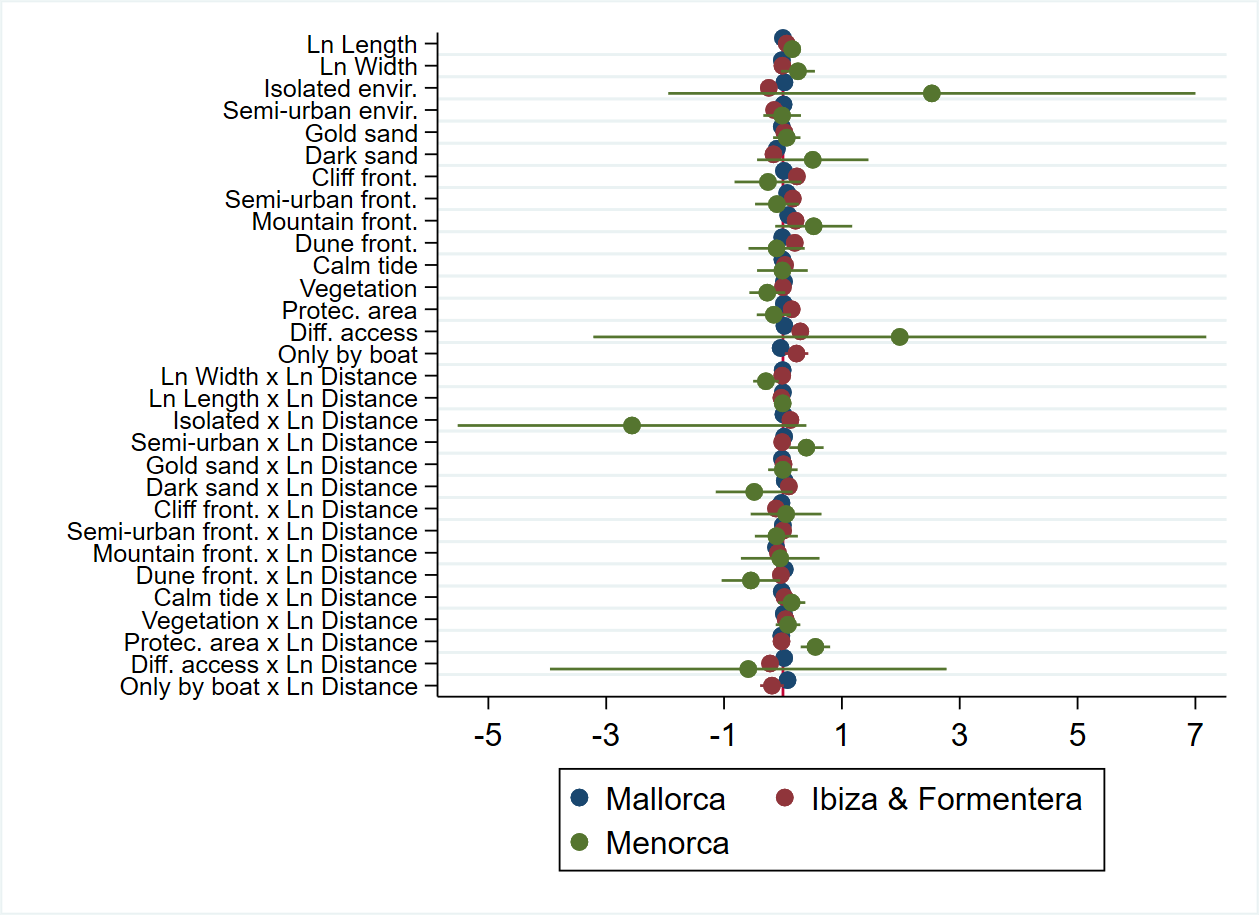


**Figure A13.** Coefficient estimates for separate regressions per island

|  | (1) | (2) | (3) |
| --- | --- | --- | --- |
|  | Mallorca-Ibiza | Mallorca-Menorca | Ibiza/Formentera-Menorca |
| Explanatory variables | Chi2(1) [pvalue] | Chi2(1) [pvalue] | Chi2(1)  [pvalue] |
|  |  |  |  |
| Ln Distance | 0.56 | 6.48 | 7.45 |
|  | [0.455] | [0.010] | [0.006] |
| Ln Length | 16.59 | 39.54 | 11.60 |
|  | [<0.001] | [<0.001] | [<0.001] |
| Ln Length x Ln distance | 5.60 | 0.02 | 1.04 |
|  | [0.018] | [0.880] | [0.306] |
| Ln Width | 0.15 | 27.83 | 24.93 |
|  | [0.697] | [<0.001] | [<0.001] |
| Ln Width x Ln distance | 0.22 | 10.40 | 9.74 |
|  | [0.641] | [0.001] | [0.001] |
| Gold sand | 1.36 | 3.42 | 0.78 |
|  | [0.244] | [0.064] | [0.377] |
| Gold sand x Ln Distance | 0.78 | 0.05 | 0.03 |
|  | [0.377] | [0.817] | [0.861] |
| Dark sand | 0.52 | 9.98 | 11.37 |
|  | [0.468] | [<0.001] | [<0.001] |
| Dark sand x Ln Distance | 1.27 | 15.15 | 17.96 |
|  | [0.260] | [<0.001] | [<0.001] |
| Cliff front. | 9.75 | 9.14 | 21.27 |
|  | [0.001] | [0.002] | [<0.001] |
| Cliff front. x Ln Distance | 2.21 | 0.71 | 2.61 |
|  | [0.137] | [0.398] | [0.106] |
| Semi-urban front. | 6.12 | 4.03 | 9.32 |
|  | [0.013] | [0.044] | [0.002] |
| Semi-urban front. x Ln Distance | 0.02 | 2.80 | 3.07 |
|  | [0.887] | [0.094] | [0.079] |
| Mountain front. | 1.82 | 7.60 | 3.95 |
|  | [0.177] | [0.005] | [0.046] |
| Mountain front. x Ln Distance | 0.24 | 0.22 | 0.06 |
|  | [0.623] | [0.638] | [0.807] |
| Dune front. | 6.38 | 1.06 | 7.49 |
|  | [0.011] | [0.302] | [0.006] |
| Dune front. x Ln Distance | 0.65 | 29.80 | 16.14 |
|  | [0.419] | [<0.001] | [<0.001] |
| Calm tide | 0.98 | 0.01 | 0.19 |
|  | [0.322] | [0.999] | [0.663] |
| Calm tide x Ln Distance | 0.98 | 5.10 | 2.20 |
|  | [0.322] | [0.024] | [0.137] |
| Vegetation | 0.23 | 18.51 | 14.60 |
|  | [0.628] | [<0.001] | [<0.001] |
| Vegetation x Ln Distance | 0.23 | 3.45 | 0.92 |
|  | [0.628] | [0.063] | [0.336] |
| Protec. area | 4.17 | 6.25 | 12.95 |
|  | [0.041] | [0.012] | [<0.001] |
| Protect. area x Ln Distance | 4.17 | 49.00 | 40.61 |
|  | [0.041] | [<0.001] | [<0.001] |
| Diff. access | 14.93 | 5.68 | 4.20 |
|  | [<0.001] | [0.017] | [0.040] |
| Diff. access x Ln Distance | 14.93 | 1.40 | 0.51 |
|  | [<0.001] | [0.237] | [0.477] |
| Only by boat | 5.90 |  |  |
|  | [0.015] |  |  |
| Only by boat x Ln Distance | 16.46 |  |  |
|  | [<0.001] |  |  |
| Isolated envir. | 19.84 | 22.38 | 27.23 |
|  | [<0.001] | [<0.001] | [<0.001] |
| Isolated envir. x Ln Distance | 6.87 | 48.00 | 52.24 |
|  | [0.008] | [<0.001] | [<0.001] |
| Semi-urban envir. | 8.73 | 0.14 | 3.44 |
|  | [0.003] | [0.707] | [0.063] |
| Semi-urban envir. x Ln Distance | 1.07 | 19.67 | 21.72 |
|  | [0.300] | [<0.001] | [<0.001] |
| All | 531.85 | 5263.51 | 5375.61 |
|  | [<0.001] | [<0.001] | [<0.001] |

**Table A19.** Pairwise coefficient comparison (chi squared test) from separate regressions by islands (Table A18)

|  | (1) | (2) | (3) |
| --- | --- | --- | --- |
|  | First stage (relevance condition) | 2SLS | Placebo check (exclusion restriction) |
| Dependent variable | Ln Width | Ln ADR | Ln ADR |
| Explanatory variables | Coeff. (SE) | Coeff. (SE) | Coeff. (SE) |
| Tourism Office | 0.455*** |  | -0.023 |
|  | (0.018) |  | (0.038) |
| Yacht club | -0.489*** |  | -0.004 |
|  | (0.016) |  | (0.024) |
| Telephone cabin | 0.172*** |  | -0.011 |
|  | (0.014) |  | (0.024) |
| Disabled access | 0.597*** |  | 0.010 |
|  | (0.013) |  | (0.023) |
| Ln Width |  | -0.017 | -0.020 |
|  |  | (0.015) | (0.014) |
| Ln Distance | -0.014*** | 0.001 | 0.001 |
|  | (0.004) | (0.004) | (0.007) |
| Ln Length | -0.049*** | 0.027*** | 0.027*** |
|  | (0.005) | (0.005) | (0.010) |
| Gold sand | -0.195*** | 0.014 | 0.012 |
|  | (0.012) | (0.014) | (0.025) |
| Dark sand | -0.369*** | -0.040 | -0.066 |
|  | (0.026) | (0.029) | (0.042) |
| Cliff front. | 0.381*** | 0.095*** | 0.097** |
|  | (0.020) | (0.021) | (0.038) |
| Semi-urban front. | 0.279*** | 0.116*** | 0.127*** |
|  | (0.013) | (0.013) | (0.043) |
| Mountain front. | 0.431*** | 0.090*** | 0.104** |
|  | (0.027) | (0.029) | (0.052) |
| Dune front. | 0.634*** | 0.113*** | 0.096* |
|  | (0.027) | (0.029) | (0.049) |
| Calm tide | 0.026** | -0.021* | -0.026 |
|  | (0.012) | (0.012) | (0.021) |
| Vegetation | 0.240*** | 0.051*** | 0.046* |
|  | (0.012) | (0.012) | (0.024) |
| Protec. area | -0.150*** | -0.007 | -0.009 |
|  | (0.017) | (0.018) | (0.025) |
| Diff. access | -0.601*** | 0.112*** | 0.093** |
|  | (0.025) | (0.032) | (0.041) |
| Only boat | -0.999*** | 0.093** | 0.042 |
|  | (0.037) | (0.043) | (0.053) |
| Isolated envir. | 0.075*** | -0.070*** | -0.072* |
|  | (0.023) | (0.023) | (0.042) |
| Semi-urban envir. | 0.207*** | -0.075*** | -0.081** |
|  | (0.016) | (0.015) | (0.034) |
| Structural characteristics | YES | YES | YES |
| Host characteristics | YES | YES | YES |
| Municipality fixed effects | YES | YES | YES |
| Constant | 2.642*** | 3.595*** | 3.602*** |
|  | (0.070) | (0.083) | (0.083) |
| F from first-stage [p-value] | 440.26 [<0.001] |  |  |
| Sargan test overidentifying restrictions [p-value] | 5.452 [0.141] |  |  |
| Durbin-Wu-Hausman test for exogeneity [p-value] | 0.005 [0.932] |  |  |
| Observations | 16,663 | 16,663 | 16,663 |
| R-squared | 0.703 | 0.746 | 0.737 |

**Table A20.** First stage, 2SLS and placebo regressions to test potential measurement error in ln Width. Clustered standard errors at the beach level in parentheses. *** p<0.01, ** p<0.05, * p<0.1

Note: The reference categories are *Clear sand*, *Urban front*, *Easy Ac* and *Urban envir*

| Dependent variable: Ln ADR | (1) | (2) | (3) | (4) |
| --- | --- | --- | --- | --- |
|  | Pooled OLS | Pooled OLS | Panel RE | Panel RE |
| Explanatory variables | Coeff. (SE) | Coeff. (SE) | Coeff. (SE) | Coeff. (SE) |
|  |  |  |  |  |
| Ln Distance | -0.001 | 0.023 | 0.000 | 0.022 |
|  | (0.007) | (0.040) | (0.007) | (0.035) |
| Ln Length | 0.020** | 0.020** | 0.020** | 0.021** |
|  | (0.009) | (0.009) | (0.009) | (0.009) |
| Ln Length x Ln distance |  | -0.005 |  | -0.006 |
|  |  | (0.005) |  | (0.005) |
| Ln Width | -0.020 | -0.013 | -0.018 | -0.012 |
|  | (0.014) | (0.014) | (0.013) | (0.013) |
| Ln Width x Ln distance |  | -0.011 |  | -0.010 |
|  |  | (0.009) |  | (0.009) |
| Gold sand | 0.003 | -0.004 | 0.008 | 0.002 |
|  | (0.025) | (0.026) | (0.023) | (0.023) |
| Gold sand x Ln Distance |  | 0.014 |  | 0.011 |
|  |  | (0.019) |  | (0.016) |
| Dark sand | -0.056 | -0.084** | -0.065 | -0.076** |
|  | (0.048) | (0.042) | (0.040) | (0.037) |
| Dark sand x Ln Distance |  | 0.041 |  | 0.016 |
|  |  | (0.025) |  | (0.024) |
| Cliff front. | 0.066** | 0.063** | 0.091*** | 0.089** |
|  | (0.032) | (0.031) | (0.035) | (0.035) |
| Cliff front. x Ln Distance |  | -0.014 |  | -0.015 |
|  |  | (0.025) |  | (0.023) |
| Semi-urban front. | 0.102*** | 0.106*** | 0.130*** | 0.133*** |
|  | (0.034) | (0.031) | (0.039) | (0.036) |
| Semi-urban front. x Ln Distance |  | 0.002 |  | 0.006 |
|  |  | (0.018) |  | (0.017) |
| Mountain front. | 0.047 | 0.117** | 0.073 | 0.130*** |
|  | (0.043) | (0.047) | (0.047) | (0.050) |
| Mountain front. x Ln Distance |  | -0.073** |  | -0.079** |
|  |  | (0.029) |  | (0.031) |
| Dune front. | 0.094** | 0.074 | 0.094** | 0.090* |
|  | (0.045) | (0.048) | (0.044) | (0.048) |
| Dune front. x Ln Distance |  | 0.028 |  | 0.006 |
|  |  | (0.032) |  | (0.027) |
| Calm tide | -0.019 | -0.017 | -0.029 | -0.027 |
|  | (0.020) | (0.021) | (0.021) | (0.022) |
| Calm tide x Ln Distance |  | -0.007 |  | -0.008 |
|  |  | (0.012) |  | (0.012) |
| Vegetation | 0.050** | 0.043** | 0.043* | 0.036* |
|  | (0.022) | (0.020) | (0.022) | (0.021) |
| Vegetation x Ln Distance |  | 0.026* |  | 0.015 |
|  |  | (0.013) |  | (0.014) |
| Protec. area | -0.002 | 0.039 | -0.010 | 0.023 |
|  | (0.026) | (0.025) | (0.024) | (0.026) |
| Protect. area x Ln Distance |  | -0.036** |  | -0.037** |
|  |  | (0.016) |  | (0.018) |
| Diff. access | 0.086** | 0.161*** | 0.095** | 0.167*** |
|  | (0.040) | (0.040) | (0.039) | (0.046) |
| Diff. access x Ln Distance |  | -0.072*** |  | -0.069** |
|  |  | (0.027) |  | (0.031) |
| Only by boat | 0.106* | 0.018 | 0.072 | 0.039 |
|  | (0.054) | (0.063) | (0.050) | (0.064) |
| Only by boat x Ln Distance |  | 0.060 |  | 0.012 |
|  |  | (0.041) |  | (0.038) |
| Isolated envir. | -0.042 | -0.067* | -0.057 | -0.082** |
|  | (0.039) | (0.036) | (0.039) | (0.039) |
| Isolated envir. x Ln Distance |  | 0.013 |  | 0.029 |
|  |  | (0.025) |  | (0.024) |
| Semi-urban envir. | -0.048 | -0.055* | -0.067* | -0.072** |
|  | (0.034) | (0.031) | (0.034) | (0.032) |
| Semi-urban envir. x Ln Distance |  | 0.018 |  | 0.022 |
|  |  | (0.017) |  | (0.017) |
| Day 2 | 0.001 | 0.000 | -0.002 | -0.002 |
|  | (0.004) | (0.004) | (0.001) | (0.001) |
| Day 3 | 0.000 | -0.000 | -0.001 | -0.001 |
|  | (0.004) | (0.004) | (0.001) | (0.001) |
| Day 4 | 0.001 | 0.001 | -0.000 | -0.000 |
|  | (0.004) | (0.004) | (0.001) | (0.001) |
| Day 5 | 0.010* | 0.010** | 0.004* | 0.004* |
|  | (0.005) | (0.005) | (0.003) | (0.003) |
| Day 6 | 0.016*** | 0.016*** | 0.006*** | 0.006*** |
|  | (0.005) | (0.005) | (0.002) | (0.002) |
| Day 7 | 0.010** | 0.010** | 0.003* | 0.003* |
|  | (0.005) | (0.005) | (0.002) | (0.002) |
| Day 8 | 0.006 | 0.006 | 0.000 | 0.000 |
|  | (0.005) | (0.005) | (0.001) | (0.001) |
| Day 9 | 0.006 | 0.007 | 0.001 | 0.001 |
|  | (0.005) | (0.005) | (0.002) | (0.002) |
| Day 10 | 0.007 | 0.007 | 0.001 | 0.001 |
|  | (0.005) | (0.005) | (0.002) | (0.002) |
| Day 11 | 0.011*** | 0.012*** | 0.001 | 0.001 |
|  | (0.004) | (0.004) | (0.002) | (0.002) |
| Day 12 | 0.019*** | 0.019*** | 0.007*** | 0.007*** |
|  | (0.004) | (0.004) | (0.002) | (0.002) |
| Day 13 | 0.020*** | 0.020*** | 0.008*** | 0.008*** |
|  | (0.004) | (0.004) | (0.002) | (0.002) |
| Day 14 | 0.012** | 0.012** | 0.004* | 0.004* |
|  | (0.005) | (0.005) | (0.002) | (0.002) |
| Day 15 | 0.010** | 0.010** | 0.003* | 0.003* |
|  | (0.005) | (0.005) | (0.002) | (0.002) |
| Day 16 | 0.009* | 0.009* | 0.004** | 0.004** |
|  | (0.005) | (0.005) | (0.002) | (0.002) |
| Day 17 | 0.006 | 0.006 | 0.004** | 0.004** |
|  | (0.005) | (0.005) | (0.002) | (0.002) |
| Day 18 | 0.006 | 0.006 | 0.006*** | 0.006*** |
|  | (0.005) | (0.005) | (0.002) | (0.002) |
| Day 19 | 0.013*** | 0.012*** | 0.009*** | 0.009*** |
|  | (0.005) | (0.005) | (0.002) | (0.002) |
| Day 20 | 0.006 | 0.006 | 0.008*** | 0.008*** |
|  | (0.005) | (0.005) | (0.002) | (0.002) |
| Day 21 | -0.004 | -0.004 | 0.001 | 0.001 |
|  | (0.005) | (0.005) | (0.002) | (0.002) |
| Day 22 | -0.019*** | -0.019*** | -0.004** | -0.004** |
|  | (0.005) | (0.005) | (0.002) | (0.002) |
| Day 23 | -0.017*** | -0.018*** | -0.006*** | -0.006*** |
|  | (0.005) | (0.005) | (0.002) | (0.002) |
| Day 24 | -0.020*** | -0.021*** | -0.006*** | -0.006*** |
|  | (0.005) | (0.005) | (0.002) | (0.002) |
| Day 25 | -0.022*** | -0.022*** | -0.007*** | -0.007*** |
|  | (0.005) | (0.005) | (0.002) | (0.002) |
| Day 26 | -0.017*** | -0.017*** | -0.004* | -0.004* |
|  | (0.005) | (0.005) | (0.002) | (0.002) |
| Day 27 | -0.023*** | -0.023*** | -0.007** | -0.007** |
|  | (0.005) | (0.005) | (0.003) | (0.003) |
| Day 28 | -0.040*** | -0.040*** | -0.016*** | -0.016*** |
|  | (0.006) | (0.006) | (0.003) | (0.003) |
| Day 29 | -0.026*** | -0.026*** | -0.026*** | -0.026*** |
|  | (0.007) | (0.007) | (0.003) | (0.003) |
| Day 30 | -0.036*** | -0.036*** | -0.028*** | -0.028*** |
|  | (0.008) | (0.008) | (0.003) | (0.003) |
| Day 31 | -0.046*** | -0.046*** | -0.032*** | -0.032*** |
|  | (0.008) | (0.008) | (0.004) | (0.004) |
| Structural characteristics | YES | YES | YES | YES |
| Host characteristics | YES | YES | YES | YES |
| Municipality fixed effects | YES | YES | YES | YES |
| Constant | 3.610*** | 3.588*** | 3.575*** | 3.566*** |
|  | (0.074) | (0.075) | (0.071) | (0.071) |
| Observations | 231,314 | 231,314 | 231,314 | 231,314 |
| R-squared | 0.724 | 0.726 |  |  |

**Table A21.** OLS hedonic price regression estimates using daily data. Columns 1 and 2 presents the results from a Pooled OLS regression. Columns 3 and 4 report the estimates from a panel data regression with random effects. Clustered standard errors at the beach level in parentheses. *** p<0.01, ** p<0.05, * p<0.1

Note: The reference categories are *Clear sand*, *Urban front*, *Easy Ac* and *Urban envir*


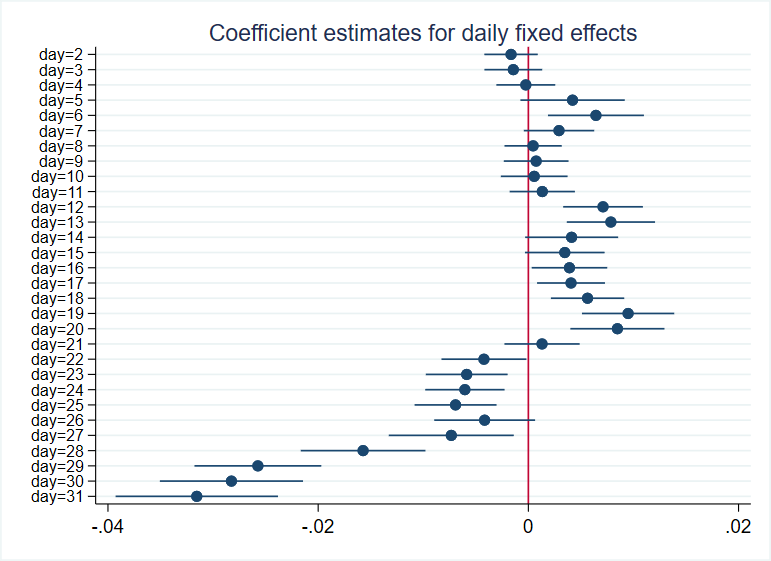


**Figure A14.** Coefficient estimates for daily fixed effects after a random effects panel regression (column 4 in Table A21).

| Dependent variable: Ln ADR | (1) | (2) |
| --- | --- | --- |
|  | Only properties reserved for more than 15 days | Only properties reserved for more than 15 days |
| Explanatory variables | Coeff. (SE) | Coeff. (SE) |
|  |  |  |
| Ln Distance | 2.4e-04 | 0.037 |
|  | (0.008) | (0.044) |
| Ln Length | 0.027*** | 0.027*** |
|  | (0.010) | (0.010) |
| Ln Length x Ln distance |  | -0.007 |
|  |  | (0.006) |
| Ln Width | -0.012 | -0.005 |
|  | (0.014) | (0.014) |
| Ln Width x Ln distance |  | -0.017 |
|  |  | (0.010) |
| Gold sand | 0.015 | 0.012 |
|  | (0.026) | (0.026) |
| Gold sand x Ln Distance |  | 0.017 |
|  |  | (0.019) |
| Dark sand | -0.026 | -0.047 |
|  | (0.048) | (0.042) |
| Dark sand x Ln Distance |  | 0.031 |
|  |  | (0.025) |
| Cliff front. | 0.082** | 0.082** |
|  | (0.034) | (0.035) |
| Cliff front. x Ln Distance |  | -0.015 |
|  |  | (0.032) |
| Semi-urban front. | 0.107*** | 0.109*** |
|  | (0.040) | (0.037) |
| Semi-urban front. x Ln Distance |  | 0.007 |
|  |  | (0.020) |
| Mountain front. | 0.084* | 0.110** |
|  | (0.048) | (0.048) |
| Mountain front. x Ln Distance |  | -0.036 |
|  |  | (0.033) |
| Dune front. | 0.123** | 0.107** |
|  | (0.049) | (0.051) |
| Dune front. x Ln Distance |  | 0.031 |
|  |  | (0.037) |
| Calm tide | -0.020 | -0.016 |
|  | (0.023) | (0.024) |
| Calm tide x Ln Distance |  | -0.006 |
|  |  | (0.014) |
| Vegetation | 0.057** | 0.053** |
|  | (0.023) | (0.021) |
| Vegetation x Ln Distance |  | 0.027* |
|  |  | (0.016) |
| Protec. area | -0.008 | 0.031 |
|  | (0.029) | (0.030) |
| Protect. area x Ln Distance |  | -0.028 |
|  |  | (0.018) |
| Diff. access | 0.122** | 0.229*** |
|  | (0.050) | (0.044) |
| Diff. access x Ln Distance |  | -0.127*** |
|  |  | (0.029) |
| Only by boat | 0.131** | 0.096 |
|  | (0.057) | (0.066) |
| Only by boat x Ln Distance |  | 0.024 |
|  |  | (0.050) |
| Isolated envir. | -0.077* | -0.106*** |
|  | (0.042) | (0.040) |
| Isolated envir. x Ln Distance |  | 0.009 |
|  |  | (0.029) |
| Semi-urban envir. | -0.075** | -0.080** |
|  | (0.035) | (0.033) |
| Semi-urban envir. x Ln Distance |  | 0.011 |
|  |  | (0.021) |
| Structural characteristics | YES | YES |
| Host characteristics | YES | YES |
| Municipality fixed effects | YES | YES |
| Constant | 3.581*** | 3.540*** |
|  | (0.079) | (0.081) |
| Observations | 9,306 | 9,306 |
| R-squared | 0.745 | 0.747 |

**Table A22.** WLS hedonic price regression estimates considering only properties reserved for more than 15 days. Clustered standard errors at the beach level in parentheses. *** p<0.01, ** p<0.05, * p<0.1

Note: The reference categories are *Clear sand*, *Urban front*, *Easy Ac* and *Urban envir*

| Dependent variable: Ln ADR | (1) | (2) |
| --- | --- | --- |
|  | All sample without weighting | All sample without weighting |
| Explanatory variables | Coeff. (SE) | Coeff. (SE) |
|  |  |  |
| Ln Distance | 0.001 | 0.022 |
|  | (0.007) | (0.035) |
| Ln Length | 0.025*** | 0.027*** |
|  | (0.009) | (0.010) |
| Ln Length x Ln distance |  | -0.007 |
|  |  | (0.005) |
| Ln Width | -0.021* | -0.014 |
|  | (0.013) | (0.013) |
| Ln Width x Ln distance |  | -0.011 |
|  |  | (0.008) |
| Gold sand | 0.013 | 0.006 |
|  | (0.024) | (0.024) |
| Gold sand x Ln Distance |  | 0.012 |
|  |  | (0.015) |
| Dark sand | -0.065 | -0.071* |
|  | (0.041) | (0.038) |
| Dark sand x Ln Distance |  | 0.010 |
|  |  | (0.023) |
| Cliff front. | 0.107*** | 0.104*** |
|  | (0.036) | (0.036) |
| Cliff front. x Ln Distance |  | -0.014 |
|  |  | (0.023) |
| Semi-urban front. | 0.134*** | 0.137*** |
|  | (0.040) | (0.038) |
| Semi-urban front. x Ln Distance |  | 0.009 |
|  |  | (0.016) |
| Mountain front. | 0.111** | 0.163*** |
|  | (0.050) | (0.052) |
| Mountain front. x Ln Distance |  | -0.076** |
|  |  | (0.031) |
| Dune front. | 0.106** | 0.110** |
|  | (0.046) | (0.050) |
| Dune front. x Ln Distance |  | -0.004 |
|  |  | (0.028) |
| Calm tide | -0.027 | -0.025 |
|  | (0.022) | (0.022) |
| Calm tide x Ln Distance |  | -0.008 |
|  |  | (0.012) |
| Vegetation | 0.041* | 0.034 |
|  | (0.023) | (0.022) |
| Vegetation x Ln Distance |  | 0.017 |
|  |  | (0.013) |
| Protec. area | -0.008 | 0.024 |
|  | (0.025) | (0.027) |
| Protect. area x Ln Distance |  | -0.041** |
|  |  | (0.018) |
| Diff. access | 0.091** | 0.168*** |
|  | (0.041) | (0.046) |
| Diff. access x Ln Distance |  | -0.083*** |
|  |  | (0.032) |
| Only by boat | 0.043 | -0.004 |
|  | (0.053) | (0.063) |
| Only by boat x Ln Distance |  | 0.017 |
|  |  | (0.038) |
| Isolated envir. | -0.071* | -0.098** |
|  | (0.040) | (0.040) |
| Isolated envir. x Ln Distance |  | 0.036 |
|  |  | (0.026) |
| Semi-urban envir. | -0.079** | -0.081** |
|  | (0.034) | (0.032) |
| Semi-urban envir. x Ln Distance |  | 0.026 |
|  |  | (0.016) |
| Structural characteristics | YES | YES |
| Host characteristics | YES | YES |
| Municipality fixed effects | YES | YES |
| Constant | 3.586*** | 3.580*** |
|  | (0.070) | (0.070) |
| Observations | 16,663 | 16,663 |
| R-squared | 0.737 | 0.738 |

**Table A23.** OLS hedonic price regression estimates without weighting observations by the number of days reserved. Clustered standard errors at the beach level in parentheses. *** p<0.01, ** p<0.05, * p<0.1

Note: The reference categories are *Clear sand*, *Urban front*, *Easy Ac* and *Urban envir*

| Dependent variable: Ln ADR | (1) | (2) |
| --- | --- | --- |
| Explanatory variables | Coeff. (SE) | Coeff. (SE) |
|  |  |  |
| Ln Distance | -0.013 | 0.023 |
|  | (0.009) | (0.036) |
| Ln Length | 0.007 | 0.007 |
|  | (0.007) | (0.006) |
| Ln Length x Ln distance |  | -0.005 |
|  |  | (0.005) |
| Ln Width | -0.011 | -0.002 |
|  | (0.012) | (0.011) |
| Ln Width x Ln distance |  | -0.014* |
|  |  | (0.008) |
| Gold sand | 0.046** | 0.046** |
|  | (0.021) | (0.020) |
| Gold sand x Ln Distance |  | -0.001 |
|  |  | (0.015) |
| Dark sand | 0.013 | -0.008 |
|  | (0.040) | (0.041) |
| Dark sand x Ln Distance |  | 0.026 |
|  |  | (0.025) |
| Cliff front. | 0.104*** | 0.131*** |
|  | (0.038) | (0.038) |
| Cliff front. x Ln Distance |  | -0.041 |
|  |  | (0.025) |
| Semi-urban front. | 0.147*** | 0.155*** |
|  | (0.037) | (0.034) |
| Semi-urban front. x Ln Distance |  | -0.010 |
|  |  | (0.016) |
| Mountain front. | 0.110** | 0.177*** |
|  | (0.048) | (0.048) |
| Mountain front. x Ln Distance |  | -0.088*** |
|  |  | (0.031) |
| Dune front. | 0.141*** | 0.145*** |
|  | (0.046) | (0.051) |
| Dune front. x Ln Distance |  | 0.005 |
|  |  | (0.038) |
| Calm tide | -0.024 | -0.022 |
|  | (0.018) | (0.020) |
| Calm tide x Ln Distance |  | 0.001 |
|  |  | (0.014) |
| Vegetation | 0.035** | 0.027* |
|  | (0.015) | (0.015) |
| Vegetation x Ln Distance |  | 0.025* |
|  |  | (0.013) |
| Protec. area | -0.034 | 0.006 |
|  | (0.025) | (0.025) |
| Protect. area x Ln Distance |  | -0.029* |
|  |  | (0.016) |
| Diff. access | 0.089** | 0.137*** |
|  | (0.035) | (0.034) |
| Diff. access x Ln Distance |  | -0.050** |
|  |  | (0.025) |
| Only by boat | 0.066* | 0.016 |
|  | (0.038) | (0.050) |
| Only by boat x Ln Distance |  | 0.020 |
|  |  | (0.043) |
| Isolated envir. | -0.074* | -0.111*** |
|  | (0.038) | (0.038) |
| Isolated envir. x Ln Distance |  | 0.049 |
|  |  | (0.041) |
| Semi-urban envir. | -0.070** | -0.079*** |
|  | (0.031) | (0.029) |
| Semi-urban envir. x Ln Distance |  | 0.022 |
|  |  | (0.015) |
| Structural characteristics | YES | YES |
| Host characteristics | YES | YES |
| Postal code fixed effects | YES | YES |
| Constant | 3.683*** | 3.661*** |
|  | (0.063) | (0.059) |
| Observations | 16,663 | 16,663 |
| R-squared | 0.756 | 0.757 |

**Table A24.** WLS hedonic price regression estimates considering postal code fixed effects instead of municipality fixed effects. Clustered standard errors at the beach level in parentheses. *** p<0.01, ** p<0.05, * p<0.1

Note: The reference categories are *Clear sand*, *Urban front*, *Easy Ac* and *Urban envir*

**Municipality socioeconomic characteristics and competitors**

We collected a wide set of population socio-economic characteristics for the 67 municipalities where Airbnb listings are located. This information is drawn from the 2011 Balearic Census and the Household Income Distribution Atlas for the year 2016 (INE, 2021). For each municipality, the dataset includes the following information: population size, average age, percentage of foreign citizens, percentage of population with low education, average household size (number of people), share of large dwellings, average gross income and Gini inequality index.

Moreover, to control for the degree of market competition in each municipality, we include the number of Airbnb listings (other than self-listing) and the number of hotel beds. The former is calculated from AirDNA data. The latter is drawn from the Balearic Islands Statistics Office for the year 2016.

| **Variable** | **Description** | **Mean** | **SD** | **Min** | **Max** |
| --- | --- | --- | --- | --- | --- |
| *Socioeconomic indicators* | |  |  |  |  |
| Pop | Population (Census 2011) | 88,863.46 | 144,653.10 | 260.00 | 400,370 |
| Av. Age | Average age (2016) | 40.44 | 1.58 | 38.40 | 51.40 |
| % Foreign | Percentage of foreign citizens (Census 2011) | 0.23 | 0.06 | 0.00 | 0.35 |
| % Low educ | Percentage of population with low education (Census 2011) | 0.07 | 0.03 | 0.03 | 0.19 |
| Av. House size | Average household size (Census 2011) | 0.27 | 0.13 | 2.03 | 3.03 |
| % Large dwellings | Percentage big dwelling (Census 2011) | 16.83 | 36.50 | 0.59 | 610.91 |
| Gross Income | Average gross Income (2016) | 14,053.44 | 1,384.10 | 11,570.14 | 23,158 |
| Gini | Gini Index (2016) | 32.93 | 1.94 | 23.92 | 44.60 |
| *Competition* |  |  |  |  |  |
| Airbnb listings | Number of Airbnb listings in the neighbourhood (postal code) | 358.57 | 438.75 | 0.00 | 1,589 |
| Hotel beds | Number of Hotels beds in the neighbourhood | 11,885.65 | 12,740.75 | 0.00 | 35,455 |

Table A25 presents summary statistics of these variables.

**Table A25.** Summary statistics of municipality socioeconomic indicators and competitors

| Dependent variable: Ln ADR | (1) | (2) |
| --- | --- | --- |
| Explanatory variables | Coeff. (SE) | Coeff. (SE) |
|  |  |  |
| Ln Distance | -0.025** | 0.005 |
|  | (0.012) | (0.054) |
| Ln Length | 0.009 | 0.022 |
|  | (0.021) | (0.023) |
| Ln Length x Ln distance |  | -0.021*** |
|  |  | (0.008) |
| Ln Width | -0.066*** | -0.064** |
|  | (0.024) | (0.025) |
| Ln Width x Ln distance |  | -0.013 |
|  |  | (0.014) |
| Gold sand | 0.096** | 0.096** |
|  | (0.037) | (0.038) |
| Gold sand x Ln Distance |  | 0.007 |
|  |  | (0.026) |
| Dark sand | -0.042 | -0.076 |
|  | (0.062) | (0.076) |
| Dark sand x Ln Distance |  | 0.014 |
|  |  | (0.034) |
| Cliff front. | 0.135*** | 0.139*** |
|  | (0.050) | (0.050) |
| Cliff front. x Ln Distance |  | -0.001 |
|  |  | (0.032) |
| Semi-urban front. | 0.119** | 0.141*** |
|  | (0.057) | (0.050) |
| Semi-urban front. x Ln Distance |  | 0.014 |
|  |  | (0.023) |
| Mountain front. | 0.184*** | 0.198*** |
|  | (0.068) | (0.071) |
| Mountain front. x Ln Distance |  | -0.052 |
|  |  | (0.043) |
| Dune front. | 0.220** | 0.203** |
|  | (0.087) | (0.095) |
| Dune front. x Ln Distance |  | 0.028 |
|  |  | (0.046) |
| Calm tide | 0.056* | 0.072** |
|  | (0.030) | (0.033) |
| Calm tide x Ln Distance |  | -0.016 |
|  |  | (0.020) |
| Vegetation | -0.014 | -0.019 |
|  | (0.046) | (0.045) |
| Vegetation x Ln Distance |  | 0.021 |
|  |  | (0.023) |
| Protec. area | -0.110** | -0.069 |
|  | (0.046) | (0.046) |
| Protect. area x Ln Distance |  | -0.037 |
|  |  | (0.024) |
| Diff. access | -0.010 | 0.138** |
|  | (0.072) | (0.068) |
| Diff. access x Ln Distance |  | -0.133*** |
|  |  | (0.034) |
| Only by boat | -0.156** | -0.021 |
|  | (0.079) | (0.110) |
| Only by boat x Ln Distance |  | -0.100* |
|  |  | (0.060) |
| Isolated envir. | -0.081 | -0.114* |
|  | (0.057) | (0.058) |
| Isolated envir. x Ln Distance |  | 0.007 |
|  |  | (0.037) |
| Semi-urban envir. | -0.023 | -0.012 |
|  | (0.052) | (0.044) |
| Semi-urban envir. x Ln Distance |  | -0.015 |
|  |  | (0.024) |
| Pop | -4.7e-07* | -5.0e-07* |
|  | (2.75e-07) | (2.9e-07) |
| Av. Age | -0.078*** | -0.078*** |
|  | (0.029) | (0.029) |
| % Foreign | -0.175 | -0.407 |
|  | (0.429) | (0.443) |
| % Low educ | 0.291 | 0.353 |
|  | (1.224) | (1.310) |
| Av. House size | 0.277 | 0.285 |
|  | (0.174) | (0.174) |
| % Large dwellings | 4.8e-04 | 0.001 |
|  | (0.001) | (4.7e-04) |
| Gross Income | 1.9e-05 | 1.2e-05 |
|  | (1.9e-05) | (2.0e-05) |
| Gini | 0.053*** | 0.056*** |
|  | (0.010) | (0.009) |
| Airbnb listings | 1.7e-04*** | 1.7e-04*** |
|  | (5.2e-05) | (5.2e-05) |
| Hotel beds | -2.9e-06 | -2.48e-06 |
|  | (3.5e-06) | (3.7e-06) |
| Structural characteristics | YES | YES |
| Host characteristics | YES | YES |
| Constant | 4.371*** | 4.411*** |
|  | (1.445) | (1.444) |
| VIF | 2.60 | 5.02 |
| Observations | 16,663 | 16,663 |
| R-squared | 0.708 | 0.712 |

**Table A26.** WLS hedonic price regression estimates including municipality controls instead of municipality fixed effects. Clustered standard errors at the beach level in parentheses. *** p<0.01, ** p<0.05, * p<0.1

Note: The reference categories are *Clear sand*, *Urban front*, *Easy Ac* and *Urban envir.*


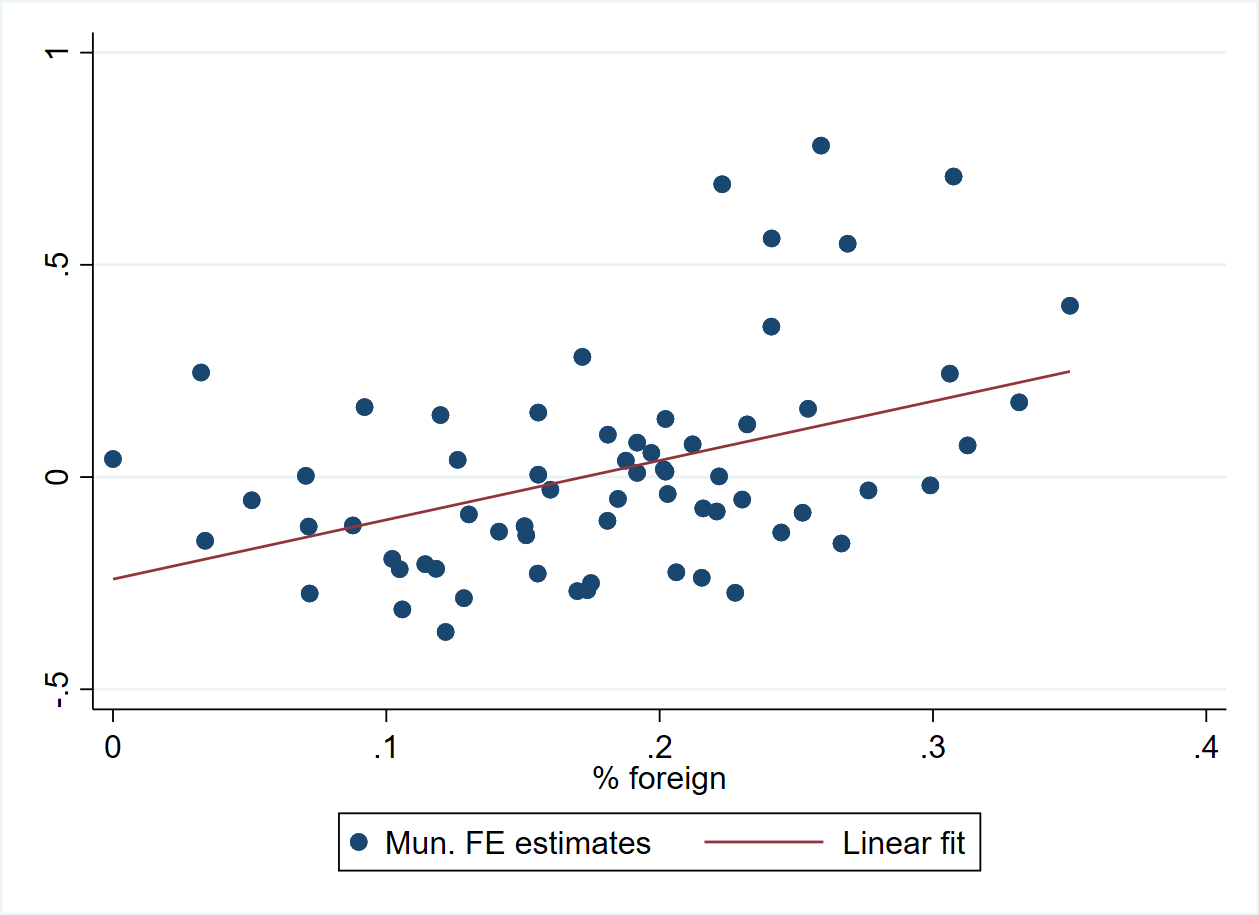


**Figure A15.** Scatter plot of Municipality fixed effects on % foreign people in the municipality


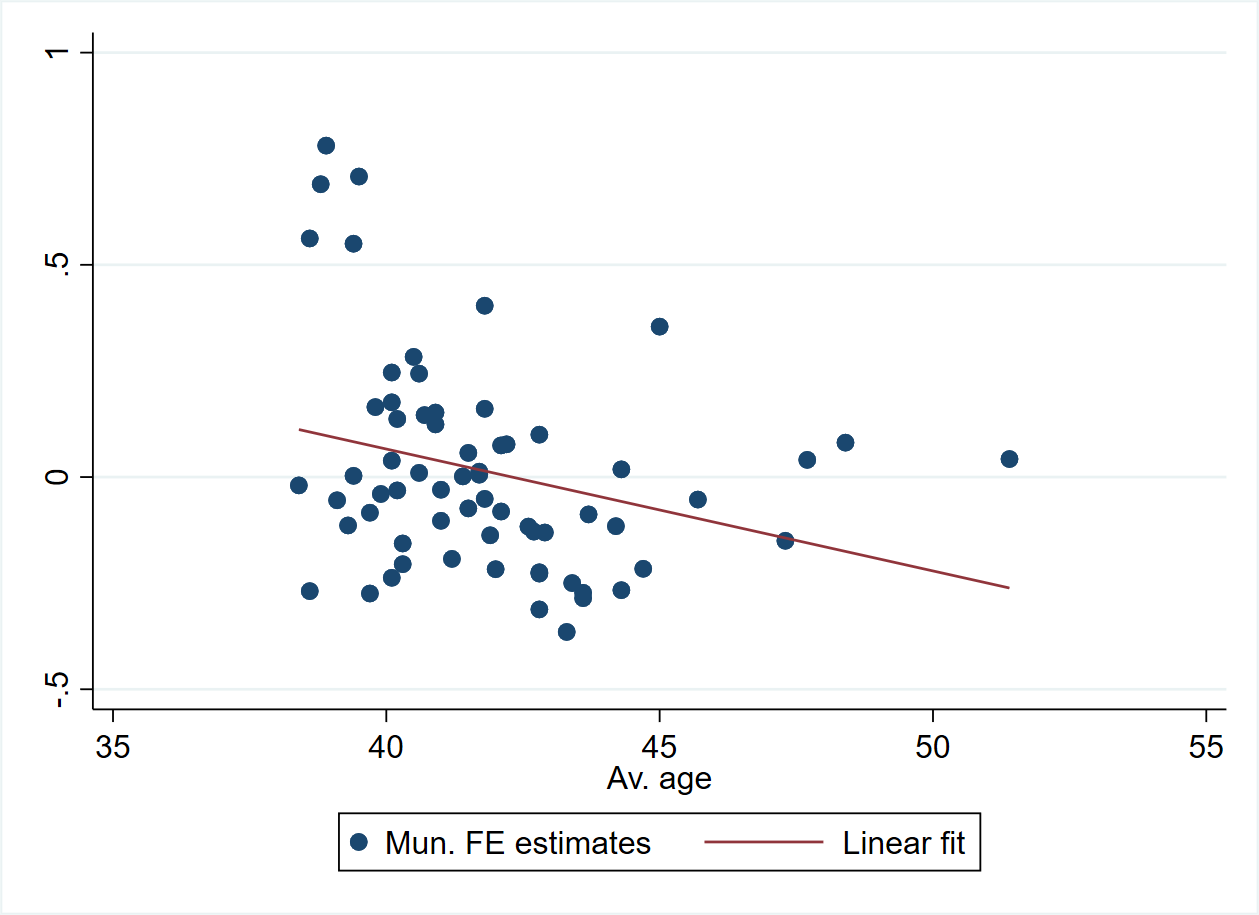


**Figure A16.** Scatter plot of Municipality fixed effects on average age of the municipality


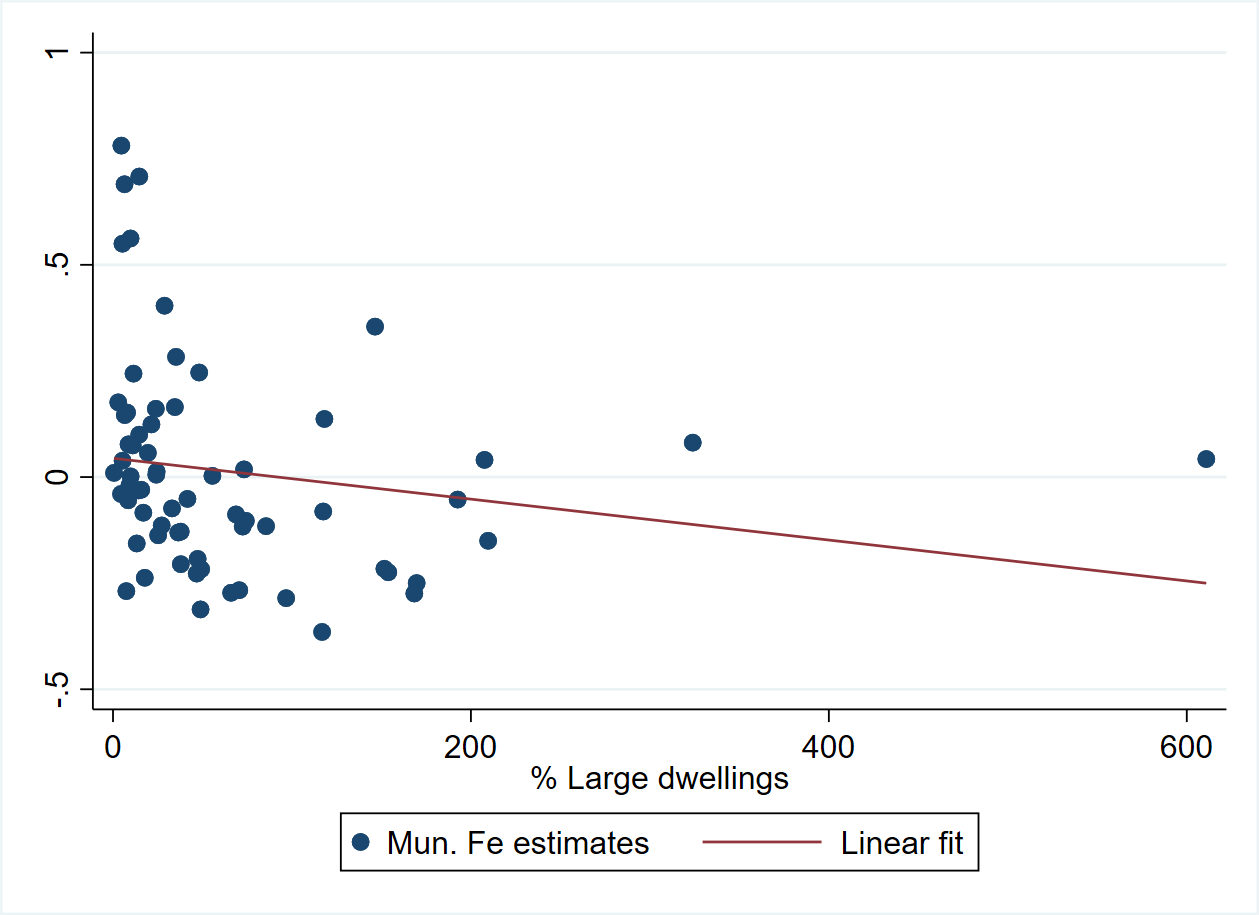


**Figure A17.** Scatter plot of Municipality fixed effects on % large dwellings in the municipality


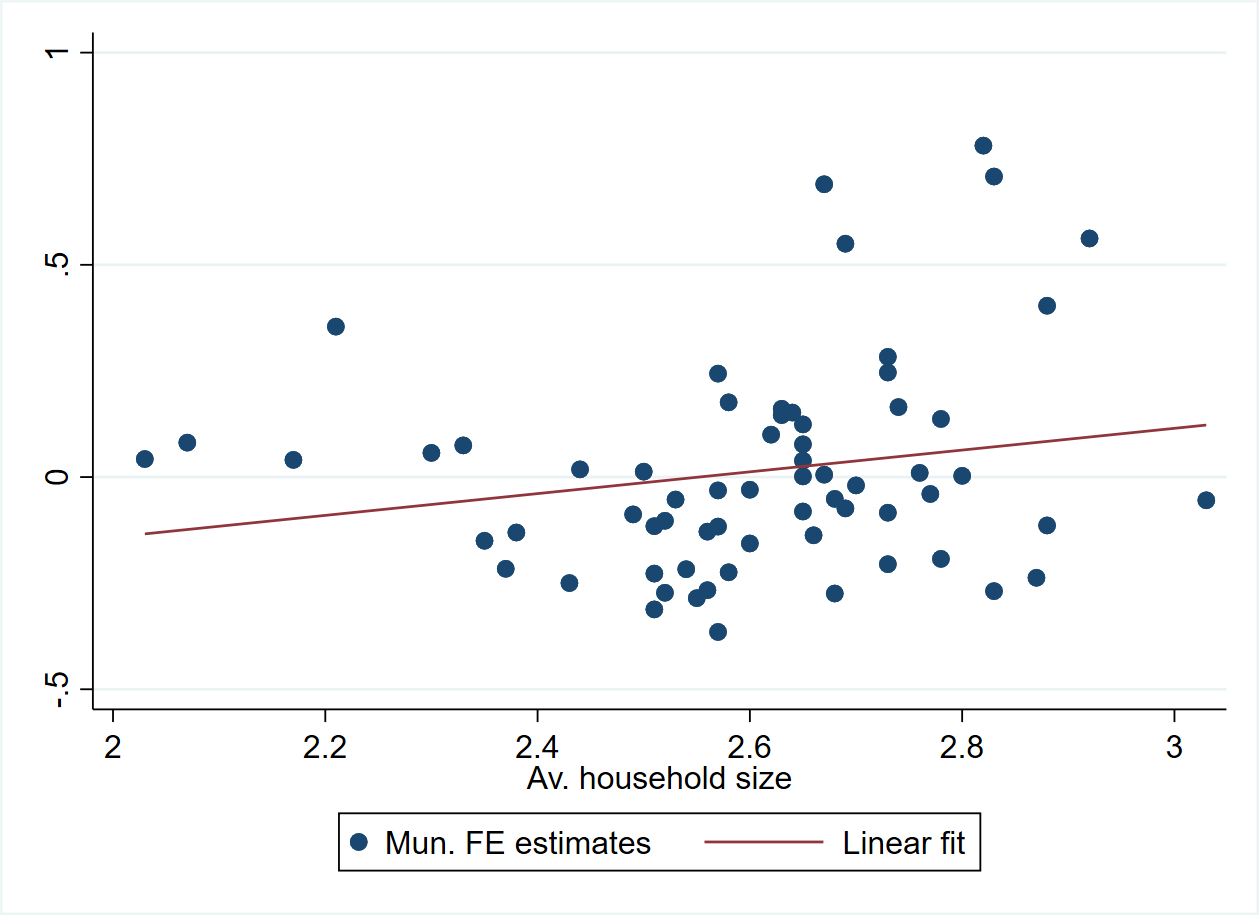


**Figure A18.** Scatter plot of Municipality fixed effects on average household size in the municipality


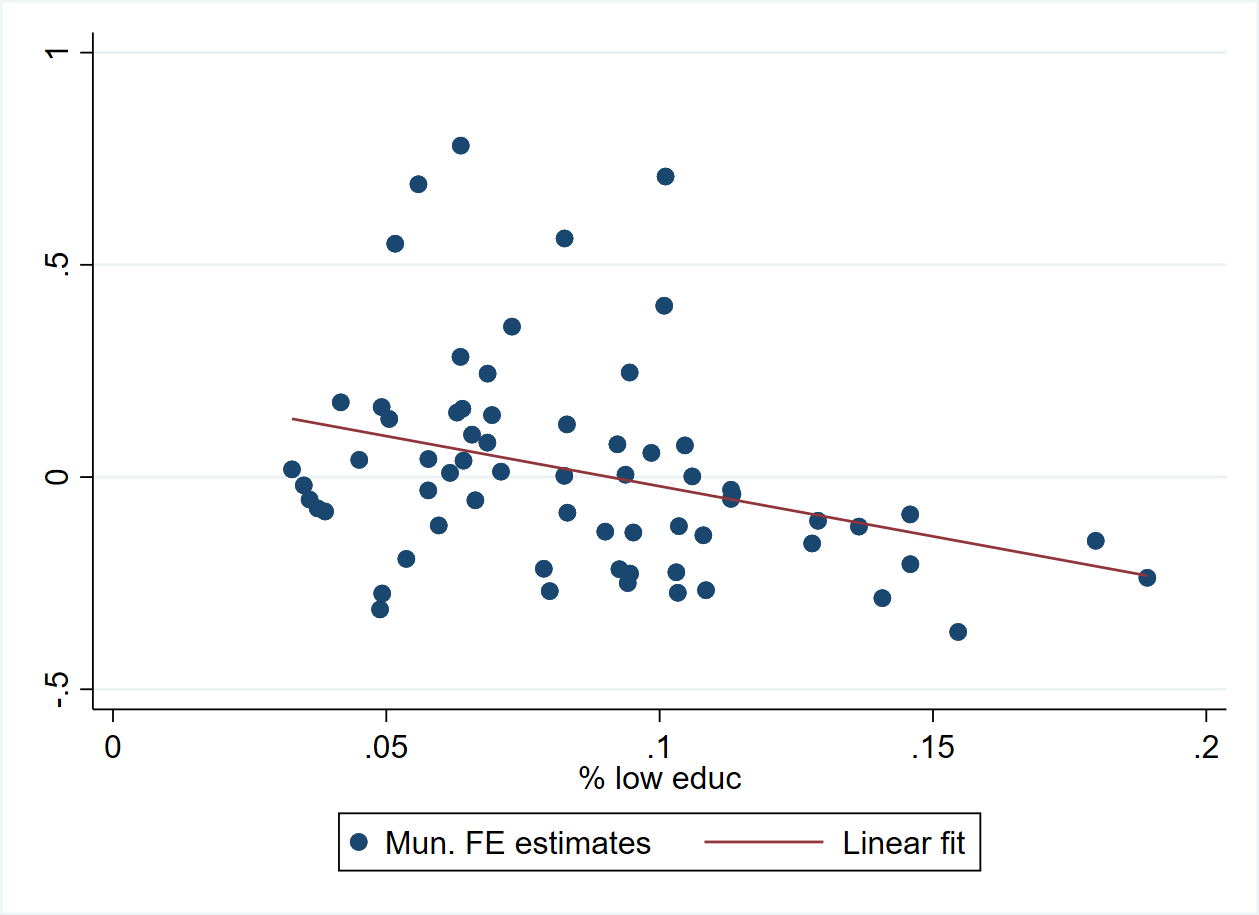


**Figure A19.** Scatter plot of Municipality fixed effects on % people with low education in the municipality


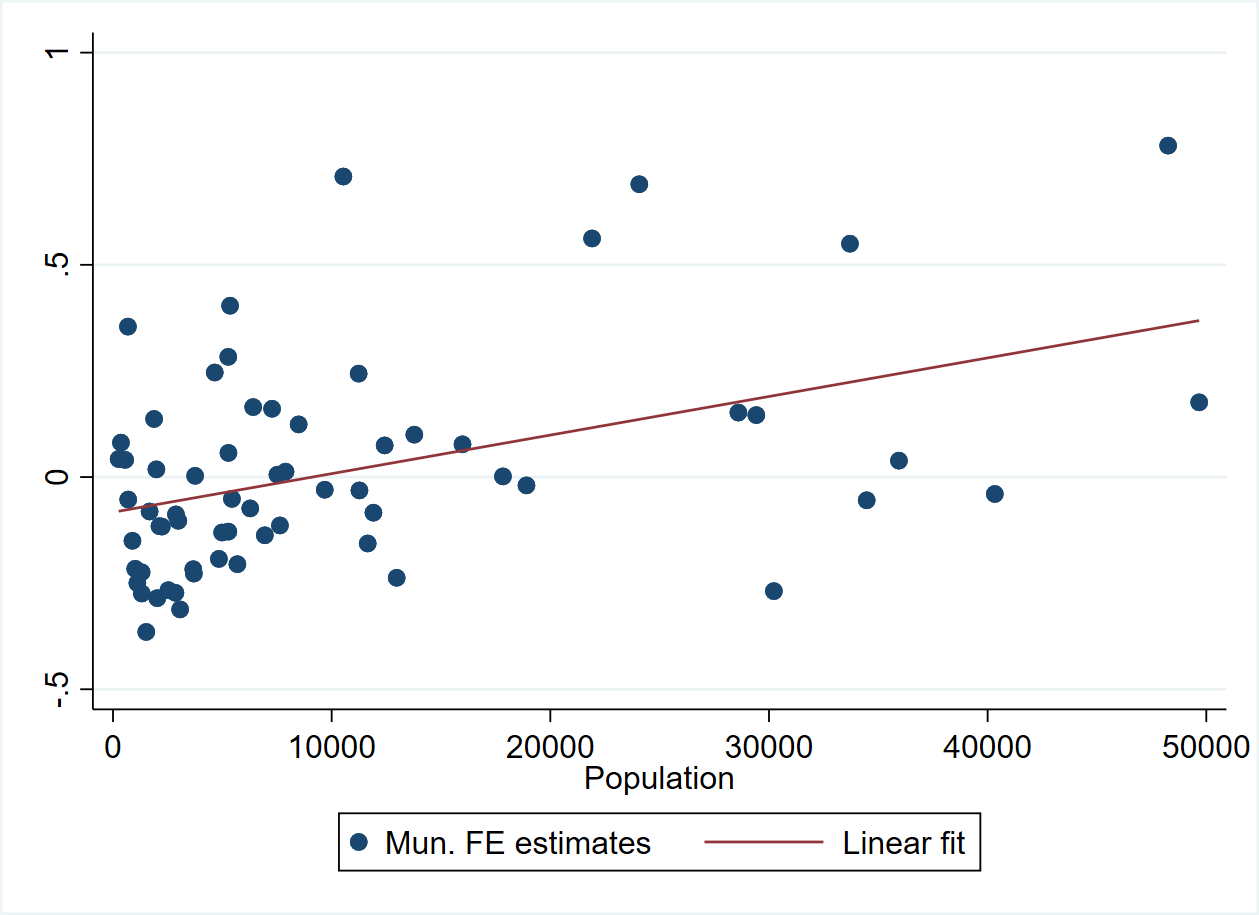


**Figure A20.** Scatter plot of Municipality fixed effects on population of the municipality


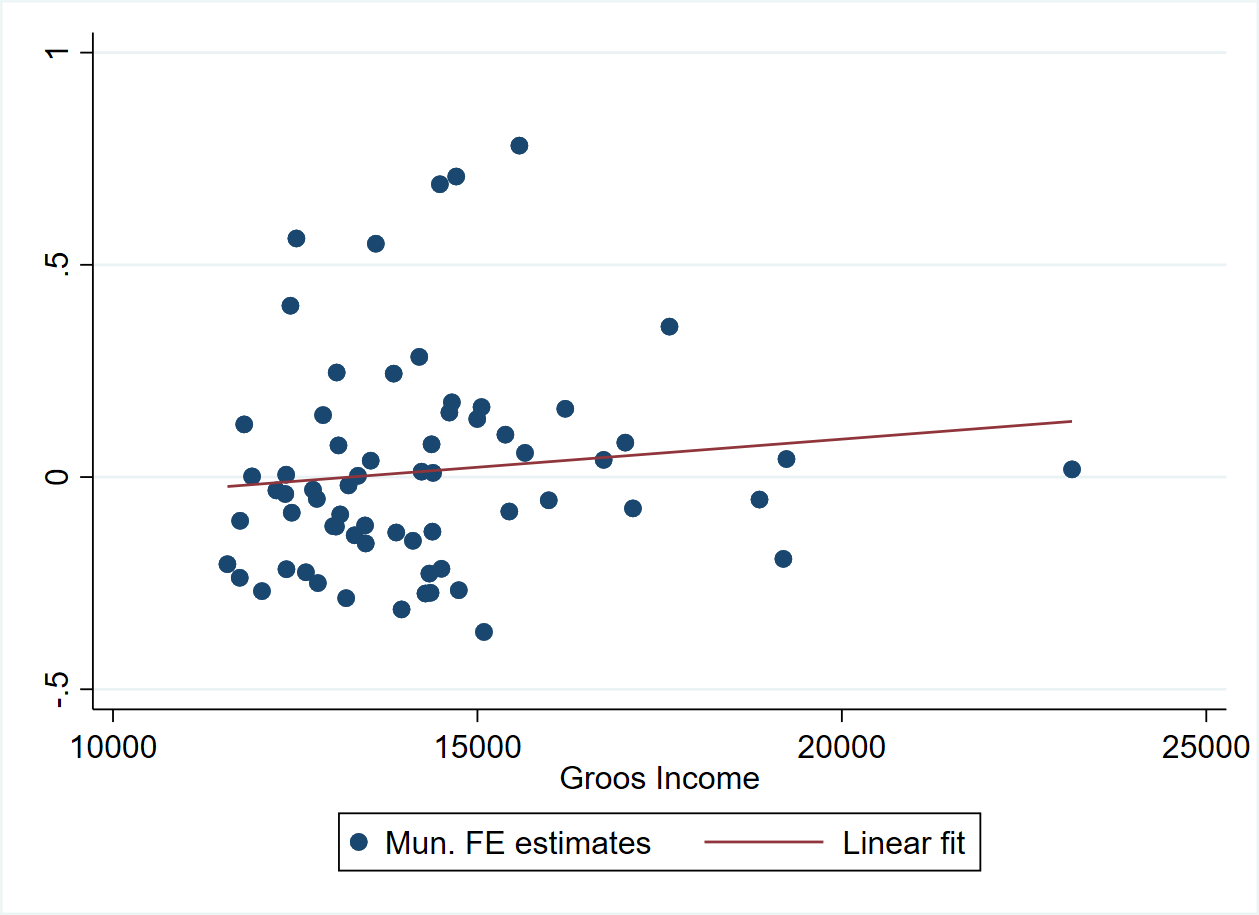


**Figure A21.** Scatter plot of Municipality fixed effects on gross income in the municipality


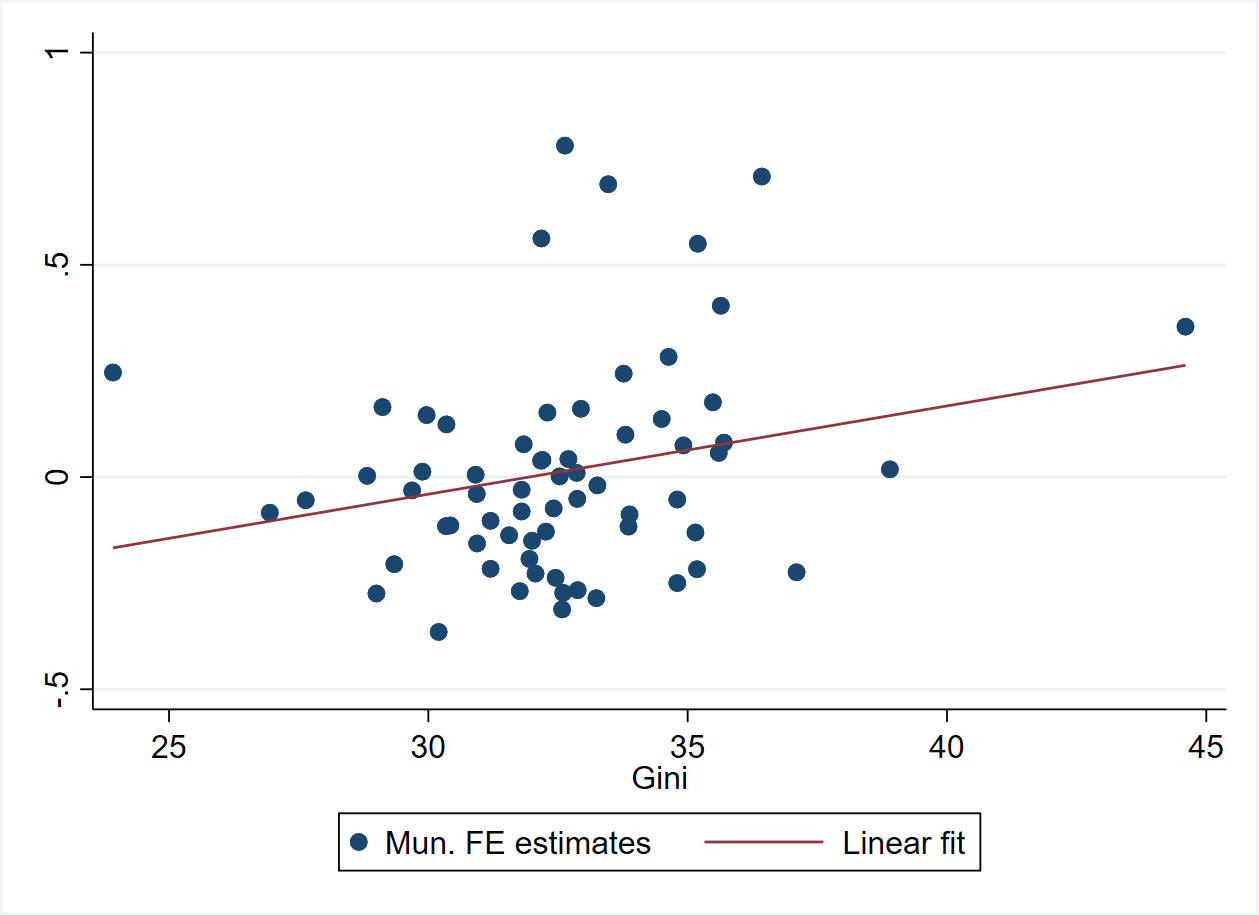


**Figure A22.** Scatter plot of Municipality fixed effects on Gini index in the municipality


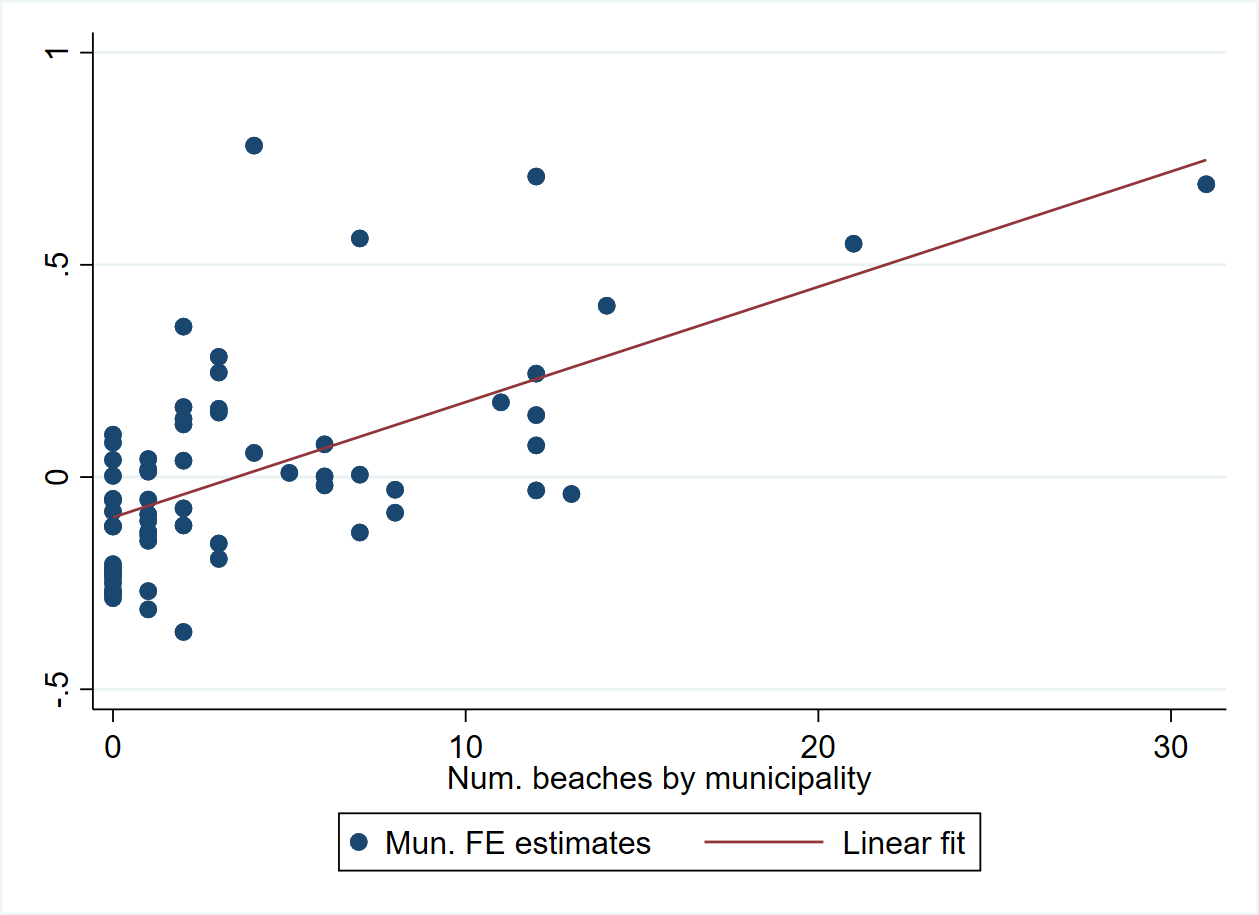


**Figure A23.** Scatter plot of Municipality fixed effects on number of beaches in the municipality


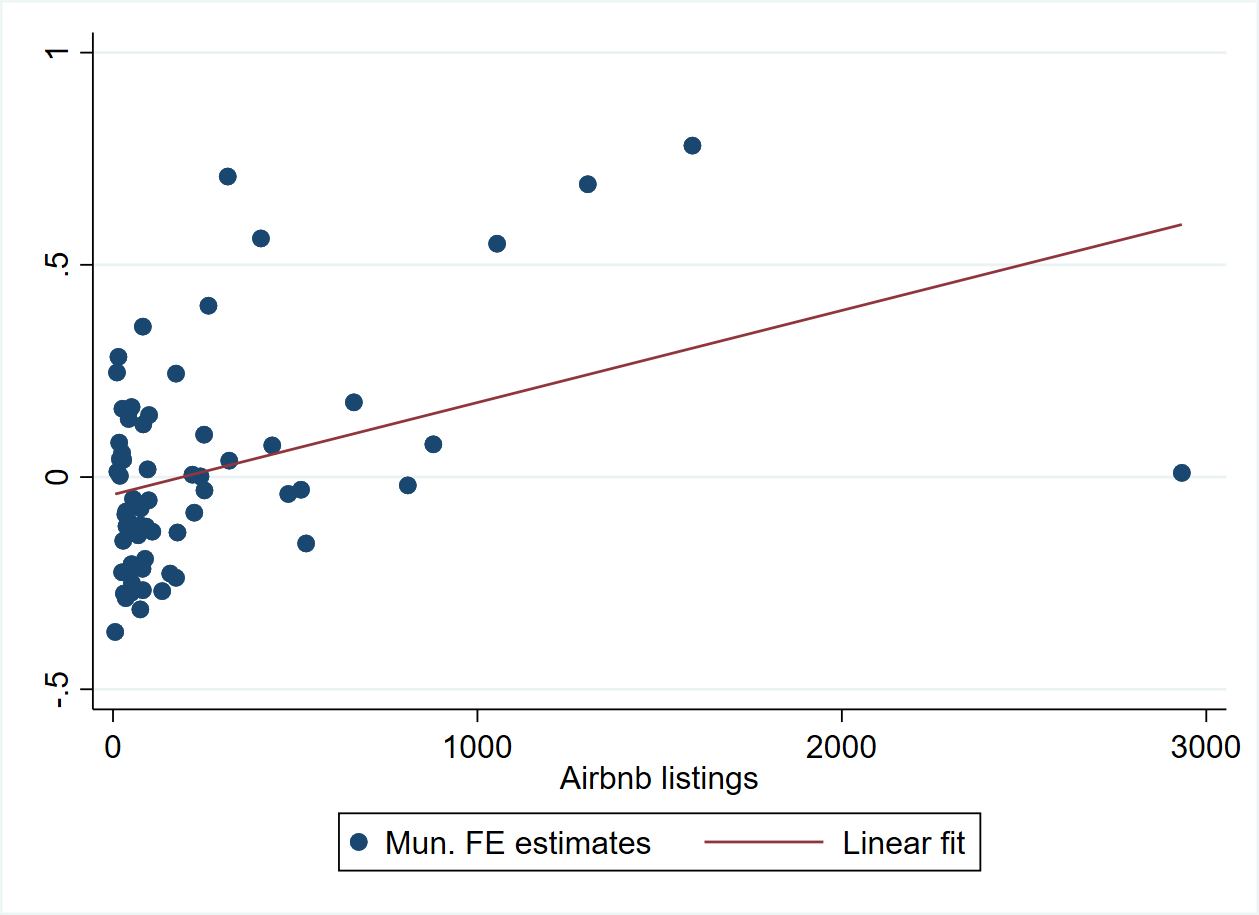


**Figure A24.** Scatter plot of Municipality fixed effects on number of Airbnb listings in the municipality


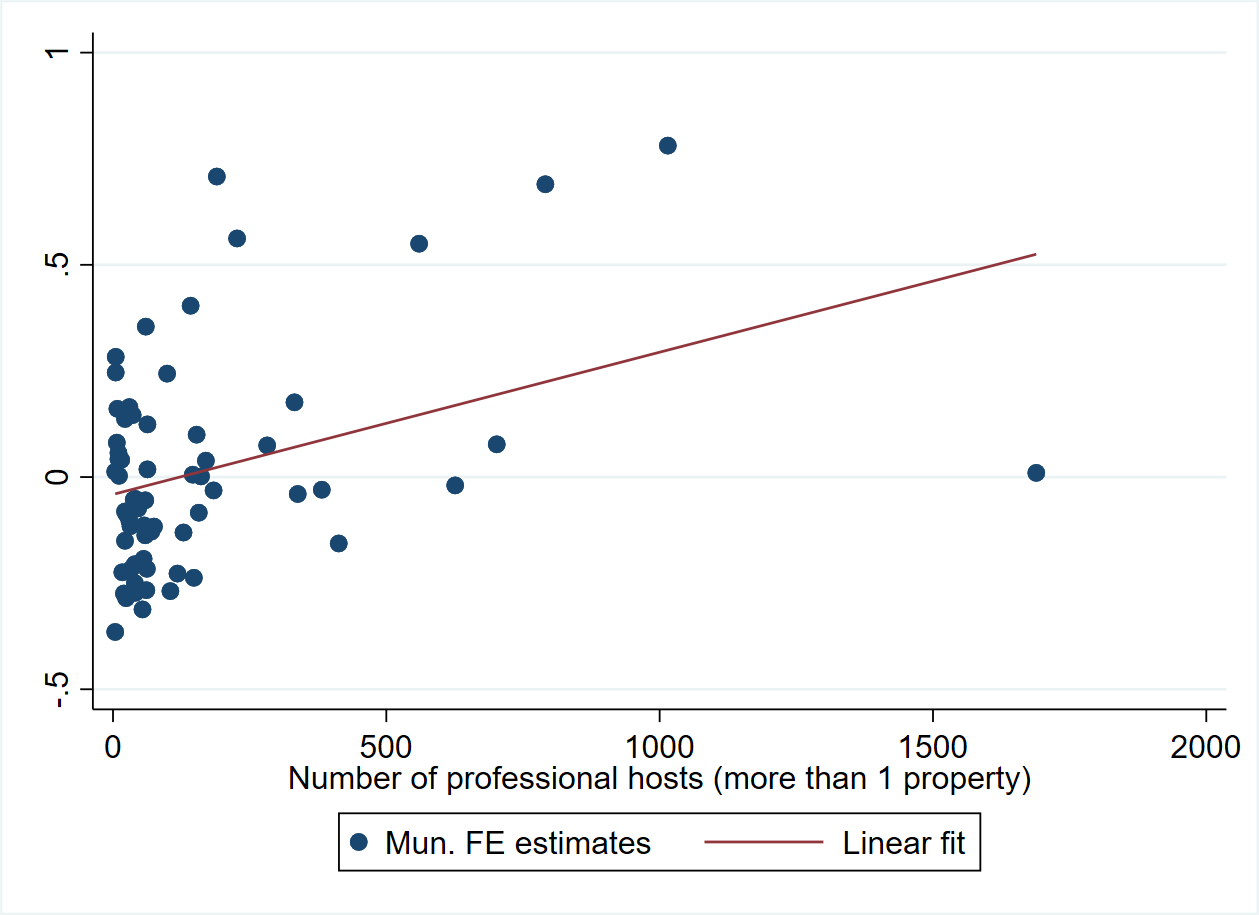


**Figure A25.** Scatter plot of Municipality fixed effects on the number of professional hosts (more than 1 property) in the municipality


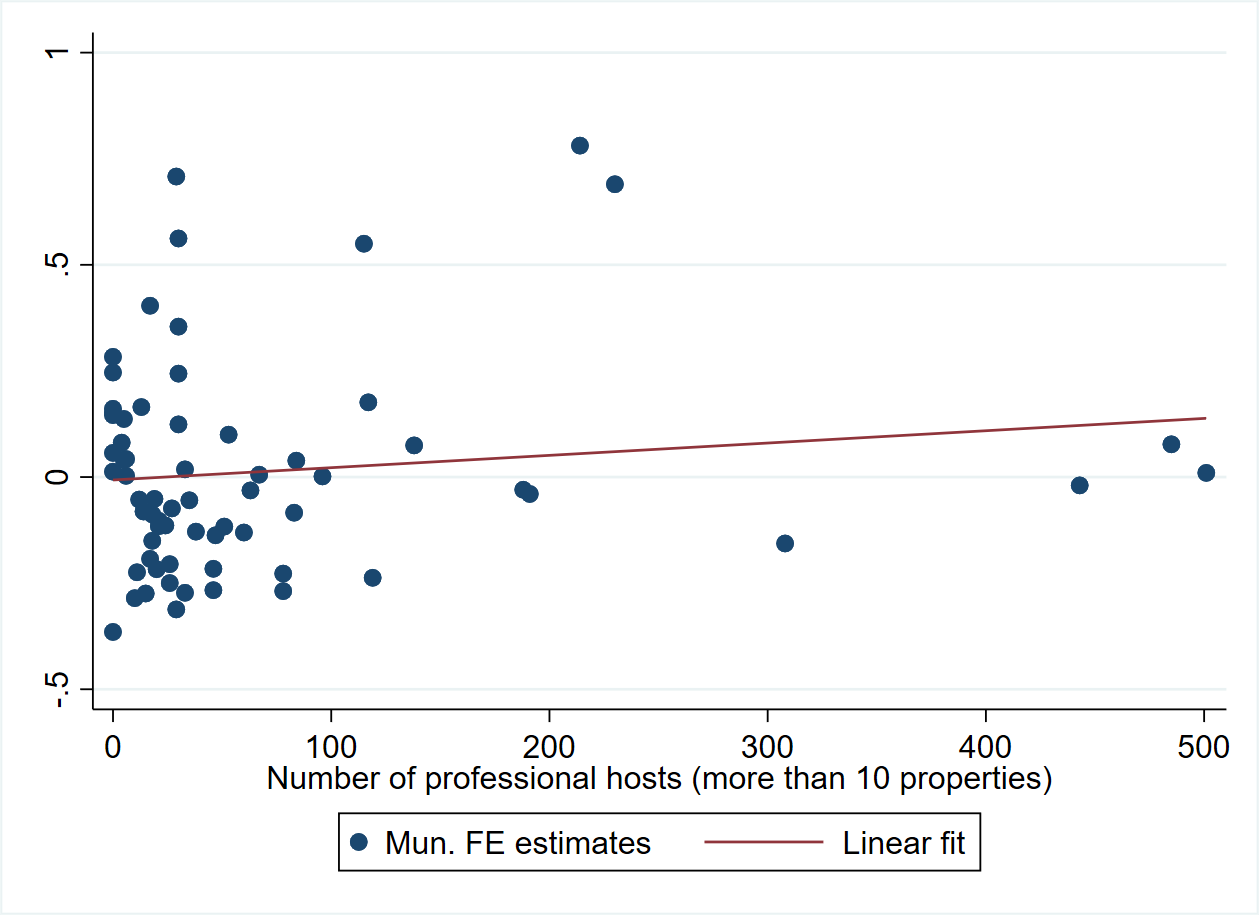


**Figure A26.** Scatter plot of Municipality fixed effects on the number of professional hosts (more than 10 properties) in the municipality
